# A neural mechanism for learning from delayed postingestive feedback

**DOI:** 10.1038/s41586-025-08828-z

**Published:** 2025-04-02

**Authors:** Christopher A. Zimmerman, Scott S. Bolkan, Alejandro Pan-Vazquez, Bichan Wu, Emma F. Keppler, Jordan B. Meares-Garcia, Eartha Mae Guthman, Robert N. Fetcho, Brenna McMannon, Junuk Lee, Austin T. Hoag, Laura A. Lynch, Sanjeev R. Janarthanan, Juan F. López Luna, Adrian G. Bondy, Annegret L. Falkner, Samuel S.-H. Wang, Ilana B. Witten

**Affiliations:** 1 Princeton Neuroscience Institute, Princeton University, Princeton, NJ, USA.; 2 Howard Hughes Medical Institute, Princeton University, Princeton, NJ, USA.

## Abstract

Animals learn the value of foods on the basis of their postingestive effects and thereby develop aversions to foods that are toxic^1–10^ and preferences to those that are nutritious^11–13^. However, it remains unclear how the brain is able to assign credit to flavours experienced during a meal with postingestive feedback signals that can arise after a substantial delay. Here we reveal an unexpected role for the postingestive reactivation of neural flavour representations in this temporal credit-assignment process. To begin, we leverage the fact that mice learn to associate novel^14,15^, but not familiar, flavours with delayed gastrointestinal malaise signals to investigate how the brain represents flavours that support aversive postingestive learning. Analyses of brain-wide activation patterns reveal that a network of amygdala regions is unique in being preferentially activated by novel flavours across every stage of learning (consumption, delayed malaise and memory retrieval). By combining high-density recordings in the amygdala with optogenetic stimulation of malaise-coding hindbrain neurons, we show that delayed malaise signals selectively reactivate flavour representations in the amygdala from a recent meal. The degree of malaise-driven reactivation of individual neurons predicts the strengthening of flavour responses upon memory retrieval, which in turn leads to stabilization of the population-level representation of the recently consumed flavour. By contrast, flavour representations in the amygdala degrade in the absence of unexpected postingestive consequences. Thus, we demonstrate that postingestive reactivation and plasticity of neural flavour representations may support learning from delayed feedback.

Postingestive feedback signals arise from the gut as food is digested and absorbed, and animals are able to associate this delayed feedback with flavours experienced during a meal minutes or hours earlier^1–13^. This learning process is essential for survival—nutritious foods are valuable, whereas poisonous foods can be deadly—but it remains unknown how the brain is able to associate a stimulus (flavour) with a delayed reinforcement signal (postingestive feedback from the gut) that can arrive much later.

Conditioned flavour aversion (CFA) provides a classic example of this credit-assignment problem. Humans^8–10^, rodents^1–5^ and other animals^6,7^ develop CFAs when they experience symptoms of food poisoning (such as gastrointestinal malaise, nausea or diarrhoea), which produces a long-lasting aversion to the potentially poisonous food. Animals can develop a CFA to novel foods after a single pairing (that is, one-shot learning) even with meal-to-malaise delays of several hours^16–18^.

Previous work on CFA has focused on two primary anatomical pathways. The first begins in the mouth and sends taste signals to the gustatory insular cortex^19,20^, which in turn transmits these signals to the basolateral amygdala (BLA)^21,22^. The second begins in the gut and sends malaise signals to a genetically defined population of glutamatergic neurons in the hindbrain parabrachial nucleus (PB) called calcitonin gene-related peptide (CGRP) neurons^23,24^. These malaise-coding CGRP neurons then project to the central amygdala (CEA) and the bed nucleus of the stria terminalis (BST). However, it remains unclear where and how temporally separated flavour and malaise signals ultimately converge in the brain to support learning.

## Novel flavours support one-shot CFA learning

**Fig. 1 |:**
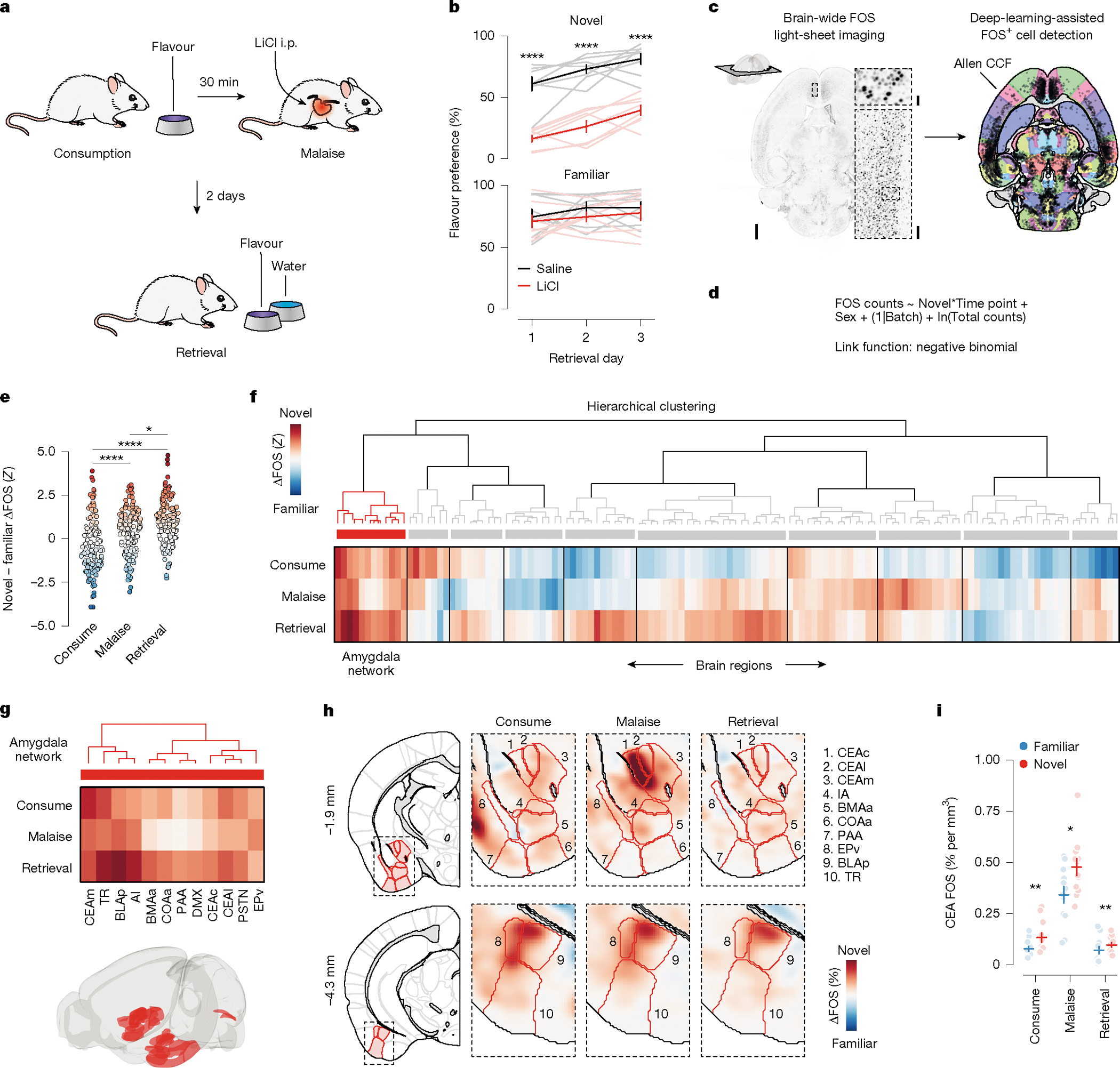
Novel flavours support one-shot, delayed CFA learning and preferentially activate the amygdala at every stage of learning. **a**, Schematic of the CFA paradigm. i.p., intraperitoneal injection. **b**, Flavour preference across three consecutive daily retrieval tests for mice that consumed either a novel (top) or familiar (bottom; all statistical tests not significant (NS)) flavour and then were injected with either LiCl or saline on the conditioning day (*n* = 8 mice per group). The flavour (sweetened grape Kool-Aid) and amount consumed (1.2 ml) was the same for all groups. The group given a familiar flavour was pre-exposed to the flavour on four consecutive days before conditioning, whereas the group given the novel flavour was completely naive. **c**, Example FOS imaging data (100 μm maximum-intensity projection) and cell detection results from the brain-wide light-sheet microscopy imaging pipeline. Scale bars, 1 mm (left), 100 μm (bottom right) and 25 μm (top right). **d**, Description of the GLMM for the brain-wide FOS dataset (*n* = 12 mice per flavour condition per time point for **e**–**i**; see Methods and equation (2)). **e**, Novel – familiar ΔFOS effect distribution at each time point across all significantly modulated brain regions (*n* = 130 brain regions). Each point represents a single brain region. **f**, Hierarchical clustering of novel – familiar ΔFOS effects (see Extended Data Fig. 3d for an expanded version). **g**, Detail of the amygdala network (cluster 1 from **f**) that is preferentially activated by novel flavours at every stage of learning. **h**, Visualization of the difference in FOS^+^ cell density across flavour conditions with Allen CCF boundaries overlaid. **i**, Comparison of individual mice for the novel and familiar flavour conditions for the CEA at each time point. Error bars represent the mean ± s.e.m. *P ≤ 0.05, **P ≤ 0.01, ****P ≤ 0.0001. See Supplementary Table 2 for details of statistical tests and for exact *P* values. See Supplementary Table 1 for a list of brain-region abbreviations and for GLMM statistics.

To gain insight into this long-standing question, we leveraged the fact that novel flavours support CFA after a single pairing with malaise, whereas familiar flavours that are already known to be safe do not^14,15^. Mice consumed a palatable flavour (sweetened grape Kool-Aid) that was either novel (no previous exposure before conditioning) or familiar (four daily pre-exposures before conditioning). Thirty minutes after consumption, the mice were given an intraperitoneal injection of lithium chloride (LiCl) to induce gastrointestinal malaise and related food-poisoning symptoms^18,25^. We then assessed learning 2 days later with a two-bottle memory-retrieval test (Fig. 1a). We used a flavour (combination of taste and odour), rather than a pure taste stimulus, in our study for ethological validity because animals rarely encounter a taste alone, and use both taste and odour to avoid foods that have made them ill^26,27^. Consistent with previous work^14,15^, mice for which the malaise-paired flavour was novel developed a strong and stable aversion, whereas mice for which the same flavour was familiar continued to prefer it to water even after the pairing with malaise (Fig. 1b).

## Brain-wide FOS imaging throughout CFA

**Fig. 2 |:**
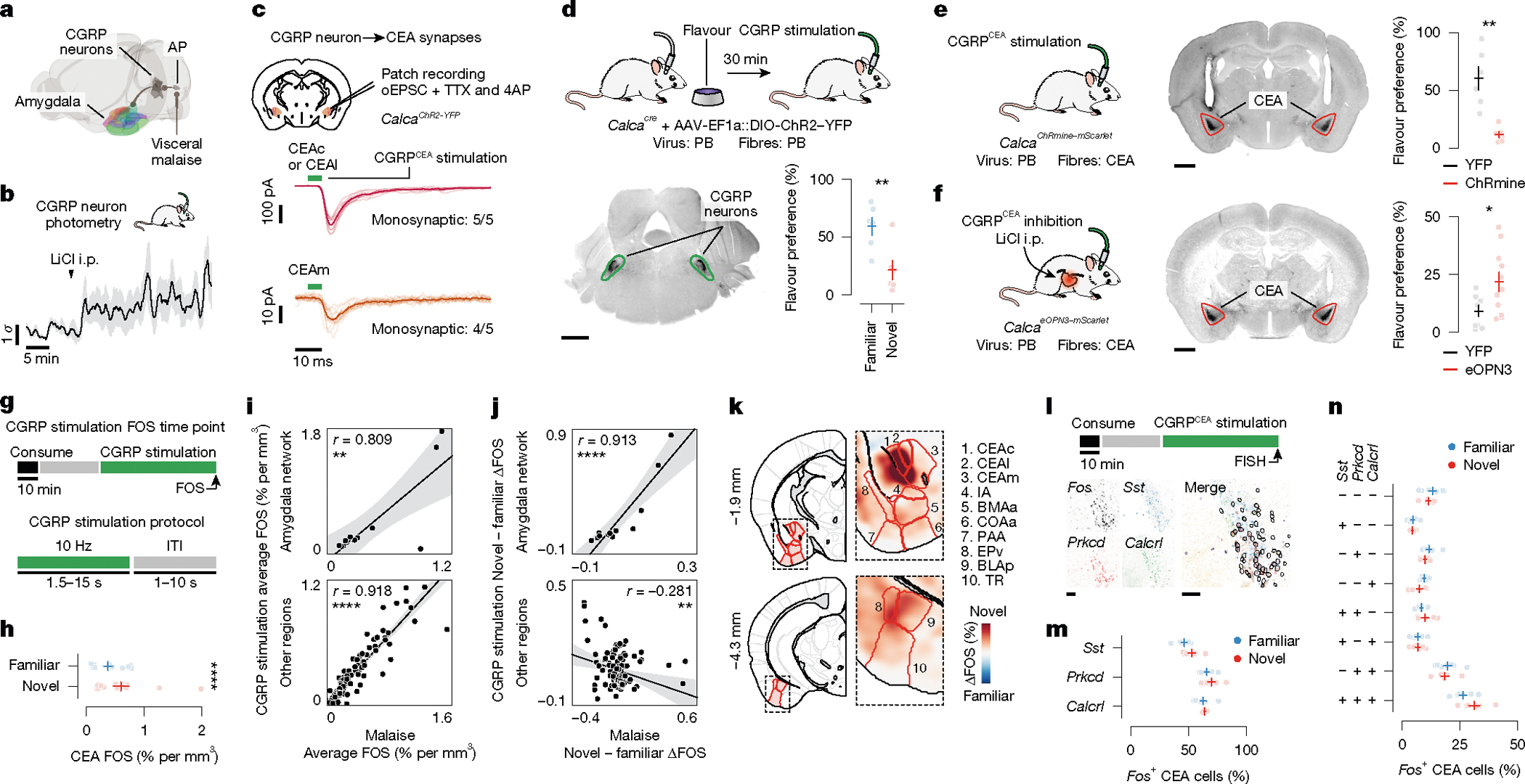
CGRP neurons mediate the effects of postingestive malaise on the amygdala, and monosynaptic connections to the CEA support the acquisition of delayed CFA. **a**, Schematic of the pathway that conveys malaise signals to the amygdala. **b**, CGRP neurons are activated in vivo by LiCl-induced malaise (*n* = 5 mice). **c**, Top, schematic of the slice electrophysiology experiment. Bottom, traces showing strong monosynaptic connections from CGRP neurons to the CEAc and CEAl and weaker connections to the CEAm (*n* = 5 neurons from 3 mice per region). Dark lines represent the average and transparent lines represent individual trials for example neurons. oEPSC, optically evoked excitatory postsynaptic current; TTX, tetrodotoxin; 4AP, 4-aminopyridine. **d**, Top, schematic for the CGRP neuron stimulation experiment. Bottom, example image of ChR2–YFP expression and data for the retrieval test (*n* = 6 mice per group). **e**, Left, schematic of the CGRP^CEA^ projection stimulation experiment. Middle, example image of ChRmine–mScarlet expression. Right, retrieval test data (*n* = 6 mice per group). **f**, Left, schematic of the CGRP^CEA^ projection inhibition experiment. Middle, example image of eOPN3–mScarlet expression. Right, retrieval test data (*n* = 11 mice for eOPN3, 9 mice for YFP). **g**, Schematic of the CGRP neuron stimulation FOS experiment (*n* = 14 mice for novel flavour, 13 mice for familiar flavour for **h**–**k**). ITI, inter-trial interval. **h**, Summary of FOS^+^ cell counts in the CEA for individual mice. **i**, Correlation between average FOS^+^ cell count for LiCl-induced malaise versus CGRP neuron stimulation (*n* = 12 for the amygdala network, 117 for the other regions). **j**, Analogous to **i**, but comparing the difference between flavour conditions. **k**, Visualization of the difference in FOS^+^ cell density across flavour conditions. **l**, Schematic of the FISH experiment. **m**,**n**, Comparison of marker gene expression (**m**) and co-expression (**n**) (*n* = 6 mice for novel flavour, 7 mice for familiar flavour; 490 ± 54 *Fos*^+^ neurons per mouse (mean ± s.e.m.); all statistical tests NS). Error bars represent the mean ± s.e.m. Shaded areas in **b** represent the mean ± s.e.m. and in **i** and **j** represent the linear fit estimate ±95% confidence intervals. Units in **j** are per cent per mm^3^. *P ≤ 0.05, **P ≤ 0.01, ****P ≤ 0.0001. See Supplementary Table 2 for details of statistical tests and for exact *P* values. Scale bars, 1 mm (**d–f**) or 100 μm (**l**).

Using this experimental paradigm, we compared cellular-resolution brain-wide activation levels in response to the same flavour when it was novel versus familiar to determine where novel flavours that support learning are represented and where this representation converges with postingestive malaise signals. After each stage of CFA learning (consumption, malaise and retrieval), brain samples from experimental mice were cleared using the iDISCO+ method^28^ and the samples were then immunolabelled for the immediate-early gene product FOS as a proxy for neural activation. High-resolution and high-signal-to-noise whole-brain light-sheet microscopy imaging volumes were subsequently acquired. We used an automated deep-learning-assisted cell-detection pipeline (258,555 ± 14,421 (mean ± s.e.m.) FOS^+^ neurons per animal across the experiments in Figs. 1 and 2 ; see Methods for details) and registered the location of each FOS^+^ neuron to the Allen Mouse Brain Common Coordinate Framework^29^ (CCF) for downstream analyses (Fig. 1c).

**Extended Data Fig. 1 |:**
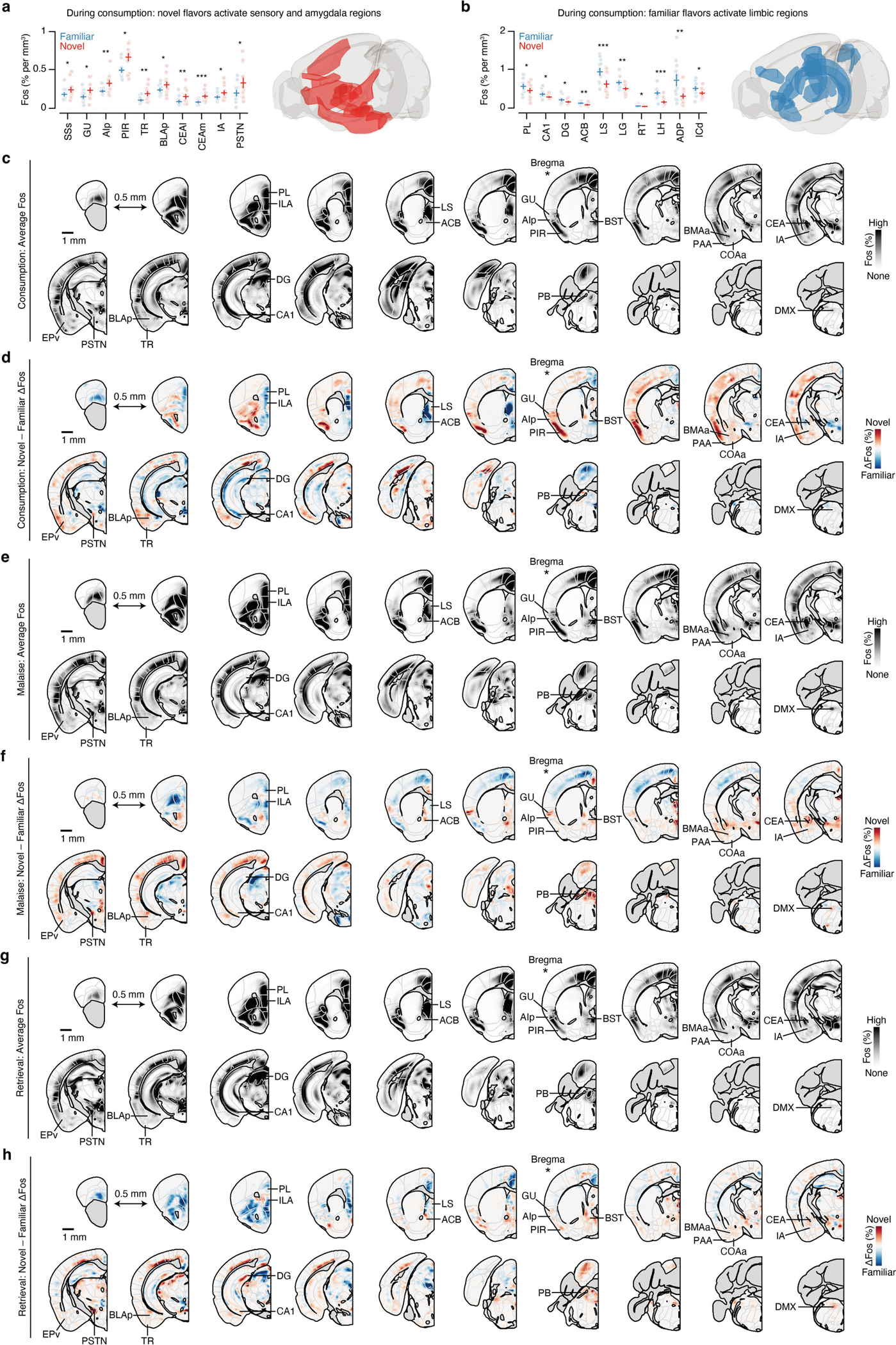
Brain-wide novel versus familiar flavour activation patterns at each stage of one-shot, delayed CFA learning. **a**, Comparison of individual familiar and novel flavour condition mice for every brain region that was significantly novel flavour-activated during consumption (*n* = 12 mice per flavour condition). **b**, Analogous to **a**, but for brain regions that were significantly familiar flavour-activated during consumption (*n* = 12 mice per flavour condition). **c**, Map of average FOS^+^ cell density across all mice for the consumption time point (*n* = 24 mice). The Allen CCF is overlaid. Coronal sections are spaced by 0.5 mm, the section corresponding to Bregma is marked with a *, and key brain regions are labeled. **d**, Map of the difference in average FOS^+^ cell density across novel versus familiar flavour condition mice for the consumption time point (*n* = 12 mice per flavour condition). **e**, Map of average FOS^+^ cell density across all mice for the malaise time point (*n* = 24 mice). **f**, Map of the difference in average FOS^+^ cell density across novel versus familiar flavour condition mice for the malaise time point (*n* = 12 mice per flavour condition). **g**, Map of average FOS^+^ cell density across all mice for the retrieval time point (*n* = 24 mice). **h**, Map of the difference in average FOS^+^ cell density across novel versus familiar flavour condition mice for the retrieval time point (*n* = 12 mice per flavour condition). An interactive visualization of these FOS^+^ cell density maps is available at https://www.brainsharer.org/ng/?id=872. Error bars represent mean ± s.e.m. *P ≤ 0.05, **P ≤ 0.01, ***P ≤ 0.001. See Supplementary Table 2 for details of statistical tests and for exact *P* values. See Supplementary Table 1 for list of brain region abbreviations.

**Extended Data Fig. 2 |:**
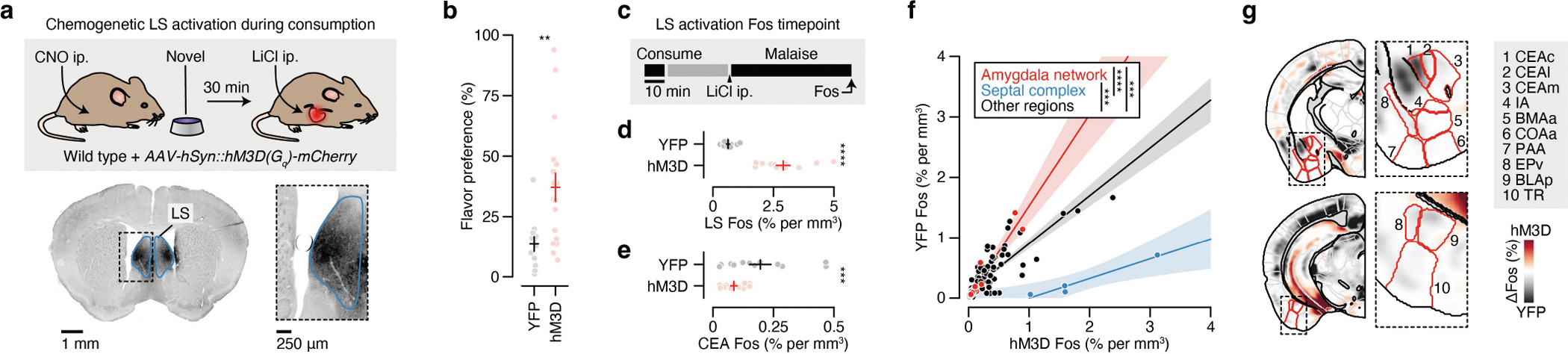
Activation of the LS during novel flavour consumption blocks malaise-driven amygdala activation and interferes with CFA acquisition. **a**, Schematic and example hM3D-mCherry expression data for the bilateral chemogenetic LS activation experiment. CNO was delivered 45-min before the experiment began to ensure that the LS was activated throughout consumption. **b**, Retrieval test flavour preference for the experiment described in **a** (*n* = 18 hM3D mice, 12 YFP mice). **c**, Schematic of the LS activation FOS time point (*n* = 12 mice per group for **d**–**g**). As in **a**, CNO was delivered 45-min before the experiment began, and the flavour was novel for both groups. **d**, Comparison of LS FOS (including the entire ‘Lateral septal complex’ in the Allen CCF) for individual YFP and hM3D mice, confirming strong activation by hM3D. **e**, Comparison of CEA FOS for individual YFP and hM3D mice, showing reduced malaise-driven activation in hM3D mice. **f**, Correlation between the average FOS^+^ cell count of each brain region for hM3D versus YFP mice. The amygdala network (from Fig. 1f, g; *n* = 12 regions), septal complex (*n* = 4 regions), and all other regions (*n* = 114 regions) are shown separately. **g**, Visualization of the difference in FOS^+^ cell density across YFP versus hM3D mice with Allen CCF boundaries overlaid. Error bars represent mean ± s.e.m. Shaded areas represent linear fit estimate ± 95% confidence interval. **P ≤ 0.01, ***P ≤ 0.001, ****P ≤ 0.0001. See Supplementary Table 2 for details of statistical tests and for exact *P* values. See Supplementary Table 1 for list of brain region abbreviations.

We initially investigated the first stage of the CFA paradigm: consumption of flavoured water. We observed marked differences in the brain-wide activation patterns of mice that consumed a novel versus familiar flavour, even though each group had precisely the same sensory experience during FOS induction (Extended Data Fig. 1a–d and Supplementary Table 1; interactive visualization at https://www.brainsharer.org/ng/?id=872, left column). Novel flavours that support learning preferentially activated a set of sensory and amygdala structures (for example, the CEA, the BLA, the insular cortex and the piriform cortex; Extended Data Fig. 1a). These observations are consistent with previous anatomically targeted studies of immediate-early gene expression^30,31^ and with loss-of-function experiments^19–22^ that demonstrated a causal role for many of these regions in CFA. By contrast, a familiar flavour that animals had previously learned was safe primarily engaged a network of limbic regions (for example, the lateral septum (LS), the ventral hippocampus, the prefrontal cortex and the nucleus accumbens; Extended Data Fig. 1b). Unlike the novel-flavour-activated regions, most of these regions had not previously been implicated in CFA. The LS showed the strongest familiar-flavour-dependent activation, and chemogenetic activation of this region during consumption was sufficient to block CFA learning (Extended Data Fig. 2a,b) and amygdala activation by a novel flavour (Extended Data Fig. 2c–g). Together, these results validate the potential of our brain-wide FOS imaging approach to identify new regions that contribute to CFA.

## The amygdala responds to novel flavours

We next investigated how the brain-wide activation patterns induced by a novel versus familiar flavour change during postingestive malaise and, days later, memory retrieval (interactive visualization at https://www.brainsharer.org/ng/?id=872, middle and right columns). We reasoned that preferential novel-flavour activation at these time points, respectively, may reveal where flavour and malaise signals initially converge to support CFA learning and where the CFA memory is stored and recalled. To accurately estimate the contribution of flavour novelty and experimental time point to neural activation in each brain region, we applied a generalized linear mixed model (GLMM) that accounts for variation associated with different technical batches and with the sex of the mice (Fig. 1d, Methods and Supplementary Table 1).

**Extended Data Fig. 3 |:**
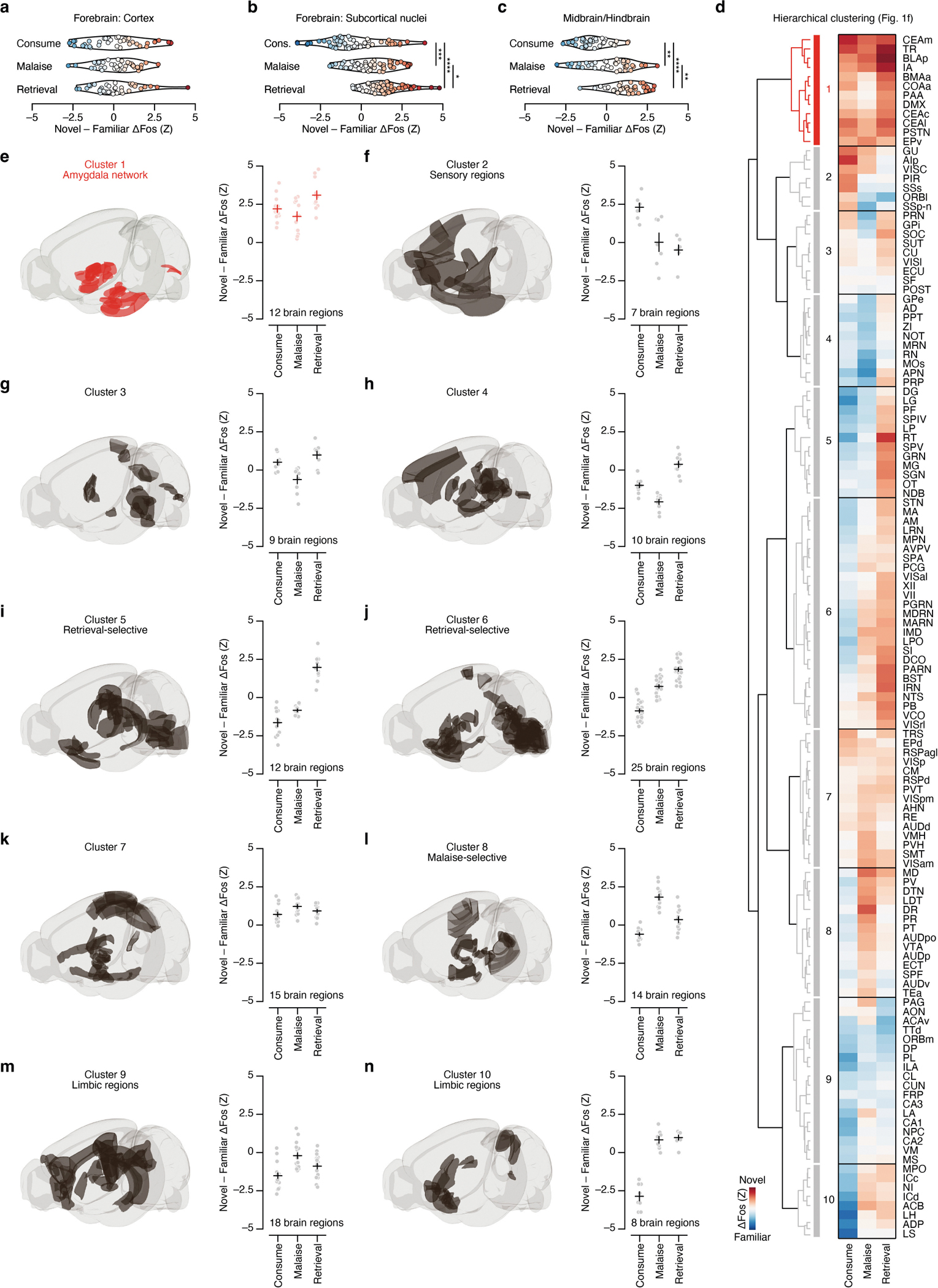
Hierarchical clustering of brain regions based on novel versus familiar flavour activation patterns. Panels **a**–**c** show that the brain-wide shift towards activation by the novel flavour is primarily localized to subcortical regions; outlines represent kernel-density estimates of the empirical distributions. **a**, Novel – familiar ΔFOS effect distribution of all cortical regions (cerebral cortex in the Allen CCF) at each time point (*n* = 38 brain regions; all statistical tests not significant). **b**, Novel – familiar ΔFOS effect distribution of all subcortical forebrain regions (cerebral nuclei, thalamus and hypothalamus in the Allen CCF) at each time point (*n* = 54 brain regions). **c**, Novel – familiar ΔFOS effect distribution of all midbrain and hindbrain regions (midbrain, pons and medulla in the Allen CCF) at each time point (*n* = 38 brain regions). **d**, Hierarchical clustering of novel – familiar ΔFOS effects. This is an expanded version Fig. 1f showing all brain region names. **e**–**n**, Left, Illustration of the brain regions comprising each cluster from the hierarchical clustering analysis. Right, Summary of the novel – familiar ΔFOS effect for each cluster at each time point, showing each brain region as an individual point. Error bars represent mean ± s.e.m. *P ≤ 0.05, **P ≤ 0.01, ***P ≤ 0.001, ****P ≤ 0.0001. See Supplementary Table 2 for details of statistical tests and for exact *P* values. No statistical tests were performed for **e**–**n**. See Supplementary Table 1 for list of brain region abbreviations and for GLMM statistics.

Although novel and familiar flavours preferentially activated an equal fraction of brain regions during consumption (Extended Data Fig. 1a,b), postingestive malaise triggered a brain-wide shift towards activation by the novel flavour (Fig. 1e and Extended Data Fig. 3a–c). This shift towards representing the novel flavour was still present when the memory was retrieved days later (Fig. 1e and Extended Data Fig. 3a–c).

**Extended Data Fig. 4 |:**
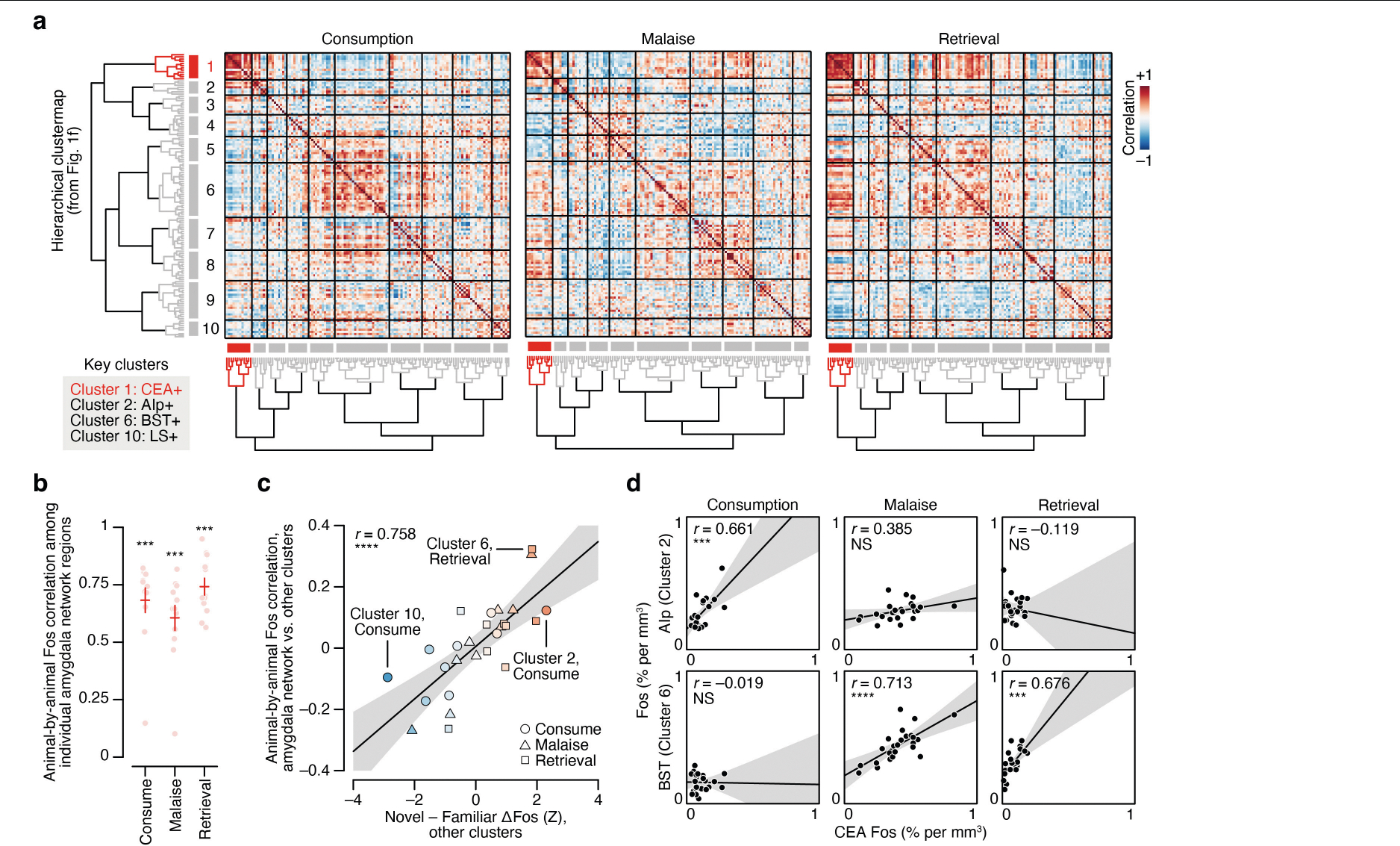
The amygdala cluster forms a functional network. **a**, Correlation matrices showing the animal-by-animal pairwise FOS correlation for every pair of brain regions during consumption (left), delayed malaise (middle), or memory retrieval (right). Brain regions are sorted using the hierarchical clustermap obtained from the novel – familiar ΔFOS effects in Fig. 1f. **b**, Summary of the average within-cluster FOS correlation for individual amygdala network regions (Cluster 1 from Fig. 1f, g) by time point (*n* = 12 regions). The high animal-by-animal correlation among all of the regions in cluster 1 suggest that these regions form a functional network. Panels **c,d** show that activation of other clusters of brain regions is more correlated with amygdala network activation at experimental time points when those clusters are more strongly novel flavour-selective, including for clusters that were specifically engaged during the initial flavour consumption (comprising sensory cortices; cluster 2) or during retrieval (including the BST; cluster 6). **c**, Summary of the average across-cluster FOS correlation between the amygdala network and every other cluster at each time point as a function of the other cluster’s standardized novel – familiar effect at that time point (*n* = 9 clusters × 3 time points). **d**, Scatter plots showing the pairwise correlation between AIp (top; example cluster 2 region) or BST (bottom; example cluster 6 region) and the CEA (*n* = 24 mice per time point) at each experimental time point. Error bars represent mean ± s.e.m. Shaded areas represent linear fit estimate ± 95% confidence interval. NS, not significant, ***P ≤ 0.001, ****P ≤ 0.0001. See Supplementary Table 2 for details of statistical tests and for exact *P* values. See Supplementary Table 1 for list of brain region abbreviations.

To investigate which brain regions contributed to this effect, we performed hierarchical clustering on weights from the GLMM of novel-flavour versus familiar-flavour activation across experimental time points (Fig. 1f and Extended Data Fig. 3d–n). This analysis uncovered a network of amygdala regions (cluster 1) that was preferentially activated by the novel flavour at every stage of learning (Fig. 1g,h and see Extended Data Fig. 4a–d for correlation analyses within and across clusters), most notably in the CEA (Fig. 1i).

This discovery is notable for two reasons. First, the fact that the representation of a novel flavour formed during the initial consumption stage is still present 30 min later during postingestive malaise implies that this network is a site for the convergence of flavour and malaise signals. Second, the observation that this novel-flavour representation is still present upon memory retrieval suggests that the same network is also a site of storage and recall.

## CGRP neurons mediate the effects of malaise

**Extended Data Fig. 5 |:**
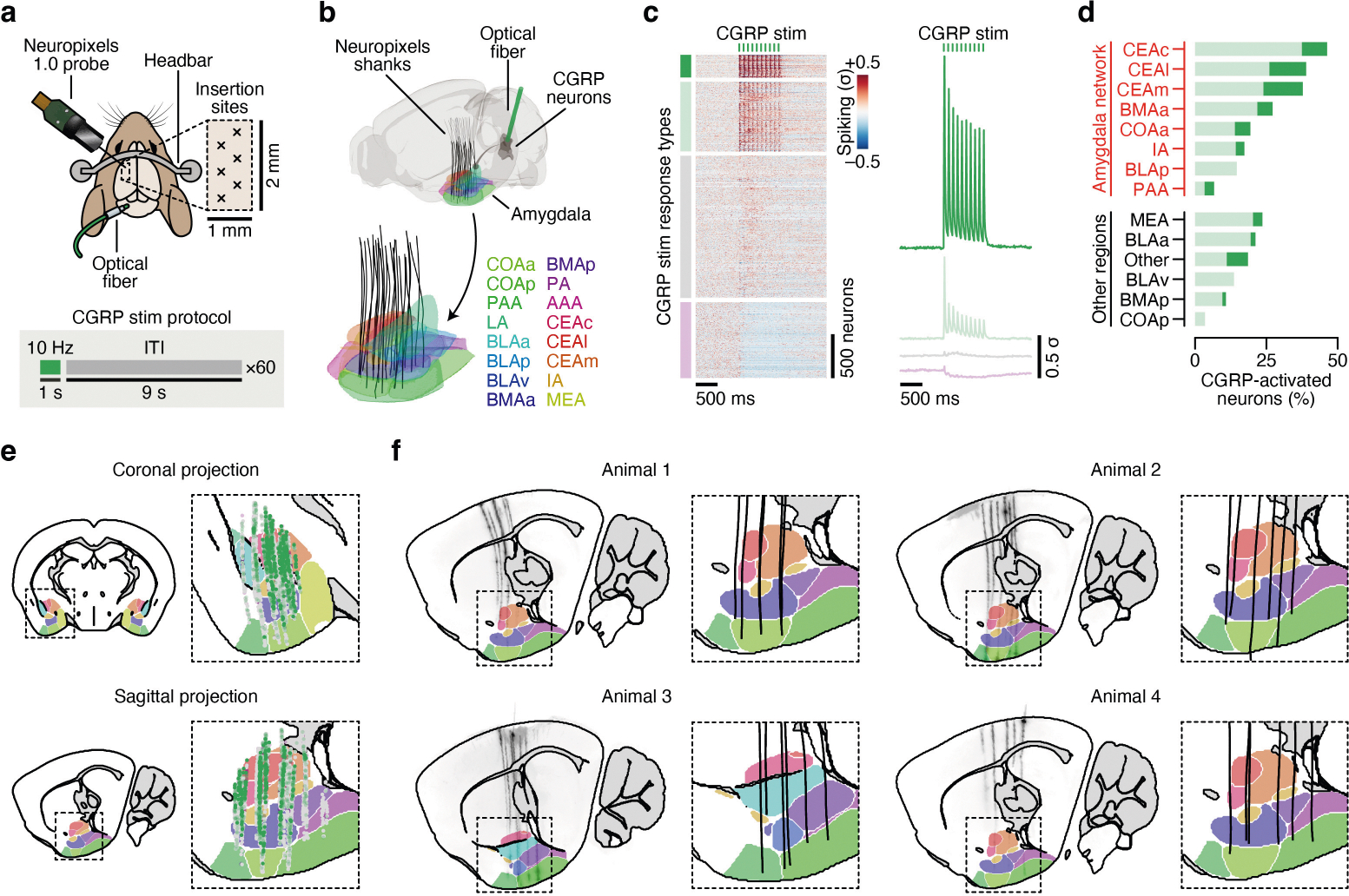
An electrophysiological atlas of the effects of CGRP neuron stimulation on amygdala activity in vivo. **a**, Schematic of the acute Neuropixels recording experiment (created using BioRender.com). **b**, Reconstruction of recording trajectories registered to the Allen CCF. Each line represents one insertion of a single-shank Neuropixels 1.0 probe (*n* = 24 insertions from 4 mice). **c**, Left, PETHs of neural activity time-locked to CGRP neuron stimulation trains (*n* = 3,524 amygdala neurons from 24 insertions). Neurons were divided into four response types using a GMM (see Methods): two CGRP neuron stimulation-activated response types (7.3% strongly activated, dark green; 22.4% weakly activated, light green), one CGRP neuron stimulation-inhibited response type (24.6%, purple), and one unmodulated response type (45.8%, gray). Right, Average PETHs for each GMM response type. **d**, Percentage of recorded neurons that were CGRP neuron stimulation-activated based on the GMM across amygdala subregions (*n* = 339 CEAc, 272 CEAl, 717 CEAm, 526 BMAa, 129 COAa, 133 IA, 41 BLAp, 30 PAA, 182 MEA, 354 BLAa, 54 Other (AAA, LA, PA), 44 BLAv, 250 BMAp, and 58 COAp neurons). Regions in the CFA amygdala network (cluster 1 from Fig. 1f, g) are shown in red, and other amygdala regions are shown in black. **e**, Anatomical distribution of all CGRP neuron stimulation-activated (green), CGRP neuron stimulation-inhibited (purple), and unmodulated (gray) neurons projected onto a single coronal or sagittal section of the Allen CCF. **f**, Left, Light-sheet microscopy data for each animal showing Neuropixels probe trajectories aligned to the Allen CCF with amygdala subregions overlaid. Right, Reconstructions of recording trajectories. For each animal, a single sagittal section corresponding to the center-of-mass of the active recording sites is shown. The colormap for amygdala regions in **b** is also used in **e**,**f**. See Supplementary Table 1 for list of brain region abbreviations.

The idea that the amygdala could be a crucial node for the convergence of flavour representations and malaise signals is further supported by classic work establishing the amygdala as a site of associative learning^32,33^. Further pointing to the amygdala as a site of convergence, parabrachial CGRP neurons in the brainstem are reported to convey visceral malaise signals to the CEA^23,24,34^ (Fig. 2a). Indeed, we found that LiCl injection activated CGRP neurons in vivo^34^ (Fig. 2b). CGRP neurons formed dense monosynaptic connections in the CEA^34^ (Fig. 2c; latency: 5.9 ± 0.3 ms (mean ± s.e.m.)), and stimulation of CGRP neurons activated neurons throughout the amygdala in vivo (Extended Data Fig. 5a–f).

Optogenetic stimulation of CGRP neurons also recapitulated the effects of LiCl-induced malaise in mediating novel-flavour-dependent delayed CFA. Specifically, stimulation of CGRP neuron cell bodies that began 30 min after flavour consumption was sufficient to replace LiCl injection and condition an aversion to a novel but not familiar flavour (Fig. 2d). Similarly, stimulation of CGRP neuron→CEA (CGRP^CEA^) axon terminals 30 min after consumption of a novel flavour was also sufficient to condition a strong CFA (Fig. 2e). CGRP^CEA^ projection inhibition during delayed LiCl-induced malaise significantly interfered with CFA acquisition (Fig. 2f), but did not fully block it. This result is consistent with previous work showing that other CGRP neuron projections, for example to the BST^24^, also contribute to CFA.

**Extended Data Fig. 6 |:**
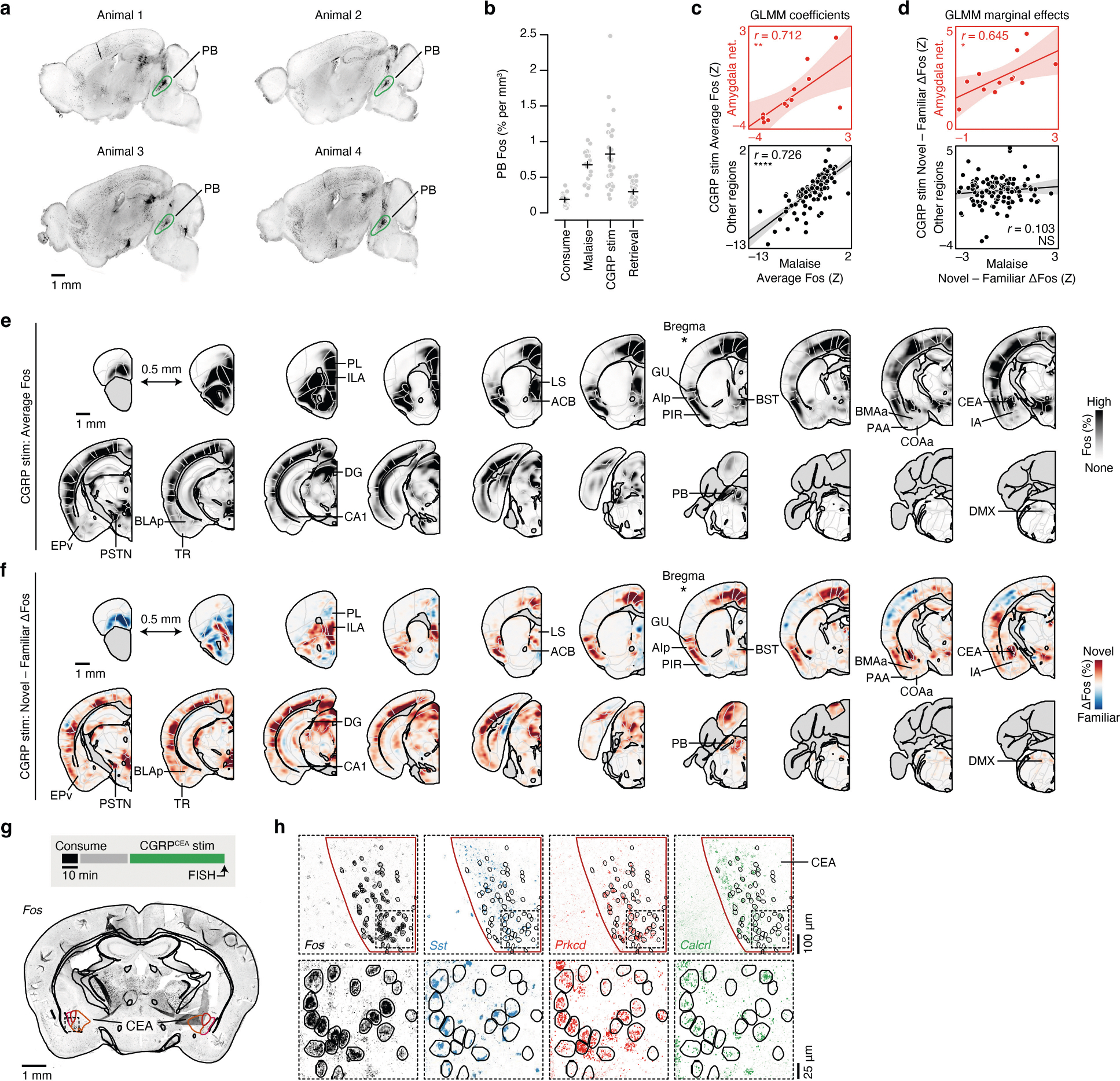
Brain-wide novel versus familiar flavour activation pattern during CGRP neuron stimulation. **a**, Example brain-wide FOS imaging data (200-μm maximum-intensity projections) for four example CGRP neuron stimulation animals. **b**, Summary of FOS^+^ cell counts in the PB at each time point (*n* = 24 consumption, 24 malaise, 27 CGRP neuron stimulation, 24 retrieval mice). **c**, Analysis analogous to Fig. 2i but using a GLMM, showing the correlation among the standardized coefficients for the main LiCl-induced malaise and CGRP neuron stimulation effects from Equation 3 (*n* = 12 amygdala, 117 other regions). **d**, Analysis analogous to Fig. 2j but using a GLMM, showing the correlation among the average marginal effects of flavour from Equation 4 (*n* = 12 amygdala, 117 other regions). **e**, Map of average FOS^+^ cell density across all mice for the CGRP neuron stimulation time point (*n* = 27 mice). **f**, Map of the difference in average FOS^+^ cell density across novel versus familiar flavour condition mice for the CGRP neuron stimulation time point (*n* = 14 novel flavour mice, 13 familiar flavour mice). **g**, Top, Schematic of the CGRP^CEA^ projection stimulation RNAscope FISH experiment. Bottom, Example slide scanner image of FOS expression with the Allen CCF overlaid. **h**, Example confocal image showing *Fos*, *Sst*, *Prkcd*, and *Calcrl* expression. This is an expanded version of Fig. 2l. The top row shows the full field-of-view, and the bottom row is magnified with *Fos*^+^ cell outlines overlaid in black. Error bars represent mean ± s.e.m. Shaded areas represent linear fit estimate ± 95% confidence interval. NS, not significant, *P ≤ 0.05, **P ≤ 0.01, ****P ≤ 0.0001. See Supplementary Table 2 for details of statistical tests and for exact *P* values. No statistical tests were performed for **b**. See Supplementary Table 1 for list of brain region abbreviations and for GLMM statistics.

Given the similarities between LiCl-induced malaise and CGRP neuron stimulation, we sought to determine whether postingestive stimulation of these cells could recapitulate the effects of LiCl-induced malaise on neural activation in the amygdala and across the brain (Fig. 2g and Extended Data Fig. 6a,b). Postingestive CGRP neuron stimulation produced highly similar levels of overall neural activation (FOS^+^ cell counts in individual brain regions) across the entire brain compared to LiCl-induced malaise (Fig. 2i and Extended Data Fig. 6c,e). Also similar to LiCl-induced malaise, CGRP neuron stimulation induced stronger activation of the amygdala network when preceded by consumption of a novel rather than familiar flavour (Fig. 2j,k and Extended Data Fig. 6d,f). This effect was particularly prominent in the CEA (Fig. 2h) and was not observed in brain regions outside the amygdala network (Fig. 2j and Extended Data Fig. 6d,f).

We next asked whether the stronger effect of CGRP neuron stimulation on amygdala activation after consumption of a novel versus familiar flavour could be explained by recruitment of a specific CEA cell type. To address this question, we performed multiplex fluorescence in situ hybridization (FISH) in the CEA in a separate group of mice that consumed either a novel or familiar flavour followed by delayed CGRP^CEA^ projection stimulation. We then examined co-expression of *Fos* mRNA with markers for several known CEA cell types^35,36^ (*Sst*, *Prkcd* and *Calcrl*; Fig. 2l). Most cells that expressed *Fos* also expressed *Prkcd* or *Calcrl*, and this did not depend on whether the mice had consumed a novel or familiar flavour before CGRP^CEA^ projection stimulation (Fig. 2m,n and Extended Data Fig. 6g,h).

Taken together, these experiments show that postingestive CGRP neuron activity is necessary and sufficient to mediate delayed CFA learning and the effects of malaise on novel-flavour-dependent, amygdala-specific neural activation. This novelty-dependent activation does not seem to be instantiated by a specific CEA cell type. Instead, novel (versus familiar) flavour consumption leads to a greater number of *Prkcd*^+^ and *Calcrl*^+^ CEA neurons being activated by subsequent CGRP neuron activity.

## Malaise reactivates flavour-coding neurons

**Fig. 3 |:**
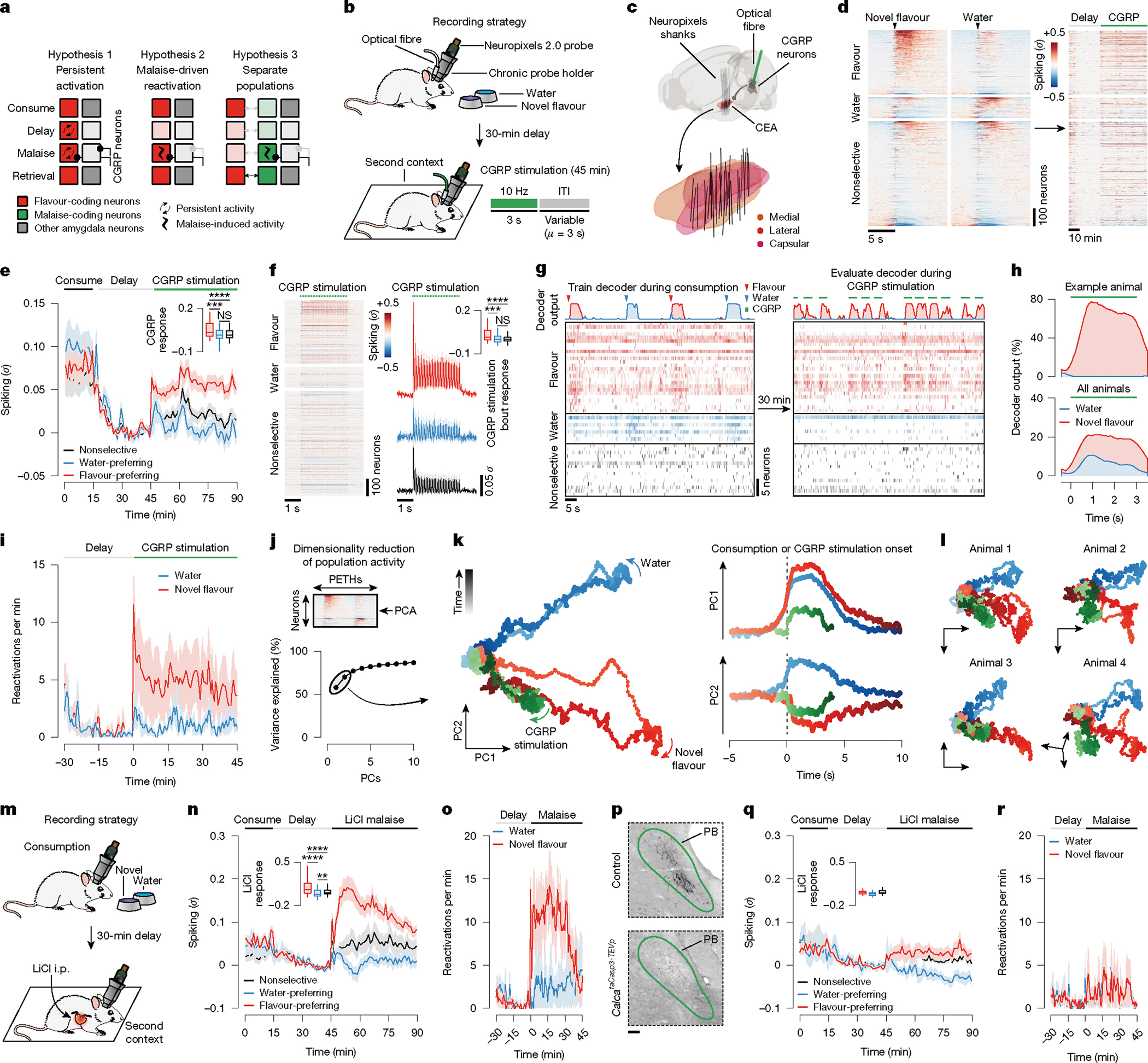
Postingestive CGRP neuron activity preferentially reactivates the representation of a recently consumed flavour in the amygdala. **a**, Hypotheses for how the amygdala associates temporally separated flavour and malaise signals. **b**, Schematic of the CGRP neuron-stimulation recording strategy. **c**, Reconstruction of recording trajectories registered to the Allen CCF. Each line represents one shank of a four-shank Neuropixels 2.0 probe (32 shanks from 8 mice). **d**, Average spiking of all individual neurons (*n* = 1,104 single units and multiunits from 8 mice). **e**, Average spiking of novel-flavour-preferring (*n* = 373), water-preferring (*n* = 121) and nonselective (*n* = 610) populations. **f**, Left, average spiking of individual neurons during CGRP neuron-stimulation bouts. Right, population averages (same sample sizes as **e**). **g**, Example of a multinomial logistic regression decoder session. **h**, Average decoder posterior time locked to CGRP neuron stimulation (mean across 6 mice). **i**, Average reactivation rates of novel flavour or water representations (*n* = 6 mice). **j**, PCA schematic. **k**, Population trajectories for novel-flavour consumption, water consumption and CGRP neuron stimulation. **l**, Trajectories for individual example mice. **m**, Schematic of the LiCl-induced-malaise recording strategy. **n**, Average spiking of novel-flavour-preferring (*n* = 280 neurons from 4 mice), water-preferring (*n* = 80) and nonselective (*n* = 218) populations. **o**, Average reactivation rates of novel flavour or water representations (*n* = 4 mice). **p**, Example CGRP immunoreactivity data confirming the ablation of CGRP neurons in the PB (outlined in green). Scale bar, 100 μm. **q**, Analogous to **n**, but for mice in which CGRP neurons were ablated (*n* = 124 novel-flavour-preferring, 20 water-preferring, 256 nonselective neurons from 4 mice; all statistical tests NS). **r**, Analogous to **o**, but for mice in which CGRP neurons were ablated (*n* = 4 mice). Shaded areas represent the mean ± s.e.m. Inset box plots show the 10th, 25th, 50th, 75th and 90th percentiles. **P ≤ 0.01, ***P ≤ 0.001, ****P ≤ 0.0001. See Supplementary Table 2 for details of statistical tests and for exact *P* values.

Our brain-wide FOS measurements and CGRP neuron manipulations point to the amygdala as a unique site for the convergence of flavour representations and delayed malaise signals. However, these experiments do not resolve how these temporally separated signals are integrated at the single-cell level. One possibility is that individual novel-flavour-coding neurons may be persistently activated long after a meal in a manner that provides passive overlap with delayed CGRP neuron malaise signals (hypothesis 1 in Fig. 3a). Alternatively, CGRP neuron inputs may specifically reactivate novel-flavour-coding neurons (hypothesis 2 in Fig. 3a). Another possibility is that CGRP neuron inputs may activate a separate population of amygdala neurons that subsequently become incorporated into the novel-flavour representation during memory consolidation (hypothesis 3 in Fig. 3a). Testing these hypotheses requires tracking the activity of the same neurons across the stages of learning.

**Extended Data Fig. 7 |:**
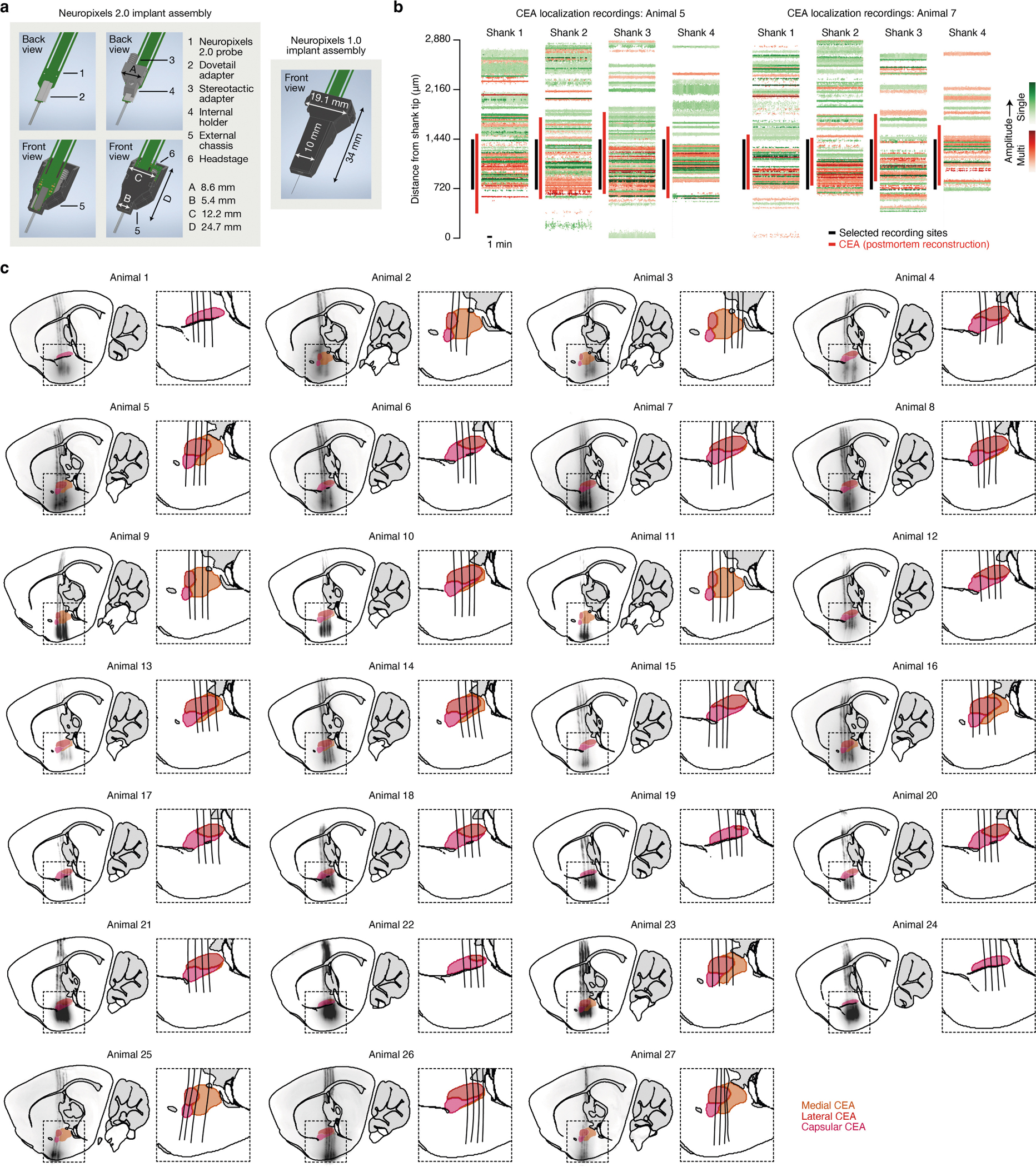
Chronic Neuropixels electrophysiology in the amygdala of freely moving mice. **a**, Left, Schematic illustration of the chronic Neuropixels 2.0 implant assembly at progressive stages of construction from top left to bottom right. Right, Schematic illustration of the chronic Neuropixels 1.0 implant assembly^68^ upon which the 2.0 implant design is based, shown for size comparison. **b**, Test recordings used to select recording sites (black bars) properly targeting the CEA (red bars, based on postmortem reconstruction) for two example animals. The shanks are arranged from anterior (1, left) to posterior (4, right) and span 750-μm total. We found that we could distinguish the CEA from nearby brain regions along the vertical axis of the probe shanks as a band of dense neural activity. **c**, Left, Light-sheet microscopy data for each animal showing Neuropixels shank trajectories aligned to the Allen CCF with CEA subregions overlaid. Right, Reconstruction of trajectories in the CEA. For each animal, a single sagittal section corresponding to the center-of-mass of all active recording sites is shown. See Methods for a description of which animals were included in each experiment.

**Extended Data Fig. 8 |:**
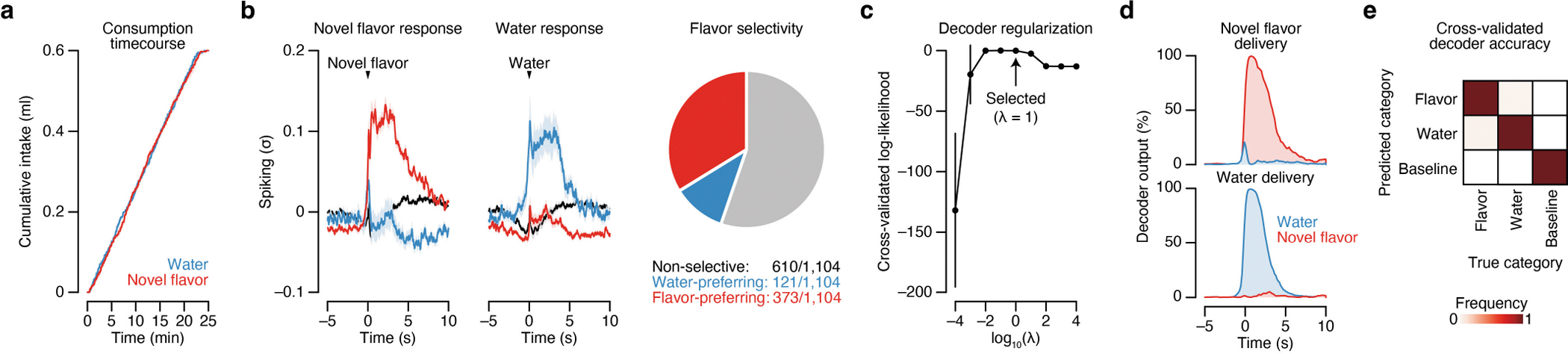
The amygdala is strongly activated by a novel flavour and near-perfectly discriminates flavour. **a**, Cumulative intake of the novel flavour and water in the two-reward CFA paradigm in Fig. 3b (*n* = 8 mice). We used a randomized, trial-based structure to ensure that mice consumed the two options at an equal rate (see Methods). **b**, Left, PETHs to novel flavour delivery for the novel flavour-preferring (*n* = 373 neurons from 8 mice), water-preferring (*n* = 121 neurons), and nonselective (*n* = 610 neurons) CEA neurons in Fig. 3c–l. Middle, PETHs to water delivery for the same novel flavour-preferring, water-preferring, and nonselective CEA neurons. Right, Pie chart visualizing the proportion of novel flavour-preferring, water-preferring, and nonselective CEA neurons. Panels **c–e** provide additional characterization of the multinomial logistic regression decoder using CEA population activity in Fig. 4g–i. **c**, Cross-validated log-likelihood for the decoder classifying periods of novel flavour consumption versus water consumption versus baseline activity across a range of regularization parameter (λ) values (*n* = 6 mice). **d**, Decoder output time-locked to novel flavour delivery (top) and water delivery (bottom) in the initial consumption period (mean across 6 mice). The decoder’s predicted probability for novel flavour consumption and water consumption are both shown. **e**, Confusion matrix summarizing the cross-validated decoder performance across all mice (*n* = 6 mice). The overall misclassification rate was 0.56% (4 out of 720). Error bars and shaded areas represent mean ± s.e.m. No statistical tests were performed for **c**.

**Extended Data Fig. 9 |:**
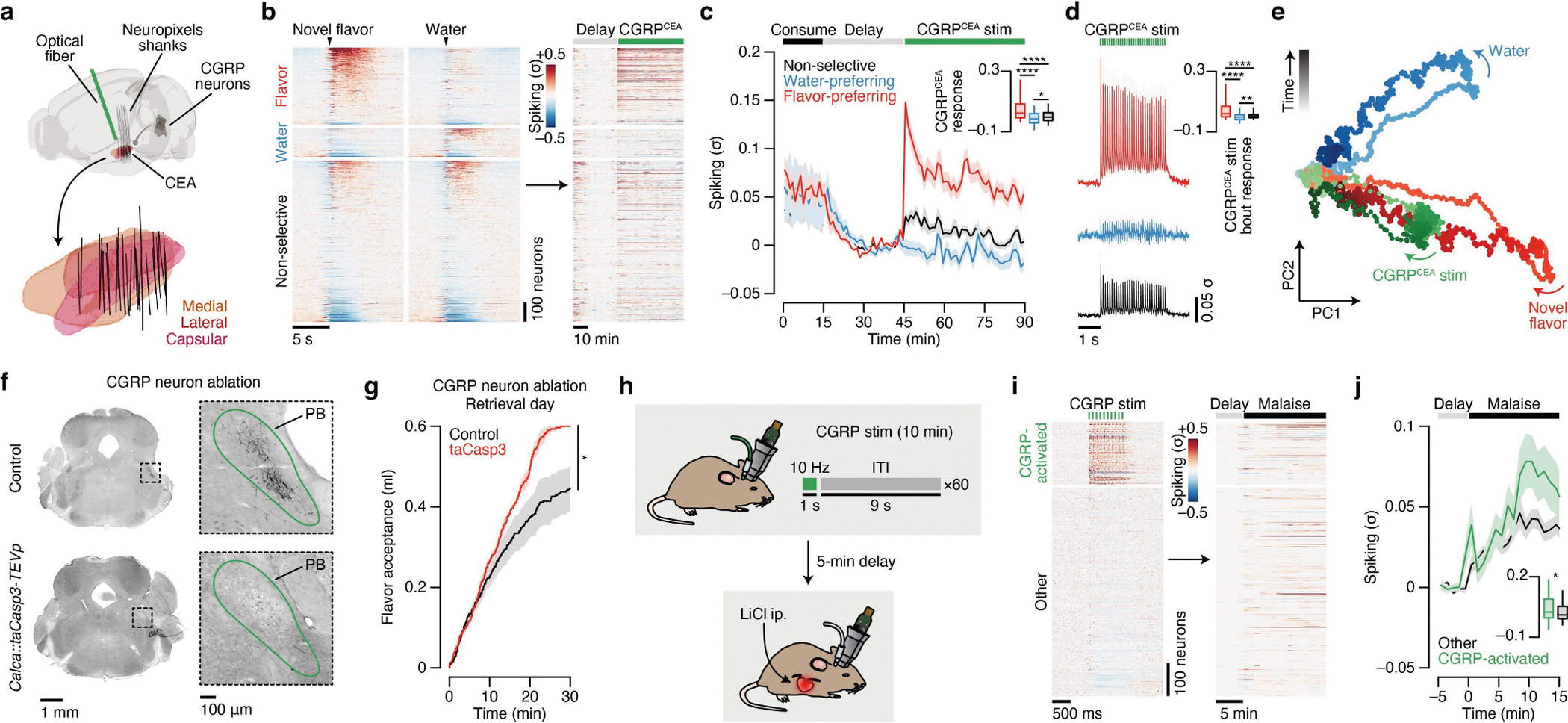
CGRP^CEA^ projection stimulation reactivates flavour representations in the amygdala, and CGRP neuron ablation impairs delayed CFA learning. Panels **a**–**e** show that CGRP^CEA^ projection stimulation reactivates flavour representations in the amygdala. **a**, Reconstruction of recording trajectories registered to the Allen CCF for mice with CGRP^CEA^ projection stimulation (*n* = 32 shanks from 8 mice). **b**, Average spiking of individual neurons during the CGRP^CEA^ projection stimulation conditioning experiment (*n* = 1,221 neurons from 8 mice). **c**, Average spiking of the novel flavour-preferring (*n* = 354 neurons), water-preferring (*n* = 129 neurons), and nonselective (*n* = 738 neurons) populations across the entire experiment. **d**, Average spiking of the novel flavour-preferring, water-preferring, and nonselective populations during individual bouts of CGRP^CEA^ projection stimulation (same sample sizes as **c**). **e**, Neural trajectories in PC-space for novel flavour consumption, water consumption, and CGRP^CEA^ projection stimulation. Panels **f**,**g** show that genetic ablation of CGRP neurons by taCasp3-TEVp impairs delayed CFA learning. **f**, Example CGRP immunoreactivity data confirming genetic ablation of CGRP neurons by taCasp3-TEVp. This is an expanded version of Fig. 3p. **g**, CGRP neuron ablation mice show significantly higher acceptance of the conditioned flavour following LiCl-induced CFA when compared to wild type controls (*n* = 6 taCasp3 mice, 7 control mice). Panels **h**–**j** show that CGRP neuron stimulation-activated CEA neurons are also activated by LiCl injection. **h**, Schematic. **i**, Average spiking during CGRP neuron stimulation and then during LiCl-induced malaise (*n* = 821 neurons from 4 mice). **j**, Average spiking of the CGRP neuron stimulation-activated neurons (*n* = 189 neurons) and other neurons (*n* = 632 neurons) during LiCl-induced malaise. Shaded areas represent mean ± s.e.m. Inset box plots show the 10^th^, 25^th^, 50^th^, 75^th^, and 90^th^ percentiles. *P ≤ 0.05, **P ≤ 0.01, ****P ≤ 0.0001. See Supplementary Table 2 for details of statistical tests and for exact *P* values.

Therefore, to distinguish these possibilities, we performed high-density recordings of individual neurons in the CEA—the core node in the amygdala network from our brain-wide FOS imaging dataset (Figs. 1 and 2)—during consumption, subsequent malaise and memory retrieval (Fig. 3b). Recordings were performed with chronically implanted four-shank Neuropixels 2.0 probes^37^ (Extended Data Fig. 7a). Reconstruction of individual shank trajectories confirmed that we were able to precisely target the CEA (Fig. 3c and Extended Data Fig. 7b,c; 138 ± 30 (mean ± s.e.m.) CEA neurons per animal in Fig. 3b–l). We initially trained mice to consume water at an equal rate from two port locations (Extended Data Fig. 8a and see Methods for details). On the conditioning day, we replaced one port with a novel flavour, whereas water remained in the other as an internal control. Immediately after the consumption period ended, mice were transferred to a distinct second context in which they would experience postingestive malaise. This step was performed to ensure that any neural correlates of flavour consumption that we might subsequently observe were not due to features of the original context in which consumption occurred. After a 30-min delay period in this second context, we induced postingestive malaise using one of the following methods across three groups of mice: (1) optogenetic stimulation of CGRP neuron cell bodies (Fig. 3b–l); (2) optogenetic stimulation of CGRP^CEA^ projections (Extended Data Fig. 9a–e); or (3) injection of LiCl (Fig. 3m–r). Similar results were observed across all three groups of mice; therefore we begin by describing data from the first group.

During consumption, 34% of CEA neurons were significantly activated by the novel flavour compared with only 11% for water (Fig. 3d and Extended Data Fig. 8b) and, in a separate experiment described below, 17% for a familiar flavour. These observations are consistent with our FOS imaging data at the consumption time point (Fig. 1h,i). Almost all CEA neurons, including novel-flavour-coding neurons, were significantly less active after consumption ended (Fig. 3d,e), which suggests that persistent activation (hypothesis 1 in Fig. 3a) is not the mechanism that the amygdala uses to associate flavours with delayed malaise signals.

We next investigated how delayed CGRP neuron stimulation affects CEA neuron activity. CGRP neuron stimulation potently and selectively reactivated novel-flavour-coding CEA neurons, with only limited effects on water-coding and nonselective neurons (Fig. 3d,e; consistent only with hypothesis 2 in Fig. 3a). This reactivation was precisely time locked to individual bouts of CGRP neuron stimulation (Fig. 3f), which suggests that it is directly driven by the release of glutamate from CGRP neuron inputs^34^ (Fig. 2c and Extended Data Fig. 5c) rather than by a slow change in affective or physiological internal state. Similar to CGRP neuron cell-body stimulation, delayed CGRP^CEA^ projection stimulation strongly reactivated novel-flavour-coding CEA neurons (Extended Data Fig. 9a–d).

Reactivation of novel-flavour-coding neurons was similarly present in a separate group of mice that experienced delayed LiCl-induced malaise rather than CGRP neuron stimulation (Fig. 3m,n). Moreover, genetic ablation of CGRP neurons abolished the preferential reactivation of novel-flavour-coding neurons by delayed malaise (Fig. 3p,q) and impaired learning (Extended Data Fig. 9f,g). This finding indicates that the effects of postingestive malaise on CFA—and on CEA dynamics—are mediated by CGRP neurons.

Together, these observations suggest that CEA neurons that encode a recently consumed flavour are selectively reactivated by delayed malaise signals through CGRP neuron inputs, thereby providing a potential mechanism for temporal credit assignment during CFA learning (hypothesis 2 in Fig. 3a).

## Malaise drives population-level flavour reactivations

Population-level analyses corroborated the conclusion that CGRP neuron activity after consumption preferentially reactivates the neural representation of the recently consumed flavour. First, we trained a multinomial logistic regression decoder using population activity during the consumption period to discriminate novel flavour or water consumption from baseline activity (Fig. 3g and Extended Data Fig. 8c,d). Cross-validated decoding accuracy was nearly perfect (Extended Data Fig. 8e). We then evaluated this decoder using population activity during the delay and CGRP neuron-stimulation periods and investigated the probability of decoding the novel flavour or water representation on a moment-by-moment basis (decoder output, *P*(novel flavour|population activity)). This decoding analysis showed that individual bouts of CGRP neuron stimulation reliably reactivated population-level flavour representations (Fig. 3g–i). By contrast, water representations were rarely reactivated by CGRP neuron stimulation (Fig. 3g–i). Consistent with the results from CGRP neuron cell-body stimulation, LiCl-induced malaise also strongly reactivated population-level flavour representations in the CEA (Fig. 3o) and this required functional CGRP neurons (Fig. 3r).

We next compared population-activity trajectories during consumption and during CGRP neuron stimulation by performing principal component analysis (PCA) on the pooled (across mice) trial-averaged population activity during novel-flavour and water consumption. Neural activity during consumption was low-dimensional, with the first two principal components (PCs) explaining >70% of variance in trial-averaged population activity (Fig. 3j). Plotting neural trajectories during novel-flavour and water consumption on the PC1–PC2 axis revealed that PC2 perfectly discriminated these two flavours (Fig. 3k). Using these PCA loadings to project population activity during CGRP neuron stimulation onto the same PC1–PC2 axis showed that this experience closely mirrored the neural trajectory of novel-flavour consumption (Fig. 3k). This strong effect was also apparent in the population activity of individual mice (Fig. 3l) and in the population activity of mice that received delayed CGRP^CEA^ projection stimulation (Extended Data Fig. 9e). Thus, population-level analyses confirmed that CGRP neuron activity specifically reactivates flavour representations in the CEA during delayed postingestive malaise (hypothesis 2 in Fig. 3a).

## Malaise strengthens flavour representations

**Fig. 4 |:**
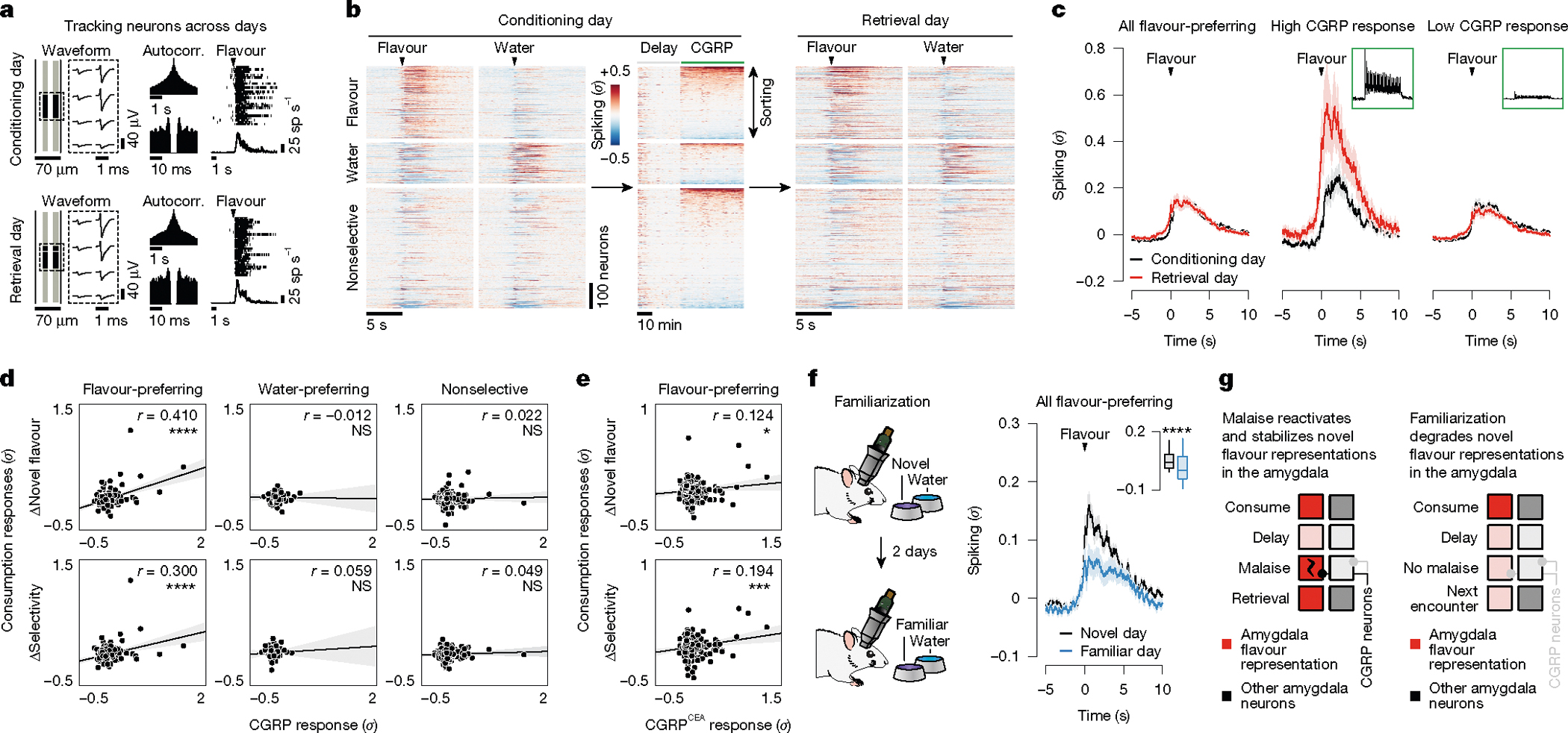
Postingestive CGRP neuron activity induces plasticity to stabilize flavour representations in the amygdala upon memory retrieval. **a**, Spike waveforms, autocorrelograms (Autocorr.) and flavour response rasters for an example neuron tracked across conditioning and retrieval days. **b**, Average spiking of all tracked neurons during the consumption, delay and CGRP neuron-stimulation periods on the conditioning day and during consumption on the retrieval day (*n* = 939 neurons from 8 mice). **c**, Left, average spiking of the novel-flavour-preferring population (*n* = 265 neurons) during flavour consumption on conditioning and retrieval days. Middle and right, average spiking of novel-flavour-preferring neurons with the highest 10% CGRP response magnitudes and of the remaining novel-flavour-preferring neurons. **d**, Correlation between the change (retrieval – conditioning) in flavour response or selectivity for each neuron during consumption to its average response during the CGRP neuron-stimulation period. The novel-flavour-preferring (*n* = 265 neurons), water-preferring (*n* = 123 neurons) and nonselective (*n* = 551 neurons) populations are shown separately. **e**, Analogous to **d**, but for mice with CGRP^CEA^ projection stimulation (*n* = 286 novel-flavour-preferring neurons from 8 mice). **f**, Left, schematic of the flavour-familiarization experiment. Right, average spiking of the initially flavour-preferring population (*n* = 201 neurons from 7 mice; classified on novel-flavour day) during flavour consumption on the novel day and the familiar day. **g**, Illustration of the neural mechanism for learning from delayed postingestive feedback using malaise-driven reactivation and stabilization of flavour representations in the amygdala. Shaded areas in **c** and **f** represent the mean ± s.e.m. and in **d** and **e** represent the linear fit estimate ±95% confidence intervals. Inset box plot shows the 10th, 25th, 50th, 75th and 90th percentiles. *P ≤ 0.05, ***P ≤ 0.001, ****P ≤ 0.0001. See Supplementary Table 2 for details of statistical tests and for exact *P* values.

Tracking neural activity across flavour consumption and malaise revealed that malaise signals preferentially reactivate flavour representations in the amygdala (Fig. 3), thereby providing a potential mechanism for the brain to link flavours experienced during a meal with delayed postingestive feedback. If this mechanism contributes to learning, then postingestive CGRP neuron activity would be expected to trigger functional plasticity in flavour representations in the amygdala that could underlie the CFA memory. To test this hypothesis, we examined whether postingestive reactivation of novel-flavour-coding CEA neurons is predictive of stronger flavour responses when the CFA memory is retrieved. To accomplish this task, we took advantage of the high stability of our chronic recordings to track the same CEA neurons across days and analysed their responses to flavour consumption before (conditioning day) and after (retrieval day) pairing with CGRP neuron stimulation (Fig. 4a,b and see Methods for details).

**Extended Data Fig. 10 |:**
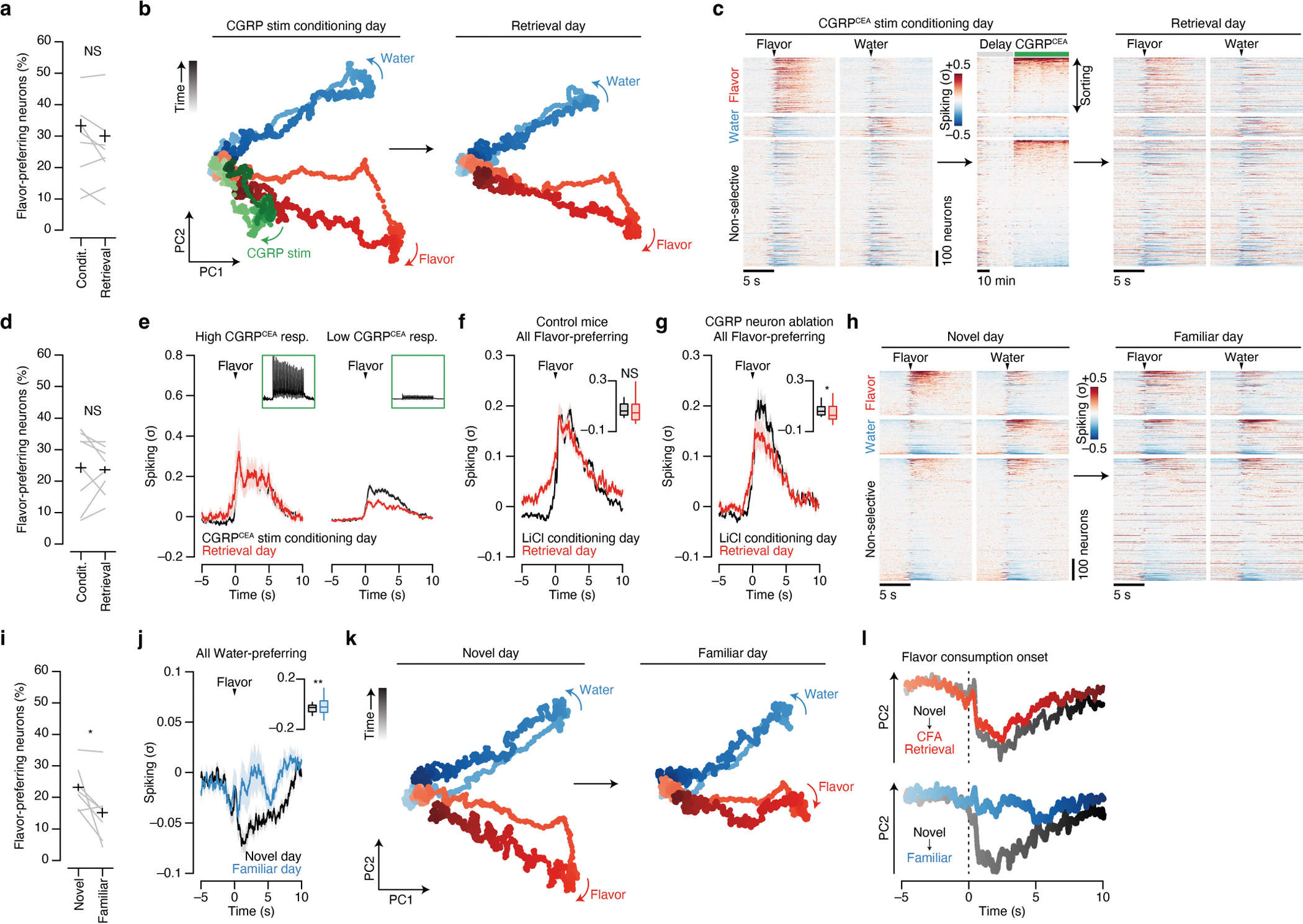
Postingestive CGRP neuron activity is necessary and sufficient to stabilize flavour representations in the amygdala upon memory retrieval. Panels **a**,**b** relate to Fig. 4b–d. **a**, Proportion of flavour-preferring neurons classified separately on conditioning or retrieval day (*n* = 8 mice). **b**, Population trajectories for flavour consumption, water consumption, and CGRP neuron stimulation. Panels **c**–**e** relate to Fig. 4e. **c**, Average spiking of all individual neurons for the CGRP^CEA^ projection stimulation experiment (*n* = 1,042 neurons from 8 mice). **d**, Analogous to **a**, but for mice with CGRP^CEA^ projection stimulation (*n* = 8 mice). **e**, Average spiking of the novel flavour-preferring neurons with the highest 10% CGRP^CEA^ response magnitudes and of the remaining novel flavour-preferring neurons. Panels **f**,**g** show that LiCl-induced malaise stabilizes the flavour representation upon retrieval, and that this is impaired by CGRP neuron ablation. **f**, For control mice, average spiking of the novel flavour-preferring population (*n* = 279 neurons from 4 mice). **g**, Analogous to **f**, but for mice with CGRP neuron ablation (*n* = 109 neurons from 4 mice). Panels **h**–**k** relate to Fig. 4f. **h**, Average spiking of all individual neurons (*n* = 924 neurons from 7 mice). **i**, Proportion of flavour-preferring neurons classified separately on novel or familiar day (*n* = 7 mice). **j**, Average spiking of the initially water-preferring population (*n* = 160 neurons from 7 mice; classified on novel day) during flavour consumption. **k**, Population trajectories for flavour and water consumption. **l**, Time-courses along the PC2 axis during consumption following CGRP neuron stimulation conditioning (from **b**) and familiarization (from **k**). Error bars and shaded areas represent mean ± s.e.m. Inset box plots show the 10^th^, 25^th^, 50^th^, 75^th^, and 90^th^ percentiles. NS, not significant, *P ≤ 0.05, **P ≤ 0.01. See Supplementary Table 2 for details of statistical tests and for exact *P* values.

The trial-averaged response of novel-flavour-coding CEA neurons (classified on the conditioning day) was largely stable across days (Fig. 4c). However, sorting these neurons on the basis of the magnitude of their response to CGRP neuron stimulation revealed a notable effect. Specifically, novel-flavour-coding CEA neurons with the greatest CGRP neuron input responded more strongly to the flavour during memory retrieval, whereas the responses of novel-flavour-coding neurons with weak or no CGRP neuron input remained relatively unchanged (Fig. 4c,d). By contrast, we did not observe a similar correlation for water-coding or nonselective CEA neurons (Fig. 4d), which further suggests that postingestive malaise does not recruit additional neurons into the initial flavour representation (as in hypothesis 3 in Fig. 3a). Together, these observations indicate that CGRP neurons induce functional plasticity that stabilizes the response of the amygdala to the conditioned flavour after learning. Consistent with this conclusion, the population-level flavour representation, as visualized using PCA, was highly stable across conditioning and retrieval days (Extended Data Fig. 10b,l).

CGRP^CEA^ projection stimulation (Fig. 4e and Extended Data Fig. 10c–e) and LiCl-induced malaise (Extended Data Fig. 10f) had similar stabilizing effects on novel-flavour-coding neuron responses during memory retrieval compared to CGRP neuron cell-body stimulation. By contrast, the responses of novel-flavour-coding neurons significantly decreased during memory retrieval in mice lacking CGRP neurons (Extended Data Fig. 10g). Thus, CGRP neuron activity is necessary and sufficient for both the reactivation and stabilization of flavour representations in the amygdala by delayed malaise signals.

For comparison, we next asked how amygdala activity evolves after familiarization (that is, experience with a flavour without any aversive postingestive consequences, as in the ‘Familiar’ condition in Figs. 1 and 2). Consistent with our initial FOS imaging data comparing activation patterns between novel and familiar flavours (Fig. 1h,i), tracking CEA neuron activity before and after familiarization revealed that the proportion of flavour-coding neurons significantly decreased after familiarization (Extended Data Fig. 10h,i). Similarly, the trial-averaged response of individual flavour-coding neurons (classified on the novel-flavour day) significantly decreased after familiarization (Fig. 4f). This result was in contrast to the stability we observed following conditioning with CGRP neuron stimulation (Fig. 4c, left) and LiCl-induced malaise (Extended Data Fig. 10f). Furthermore, initially water-preferring neurons increased their response to the flavour after it became familiar (Extended Data Fig. 10j). Together, these observations suggest that familiarization degrades flavour representations in the amygdala such that the representation of a flavour moves closer to the representation of pure water. Consistent with this conclusion, the modulation of population-level activity along the PC2 dimension that discriminates flavour from water was almost completely abolished following familiarization (Extended Data Fig. 10k,l).

Thus, CGRP neurons convey malaise signals that preferentially reactivate flavour representations in the amygdala, which may enable the brain to bridge the delay between a meal and postingestive feedback during CFA learning. Moreover, these postingestive signals induce plasticity to stabilize or strengthen flavour representations after conditioning, whereas flavour representations rapidly degrade in the absence of malaise signals as flavours become familiar and safe (Fig. 4g).

## Novel flavours trigger PKA activity

**Fig. 5 |:**
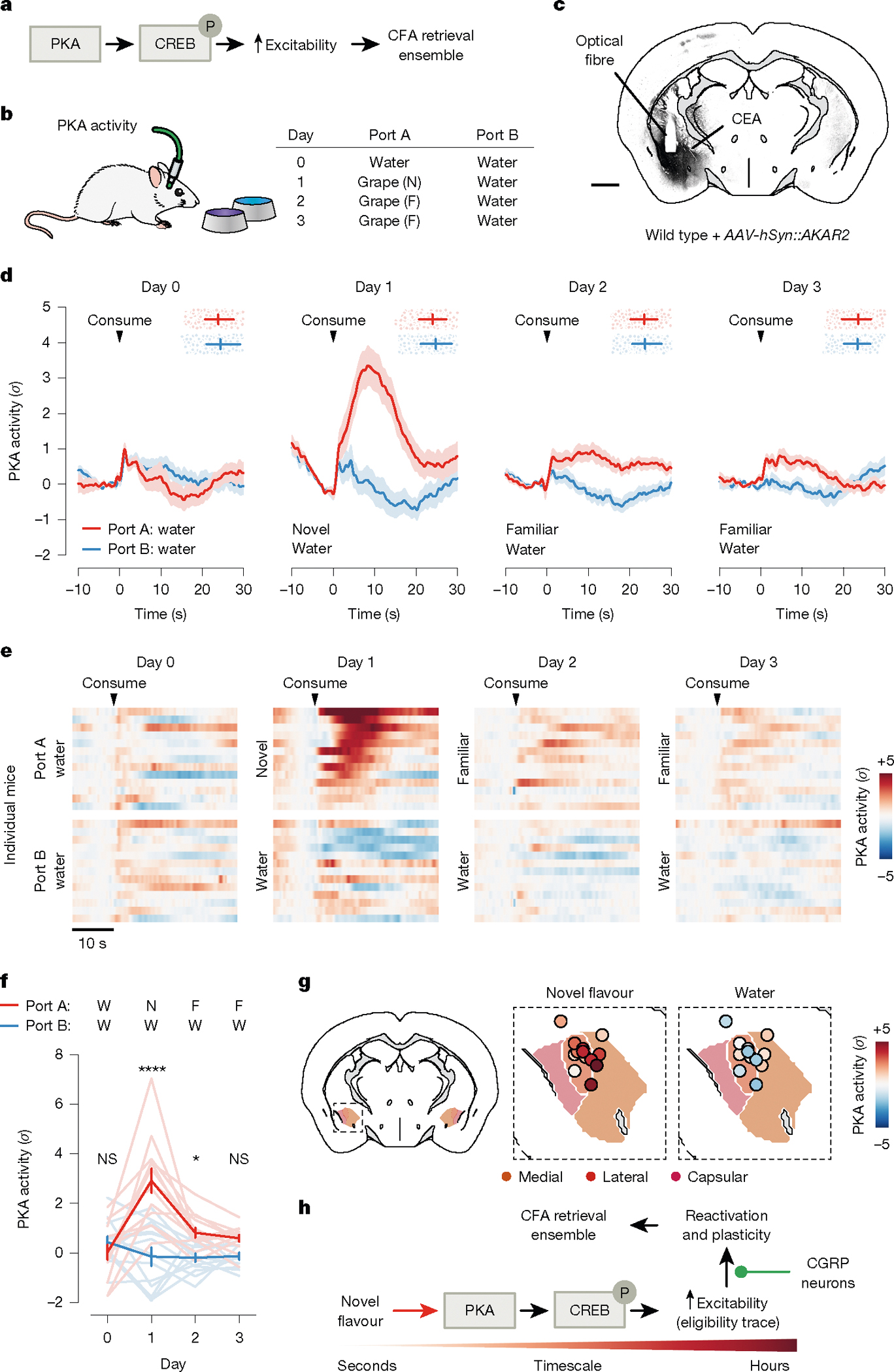
Novel-flavour consumption triggers PKA activity in the amygdala, which provides a potential biochemical eligibility trace for reactivation by postingestive CGRP neuron activity. **a**, Simplified schematic of the biochemical pathway that has been proposed to allocate a subset of amygdala neurons to the CFA memory-retrieval ensemble (or ‘memory engram’)^40–46^. **b**, Schematic of the strategy for recording PKA activity in the CEA across flavour-familiarization using the AKAR2 sensor^47^. F, familiar; N, novel. **c**, Example image of AKAR2 expression in the CEA. Scale bar, 1 mm. **d**, PKA activity in the CEA in response to consumption of a novel or familiar flavour (port A) and to water (port B) across four consecutive days (*n* = 13 mice). Points and error bars at the top of each plot indicate the timing of the next reward consumption. **e**, PKA activity for individual mice in response to a novel or familiar flavour and to water consumption. Mice were sorted by novel-flavour response (day 1). **f**, Summary of PKA activity in response to a novel or familiar flavour and to water consumption (*n* = 13 mice). **g**, PKA activity in response to novel flavour (left) and water (right) consumption on day 1 for individual mice aligned to the Allen CCF (*n* = 13 mice). **h**, Schematic of a putative hypothesis linking biochemistry with neural activity during CFA, with novelty-dependent increases in PKA in novel-flavour-coding amygdala neurons leading to increased reactivation of these cells by delayed CGRP neuron inputs and recruitment to the CFA memory-retrieval ensemble. Error bars in **d** represent the 25th, 50th and 75th percentiles and in **f** represent the mean ± s.e.m. Shaded areas represent the mean ± s.e.m. *P ≤ 0.05, ****P ≤ 0.0001. See Supplementary Table 2 for details of statistical tests and for exact *P* values.

So far, we have focused on the role of neural activity in supporting CFA. Previous work in the field has taken a complementary approach to examine the role of biochemical signals^38,39^. For example, many studies have shown an important role in the amygdala for cAMP response element-binding protein (CREB)^40–42^, a transcription factor that regulates neural excitability, and protein kinase A (PKA)^43,44^, which phosphorylates and activates CREB. CREB activity levels at the time of conditioning are thought to bias neurons towards allocation to the CFA ‘memory engram’^45,46^: the ensemble of cells that are activated during retrieval of the CFA memory (Fig. 5a).

We therefore investigated how the PKA→CREB pathway might relate to the malaise-driven reactivation and stabilization of flavour representations that we describe here. One possibility is that this biochemical pathway could be preferentially triggered in the amygdala by novel-flavour consumption, which may in turn contribute to increased excitability or responsiveness of novel-flavour-coding neurons to CGRP neuron inputs during malaise.

To test the first part of this hypothesis—that the PKA→CREB pathway is preferentially activated by novel flavours—we recorded in vivo PKA activity in the CEA through fibre photometry measurements of the AKAR2 sensor^47^ (Fig. 5b,c). These recordings showed that novel flavours drive a strong increase in PKA activity in the CEA, whereas familiar flavours have little impact on PKA activity (Fig. 5d–g). This increase in PKA activity (tens of seconds) was substantially longer in duration than the increase in spiking for each bout of consumption (Fig. 3d). Downstream effects on CREB, gene expression and neural excitability are presumably far longer-lasting. Thus, novelty-dependent gating of PKA could serve as a biochemical eligibility trace that increases the responsiveness of novel-flavour-coding neurons to delayed malaise signals, thereby permitting the selective reactivation and plasticity of novel-flavour representations in the amygdala (Fig. 5h).

## Discussion

The major reason that learning is challenging is because of delays between a stimulus or action and its outcome. This raises the question of how the brain assigns credit to the correct previous event. Most work on credit assignment has been limited to examining learning in the case of relatively short delays (on the order of seconds)^48–51^. Postingestive learning paradigms, such as CFA, provide an opportunity to study how the brain assigns credit across much longer delays.

Here we described a neural mechanism that may contribute to solving the credit-assignment problem inherent to CFA. That is, postingestive malaise signals selectively reactivate the neural representations of flavours experienced during a recent meal, and this reactivation serves to stabilize or strengthen the flavour representation upon memory retrieval.

Although previous work, mostly in the hippocampus and cortex, has suggested a role for neural reactivations in learning and memory^52,53^, our study advances this idea in multiple important ways. First, we applied this concept to a new paradigm: postingestive learning and CFA. Second, we discovered a role for the outcome signal (unconditioned stimulus) in directly triggering reactivations and demonstrated that a cell-type-specific malaise pathway mediates this effect. Third, we discovered a relationship between outcome-driven flavour reactivations and strengthened flavour representations during memory retrieval.

Our entry point into this problem was the fact that novel flavours more easily support postingestive learning than familiar flavours^14,15^. By comparing brain-wide neural-activation patterns in animals that consumed the same flavour when it was novel versus familiar, we identified an amygdala network that was unique in preferentially responding to novel flavours across every stage of learning. Although we focused here on how novel flavours become associated with aversive postingestive feedback in this amygdala network, it is possible that these same ideas generalize to the processes of familiarization or postingestive nutrient learning. Specifically, a parallel and mechanistically similar process may be at work when learning that a food is safe or nutritious. In that case, safety (or reward^11–13^) signals may reactivate recently consumed flavour representations, in contrast to the aversive CGRP neuron-mediated reactivations we report here. This may enable the weakening of flavour representations in the amygdala (Fig. 4f) and/or the strengthening of flavour representations in the LS and other limbic regions (Extended Data Fig. 1b).

The degree of specificity of malaise-driven reactivations for a recently consumed novel flavour (versus other flavours), and what mechanisms may contribute to such specificity, remain open questions. One possibility is that preferential activation of the PKA→CREB pathway by consumption of a particular novel flavour (Fig. 5) may provide a biochemical eligibility trace to facilitate the selective reactivation and strengthening of the neural representation of that flavour (versus other flavours) by delayed outcome signals.

Previous recording experiments during CFA have concentrated on the role of the gustatory insular cortex^54–59^. A consistent finding is that CFA amplifies the cortical representation of the conditioned tastant and shifts it to be more similar to innately aversive tastants. Recent work has further shown that homeostatic synaptic plasticity in the insula contributes to a transition, over the course of hours or days, from the initial formation of a more generalized taste aversion to a tastant-specific CFA memory^60^. How the malaise-driven reactivation of flavour representations in the amygdala we report here relates to such mechanisms in the insula requires further study. One possibility is that the reactivation of flavour representations by delayed malaise signals contributes to the formation of an initial memory, which is then further refined through homeostatic mechanisms in the insula and its reciprocal connections with the amygdala.

Overall, our results reveal how dedicated novelty-detection circuitry and built-in priors (preferential reactivation of recent flavour representations by postingestive malaise) work together to enable the brain to correctly link stimuli and outcomes despite long delays.

## Online content

Any methods, additional references, Nature Portfolio reporting summaries, source data, extended data, supplementary information, acknowledgements, peer review information; details of author contributions and competing interests; and statements of data and code availability are available at https://doi.org/10.1038/s41586-025-08828-z.

## Methods

### Animals and surgery

All experimental procedures were approved by the Princeton University Institutional Animal Care and Use Committee following the NIH Guide for the Care and Use of Laboratory Animals. Wild-type mice (JAX 000664) and *Calca*^*cre*^ mice^34^ (JAX 033168) were obtained from the Jackson Laboratory. Adult mice (>8 weeks old) of both sexes were used for all experiments. Mice were housed under a 12-h light–dark cycle, and experiments were conducted during the dark cycle. Ambient temperature was maintained at 21–26 °C and humidity at 30–70%. Stereotaxic surgeries were performed under isoflurane anaesthesia (3–4% for induction, 0.75–1.5% for maintenance). Mice received pre-operative antibiotics (5 mg kg^−1^ Baytril subcutaneous (s.c.)) and pre-operative and post-operative analgesia (10 mg kg^−1^ Ketofen s.c.; 3 daily injections). Post-operative health (evidence of pain, incision healing, activity and posture) was monitored for at least 5 days. For all CFA experiments, mice were water-restricted and maintained at >80% body weight for the duration of the experiment.

### Viral injections

For CGRP neuron cell-body stimulation experiments (Figs. 2–4), we bilaterally injected 400 nl of AAV5-EF1a-DIO-hChR2(H134R)-eYFP (titre, 1.2 × 10^13^ genome copies (GC) per ml; manufacturer, Princeton Neuroscience Institute (PNI) Viral Core Facility)^61,62^ at − 5.00 mm anterior–posterior (AP), ±1.40 mm medial–lateral (ML) and −3.50 mm dorsal–ventral (DV) into *Calca*^*cre*^ mice. We used these stereotaxic coordinates to target the PB in all subsequent experiments. For CGRP neuron fibre photometry experiments (Fig. 2b), we unilaterally injected 400 nl of AAV9-hSyn-FLEX-GCaMP6s (titre, 1.0 × 10^13^ GC per ml; manufacturer, PNI Viral Core Facility)^63^ into the PB of *Calca*^*cre*^ mice. For CGRP^CEA^ projection stimulation experiments (Figs. 2 and 4 and Extended Data Fig. 9), we bilaterally injected 350 nl of AAV5-EF1a-DIO-hChR2(H134R)-eYFP (titre, 1.2 × 13 GC per ml; manufacturer, PNI Viral Core Facility; RNAscope FISH experiment)^61,62^, AAV5-EF1a-DIO-ChRmine-mScarlet (titre, 9.0 × 10^12^ GC per ml; manufacturer, PNI Viral Core Facility; all other experiments)^64^ or AAV5-EF1a-DIO-eYFP (titre, 1.5 × 10^13^ GC per ml; manufacturer, PNI Viral Core Facility) into the PB of *Calca*^*cre*^ mice. For CGRP^CEA^ projection inhibition experiments (Fig. 2f), we bilaterally injected 350 nl of AAV5-hSyn-SIO-eOPN3-mScarlet (titre, 9.0 × 10^12^ GC per ml; manufacturer, Addgene)^65^ or AAV5-EF1a-DIO-eYFP (titre, 1.5 × 10^13^ GC per ml; manufacturer, PNI Viral Core Facility) into the PB of *Calca*^*cre*^ mice. For CGRP neuron ablation experiments (Fig. 3p–r and Extended Data Fig. 9g,h), we bilaterally injected 350 nl of AAV5-EF1a-FLEX-taCasp3-TEVp (titre, 1.6 × 10^13^ GC per ml; manufacturer, Addgene)^66^ into the PB of *Calca*^*cre*^ mice. For control LiCl conditioning and Neuropixels implantation experiments (Fig. 3n,o), we bilaterally injected 350 nl of AAV5-Camk2a-eYFP (titre, 7.5 × 10^11^ GC per ml; manufacturer, University of North Carolina (UNC) Vector Core) into the PB of wild-type mice. For CEA PKA recording experiments (Fig. 5), we unilaterally injected 300 nl of AAV5-hSyn-ExRai-AKAR2 (titre, 2.4 × 10^13^ GC per ml; manufacturer, PNI Viral Core Facility)^47^ at −1.15 mm AP, −2.65 mm ML and −4.85 mm DV into wild-type mice. For LS activation experiments (Extended Data Fig. 2), we bilaterally injected AAV5-hSyn-hM3D(G_q_)-mCherry (titre, 3.8 × 10^12^ GC per ml; manufacturer, Addgene)^67^ or AAV5-Camk2a-eYFP (titre, 7.5 × 10^11^ GC per ml; manufacturer, UNC Vector Core) at one (500 nl at +0.55 mm AP, ±0.35 mm ML and −4.00 mm DV) or two (150 nl each at +0.85 mm or +0.25 mm AP, ±0.60 mm ML and −3.75 mm DV) coordinates into wild-type mice. Virus was infused at 100 nl min^−1^. Coordinates are given relative to bregma. We allowed 3 weeks for AKAR2 and GCaMP expression, at least 4 weeks for ChR2 and hM3D expression, 5 weeks for CGRP neuron ablation by taCasp3-TEVp and 8 weeks for CGRP^CEA^ terminal expression of ChRmine, ChR2 and eOPN3.

### Optical fibre implantations

Optical fibres encased in stainless-steel ferrules were implanted into the brain for optogenetic and fibre photometry experiments. For bilateral optogenetic stimulation of CGRP neurons (Fig. 2), we implanted 300 μm core diameter, 0.39 NA fibres (Thorlabs, FT300EMT) above the PB at a 10° angle, with the fibre tips terminating 300–400 μm above the viral injection coordinate. For unilateral stimulation of CGRP neurons (Figs. 3 and 4), we implanted a 300 μm core diameter, 0.39 NA fibre above the left PB at a 25–30° angle, with the fibre tip terminating 300–400 μm above the viral injection coordinate. For bilateral optogenetic manipulation of CGRP^CEA^ projections (Fig. 2), we implanted 300 μm core diameter, 0.39 NA fibres above the CEA, with the fibre tips terminating at −1.15 mm AP, ±2.85 mm ML and −4.25 mm DV. For unilateral optogenetic stimulation of CGRP^CEA^ projections (Fig. 4 and Extended Data Figs. 9 and 10), we implanted a 300 μm core diameter, 0.37 NA fibre (Doric, MFC_300/360–0.37_10mm_MF2.5_FLT) above the left CEA at a +55° angle, with the fibre tip terminating at −.20 mm AP, +2.25 mm ML and −3.55 mm DV. For fibre photometry recording of CGRP neurons (Fig. 2b), we implanted a 400 μm core diameter, 0.48 NA fibre (Doric, MFC_400/430–0.48_5.0mm_MF2.5_FLT) above the left PB at a −10° to −30° angle, with the fibre tip terminating approximately at the viral injection coordinate. For fibre photometry recording of CEA PKA activity (Fig. 5), we implanted a 400 μm core diameter, 0.48 NA fibre (Doric, MFC_400/430–0.48_6.0mm_MF2.5_FLT) above the left CEA, with the fibre tip terminating approximately at the viral injection coordinate. Optical fibres were affixed to the skull with Metabond (Parkell, S380), which was then covered in acrylic dental cement.

### Chronic Neuropixels assembly

We used four-shank Neuropixels 2.0 probes^37^ (test-phase; Imec), as they were miniaturized to make them more suitable for chronic implantation in mice. To avoid directly cementing the probes to the skull (that is, so that the probes could be reused), we designed a chronic implant assembly (Extended Data Fig. 7a) based on the design for Neuropixels 1.0 probes previously validated in rats^68^. Similar to that design, the assembly was printed on Formlabs SLA 3D printers and consisted of four discrete parts: (1) a dovetail adapter permanently glued to the probe base; (2) an internal holder that mated with the dovetail adapter and facilitated stereotaxic manipulation of the probe; and (3–4) an external chassis, printed in two separated parts, that encased and protected the entire assembly. The external chassis and internal holder were attached using screws that could be removed at the end of the experiment to enable explantation and reuse. The external chassis of the final implant assembly was coated with Metabond before implantation. After explantation, probes were cleaned with consecutive overnight washes in enzyme-active detergent (Alconox Tergazyme) and silicone cleaning solvent (Dowsil, DS-2025) before reuse. The dimensions of the Neuropixels 2.0 implant assembly were significantly smaller than the Neuropixels 1.0 implant assembly^68^, primarily because of the smaller size of the probe and headstage. The maximum dimensions were 24.7 mm (height), 12.2 mm (width) and 11.2 mm (depth), with a weight of 1.5 g (not including the headstage). Space was made for the headstage to be permanently housed in the implant, as opposed to the previous design in which the headstage was connected only during recording and was secured to a tether attached to the animal. This made connecting the animal to the assembly for a recording significantly easier and obviated the need for a bulky tether that limits the movements of the animal. This change was made possible owing to improvements in Neuropixels cable design, which required fewer cables per probe and less reinforcement of the cables during free movement. Design files and instructions for printing and assembling the chronic Neuropixels 2.0 implant are available from GitHub (https://github.com/agbondy/neuropixels_2.0_implant_assembly).

### Chronic Neuropixels surgery

Surgery was performed 3–4 weeks after AAV injection to allow time for viral expression and behavioural training. First, three craniotomies were drilled: one small craniotomy (500 μm diameter) above the left PB (approached at a −10° to −30° angle) or the left CEA (approached at a +55° angle) for the optical fibre, another small craniotomy above the cerebellum for the ground wire, and one large craniotomy (1 × 2 mm) above the left CEA for the Neuropixels probe. Next, a single optical fibre was placed above the left PB or left CEA as described above. At this point, the optical fibre was affixed to the skull with Metabond and the exposed skull was covered with Metabond. Next, a prefabricated chronic Neuropixels assembly was lowered at 2.5 μm s^−1^ into the CEA using an ultraprecise micromanipulator (Sensapex μMp). The probe shanks were aligned with the AP axis of the skull, with the most anterior shank tip terminating at −0.95 mm AP, −2.95 mm ML and −6.50 mm DV. Once the probe was fully lowered, the stainless-steel ground wire was inserted 1–2 mm into the cerebellum and affixed with Metabond. The CEA craniotomy and probe shanks were then covered with medical-grade petroleum jelly, and Dentin (Parkell, S301) was used to affix the chronic Neuropixels assembly to the Metabond on the skull. The optical fibres and CEA Neuropixels probe were both placed in the left hemisphere because CGRP neuron projections are primarily ipsilateral^24^.

### One-reward CFA paradigm

As shown in Figs. 1 and 2, we used a one-reward CFA paradigm that used either a novel or familiar flavour. Experiments were performed in operant boxes (Med Associates) using MedPC software (https://med-associates.com/product/med-pc; v.IV). Operant boxes were situated in sound-attenuating chambers and equipped with a single nosepoke port and light. The nosepoke port contained a reward-delivery tube that was calibrated to deliver 20 μl of reward through a solenoid valve (Lee Technologies, LHDA2433315H). Every behavioural session (training and conditioning) had the following basic structure. First, the mouse was allowed to acclimate to the chamber for 5 min. Then, the consumption period began and the port light turned on to indicate that rewards were available. During this period, each nosepoke, detected by an infrared beam break with a 1 s time-out period, triggered the delivery of a single reward, and the period ended when 1.2 ml of reward was consumed or 10 min had passed. Then, the delay period began and lasted until 30 min after the beginning of the consumption period. During training sessions, mice were returned to the home cage after the end of the delay period.

Mice assigned to the novel-flavour condition first received four training days as described above with water as the reward and no LiCl or CGRP neuron stimulation. On the conditioning day, sweetened grape Kool-Aid (0.06% grape and 0.3% saccharin sodium salt; Sigma, S1002) was the reward. Mice assigned the familiar-flavour condition had sweetened grape Kool-Aid as the reward for all four training days as well as on the conditioning day.

On the LiCl conditioning day (Fig. 1), mice received an i.p. injection of LiCl (125 mg kg^−1^; Fisher Scientific, L121) or normal saline after the 30-min delay after the end of the consumption period. For the CGRP neuron cell-body stimulation (Fig. 2d) and CGRP^CEA^ projection stimulation (Fig. 2e) experiments, mice then received 45 min of intermittent stimulation beginning after the 30 min of delay. Blue light was generated using a 447 nm laser for ChR2 experiments. Green light was generated using a 532 nm laser for ChRmine experiments. The light was split through a rotary joint and delivered to the animal using 200 μm diameter core patch cables. Light power was calibrated to approximately 10 mW at the patch cable tip for ChR2 experiments and 3 mW for ChRmine experiments. During the experiment, the laser was controlled with a Pulse Pal signal generator (Sanworks, 1102) programmed to deliver 5 ms laser pulses at 10 Hz. For the duration of the stimulation period, the laser was pulsed for 1.5–15 s intervals (randomly chosen from a uniform distribution with 1.5 s step size) and then off for 1–10 s intervals (randomly chosen from a uniform distribution with 1 s step size). For the eOPN3 experiment (Fig. 2f), photoinhibition began 1 min before the LiCl injection and then continued for 90 min (532 nm laser, 10 mW power, 500 ms laser pulses at 0.4 Hz). Mice were then returned to the home cage. For the LS activation experiments (Extended Data Fig. 2), mice received an i.p. injection of 3 mg kg^−1^ clozapine *N*-oxide (CNO; Hellobio, 6149) 45 min before the experiment began.

We assessed learning using a two-bottle memory retrieval test. Two bottles were affixed to the side of a mouse cage (Animal Care Systems Optimice) such that the sipper tube openings were located approximately 1 cm apart. One day after conditioning, mice were given 30 min of access with water in both bottles. We calculated a preference for each mouse for this session and then counterbalanced the location of the test bottle for the retrieval test such that the average water day preference for the two bottle locations was as close to 50% as possible for each group. The next day, mice were given 30 min of access with water in one bottle and sweetened grape Kool-Aid in the other bottle. Flavour preference was then calculated using the weight consumed from each bottle during this retrieval test: flavour/(flavour + water).

To initially characterize behaviour in our CFA paradigm (Fig. 1b), retrieval tests were conducted on three consecutive days with the flavour bottle in the same location each day for each mouse. We then fit a GLMM to this dataset using the R package glmmTMB^69^ (https://github.com/glmmTMB/glmmTMB; v.1.17) with a Gaussian link function and the formula:

(1) Preference~Novel*Injection*Day+Sex+(1|Subject)

where Preference is the retrieval test result, Novel (novel, familiar), Injection (LiCl, saline), Day (day 1, day 2, day 3) and Sex (female, male) are fixed-effect categorical variables, (1|Subject) is a random effect for each mouse, the asterisk represents the main effects and interactions, and the tilde means ‘distributed as’. This GLMM showed a strong novel–injection interaction effect (*P* = 2.22 × 10^−6^, coefficient estimate *z* test, *n* = 32 mice) and a weak effect of novel alone (*P* = 0.025), but no effect of sex (*P* = 0.137) or injection (*P* = 0.574) alone or for any other effects. Using the coefficients from this GLMM, we then used the R package marginaleffects^70^ (https://github.com/vincentarelbundock/marginaleffects; version 0.12.0) to calculate the marginal effect of the flavour condition (novel – familiar) on each day independently for each injection group. We used the marginal effect estimates and s.e. values to calculate a *P* value for each injection–day combination with a *z* test, and then corrected for multiple comparisons in each injection group using the Hochberg–Bonferroni step-up procedure^71^.

For subsequent experiments (Fig. 2d–f and Extended Data Fig. 2b), we performed a single retrieval test per animal and tested for significant differences across groups using Wilcoxon rank-sum tests.

### Histology

We visualized mCherry, mScarlet and YFP signals to validate transgene expression in our LS chemogenetics (Extended Data Fig. 2a,b) and CGRP neuron optogenetics (Fig. 2d–f) experiments. Mice were deeply anaesthetized (2 mg kg^−1^ Euthasol i.p.) and then transcardially perfused with PBS followed by 4% paraformaldehyde (PFA) in PBS. Brains were then extracted and post-fixed overnight in 4% PFA at 4 °C and then cryoprotected overnight in 30% sucrose in PBS at 4 °C. Free-floating sections (40 μm) were prepared with a cryostat (Leica Microsystems, CM3050S), mounted with DAPI Fluoromount-G (Southern Biotech, 0100) and imaged with a slide scanner (Hamamatsu, NanoZoomer S60) using NDP Scan software (https://www.hamamatsu.com; v.3.4).

To visualize ExRai–AKAR2 signals (Fig. 5c), we stained for GFP immunoreactivity in the CEA. To validate CGRP neuron ablation following taCasp3-TEVp injection (Fig. 3p and Extended Data Fig. 9f), we stained for CGRP immunoreactivity in the PB. In brief, sections were washed, blocked (3% normal donkey serum (NDS) and 0.3% Triton-X in PBS for 90 min) and then incubated with primary antibody (rabbit anti-GFP, Novus, NB600–308, 1:1,000; mouse anti-CGRP, Abcam, ab81887, 1:250) in blocking buffer overnight at 4 °C. Sections were then washed, incubated with secondary antibody (Alexa Fluor 647 donkey anti-rabbit, Invitrogen, A31573, 1:500; Alexa Fluor 568 donkey anti-mouse, Life Technologies, A10037, 1:500) in blocking buffer for 90 min at room temperature, washed again, mounted with DAPI Fluoromount-G (Southern Biotech, 0100) and imaged with a slide scanner (Hamamatsu, NanoZoomer S60) using NDP Scan software (https://www.hamamatsu.com; v.3.4).

Basic image processing, such as brightness and contrast adjustment, was performed using Fiji^72^ (https://fiji.sc; v.1.52).

### Mouse brain atlas

The reference atlas we used is based on the 25 μm resolution Allen Mouse Brain CCF v.3 (https://atlas.brain-map.org)^29^. For FOS imaging experiments, we considered every brain region in the atlas that met the following criteria: (1) total volume ≥0.1 mm^3^; (2) lowest level of its branch of the ontology tree (cortical layers or zones not included). We made two modifications to the standard atlas for this study.

First, we reassigned brain region identifiers to increase the clarity of our FOS visualizations that incorporate the atlas and to accurately represent the full functional extent of the CEA. We merged several small regional subdivisions together into the larger LG, PVH, PV, MRN, PRN and SPV regions (see Supplementary Table 1 for a list of brain-region abbreviations). We reassigned all cortical layers and zones to their immediate parent regions (for example, ‘Gustatory areas, layers 1–6b’ (111–117) were reassigned to ‘Gustatory areas’ (110)). We merged all unassigned regions (tagged with the ‘-un’ suffix in the Allen CCF) into relevant parent regions (for example, ‘HPF-un’ (563) was reassigned to ‘Hippocampal formation’ (462)). We reassigned the voxels immediately surrounding the CEA that were assigned to the ‘Striatum’ (581) to the ‘Central amygdalar nucleus’ (605), because we found that cells localized to these CEA-adjacent voxels had highly similar FOS and Neuropixels responses compared with cells localized strictly in the CEA. These atlas changes were used throughout the paper (FOS imaging experiments and Neuropixels experiments). Summaries across the entire CEA (for example, Figs. 1i, 2h, 3 and 4) included all atlas voxels assigned to the parent CEA region (605) and to the CEAc (606), CEAl (607) and CEAm (608) subdivisions. The summary across the entire LS (Extended Data Fig. 2d) included all atlas voxels assigned to the parent ‘Lateral septal complex’ region (594) and to the LS subdivision (595).

Second, we made the left and right hemispheres symmetric to facilitate the pooling of data from both hemispheres for our FOS visualizations. To ensure that the hemispheres of the Allen CCF were perfectly symmetric, we replaced the left hemisphere with a mirrored version of the right hemisphere. This atlas change was used only for the FOS experiments.

### Brain-wide FOS time points

All mice used for the FOS experiments (Figs. 1 and 2 and Extended Data Figs. 1–4 and 6) were trained in the one-reward CFA paradigm as described above. For the consumption time point (Fig. 1), mice were euthanized 60 min after the end of the consumption period on the conditioning day (no LiCl injection was given). For the malaise time point (Fig. 1), mice were euthanized 60 min after the LiCl injection on the conditioning day. For the retrieval time point (Fig. 1), mice received the LiCl conditioning described above and then were returned to the operant box 2 days later for another consumption of the paired flavour using the same task structure as described above. Mice were euthanized 60 min after the end of the consumption period of the retrieval session (no LiCl was given during the retrieval session). For the CGRP neuron stimulation time point (Fig. 2), mice were euthanized 60 min after the onset of CGRP neuron stimulation on the conditioning day and stimulation continued for the full 60 min. For the LS activation time point (Extended Data Fig. 2), mice received an i.p. injection of 3 mg kg^−1^ CNO 45 min before consumption and were then euthanized 60 min after the LiCl injection on the conditioning day.

Mice were deeply anaesthetized (2 mg kg^−1^ Euthasol i.p.) and then transcardially perfused with ice-cold PBS and heparin (20 U ml^−1^; Sigma, H3149) followed by ice-cold 4% PFA in PBS. Brains were then extracted and post-fixed overnight in 4% PFA at 4 °C.

### Tissue clearing and immunolabelling

Brain samples were cleared and immunolabelled using an iDISCO+ protocol as previously described^28,73^. All incubations were performed at room temperature unless otherwise noted.

#### Clearing.

Brain samples were serially dehydrated in increasing concentrations of methanol (Carolina Biological Supply, 874195; 20%, 40%, 60%, 80% and 100% in doubly distilled water (ddH_2_O); 45 min–1 h each), bleached in 5% hydrogen peroxide (Sigma, H1009) in methanol overnight and then serially rehydrated in decreasing concentrations of methanol (100%, 80%, 60%, 40% and 20% in ddH_2_O; 45 min–1 h each).

#### Immunolabelling.

Brain samples were washed in 0.2% Triton X-100 (Sigma, T8787) in PBS, followed by 20% DMSO (Fisher Scientific, D128), 0.3 M glycine (Sigma, 410225) and 0.2% Triton X-100 in PBS at 37 °C for 2 days. Brains were then washed in 10% DMSO and 6% NDS (EMD Millipore S30) and 0.2% Triton X-100 in PBS at 37 °C for 2–3 days to block nonspecific antibody binding. Brains were then washed twice for 1 h at 37 °C in 0.2% Tween-20 (Sigma P9416) and 10 mg ml^−1^ heparin in PBS (PTwH solution) followed by incubation with primary antibody solution (rabbit anti-FOS, 1:1,000; Synaptic Systems, 226008) in 5% DMSO, 3% NDS and PTwH at 37 °C for 7 days. Brains were then washed in PTwH 6 times for increasing durations (10 min, 15 min, 30 min, 1 h, 2 h and overnight) followed by incubation with secondary antibody solution (Alexa Fluor 647 donkey anti-rabbit, 1:200; Abcam, ab150075) in 3% NDS and PTwH at 37 °C for 7 days. Brains were then washed in PTwH 6 times for increasing durations again (10 min, 15 min, 30 min, 1 h, 2 h, overnight).

CGRP neuron stimulation time point samples also received primary (chicken anti-GFP, 1:500; Aves, GFP-1020) and secondary (Alexa Fluor 594 donkey anti-chicken, 1:500; Jackson Immunoresearch, 703–585-155) antibodies for ChR2–YFP immunolabelling during the above protocol.

#### Final storage and imaging.

Brain samples were serially dehydrated in increasing concentrations of methanol (20%, 40%, 60%, 80% and 100% in ddH_2_O; 45 min–1 h each), then incubated in a 2:1 solution of dichloromethane (Sigma, 270997) and methanol for 3 h then washed twice for 15 min in 100% dichloromethane. Before imaging, brains were stored in the refractive-index-matching solution dibenzyl ether (Sigma, 108014).

### FOS light-sheet microscopy imaging

Cleared and immunolabelled brain samples were glued (Loctite, 234796) ventral side-down to a 3D-printed holder and imaged in dibenzyl ether using a dynamic axial-sweeping light-sheet fluorescence microscope^74^ (Life Canvas Technologies, SmartSPIM) using SmartSPIM acquisition software (https://lifecanvastech.com/products/smartspim; v.5.6). Images were acquired using a ×3.6, 0.2 NA objective with a 3,650 × 3,650 μm field of view onto a 2,048 × 2,048 pixel sCMOS camera (pixel size, 1.78 × 1.78 μm) with a spacing of 2 μm between horizontal planes (nominal axial point spread function, 3.2–4.0 μm). Imaging of the entire brain required 4 × 6 tiling across the horizontal plane and 3,300–3,900 total horizontal planes. Autofluorescence channel images were acquired using 488 nm excitation light at 20% power (maximum output, 150 mW) and 2 ms of exposure time, and FOS channel images were acquired using 639 nm excitation light at 90% power (maximum output, 160 mW) and 2 ms of exposure time. For CGRP neuron stimulation time point samples, a bilateral volume encompassing both PB regions was imaged separately using 561 nm excitation light at 20% power (maximum output, 150 mW) and 2 ms of exposure time to confirm ChR2–YFP expression.

After acquisition, tiled images for the FOS channel were first stitched into a single imaging volume using the TeraStitcher C++ package^75^ (https://github.com/abria/TeraStitcher; v.1.11.10). These stitching parameters were then directly applied to the tiled autofluorescence channel images, which produced two aligned 3D imaging volumes with the same final dimensions. After tile stitching, striping artefacts were removed from each channel using the Python package Pystripe^76^ (https://github.com/chunglabmit/pystripe; v.0.2.0).

We registered the final FOS imaging volume to the Allen CCF using the autofluorescence imaging volume as an intermediary^73^. We first downsampled both imaging volumes by a factor of five for computational efficiency. Autofluorescence→atlas alignment was done by applying an affine transformation to obtain general alignment using only translation, rotation, shearing and scaling, followed by applying a b-spline transformation to account for local nonlinear variability among individual brains. FOS→autofluorescence alignment was done by applying only affine transformations to account for brain movement during imaging and wavelength-dependent aberrations. Alignment transformations were computed using the Elastix C++ package^77,78^ (https://github.com/SuperElastix/elastix; v.4.8). These transformations enabled us to transform FOS^+^ cell coordinates first from their native space to the autofluorescence space and then to Allen CCF space. In rare cases when this two-step alignment strategy failed, we directly registered the FOS imaging volume to the Allen CCF by applying both affine and b-spline transformations.

### Deep-learning-assisted cell-detection pipeline

We first use standard machine-vision approaches to identify candidate FOS^+^ cells based on peak intensity and then use a convolutional neural network to remove artefacts. Our pipeline builds on the Python package ClearMap^28,79^ (https://github.com/ChristophKirst/ClearMap2; v.2.0) for identifying candidate cells and the Python package Cellfinder^80^ (https://github.com/brainglobe/cellfinder; v.0.4.20) for artefact removal.

#### Cell detection.

ClearMap operates through a series of simple image-processing steps. First, the FOS imaging volume was background-subtracted using a morphological opening (disk size, 21 pixels). Second, potential cell centres were found as local maxima in the background-subtracted imaging volume (structural element shape, 11 pixels). Third, the cell size was determined for each potential cell centre using a watershed algorithm (see below for details on the watershed-detection threshold). Fourth, a final list of candidate cells was generated by removing all potential cells that were smaller than a preset size (size threshold, 350 pixels). We confirmed that our findings were consistent across a wide range of potential size thresholds.

We implemented three changes to the standard ClearMap algorithm. First, we de-noised the FOS imaging volume using a median filter (function, scipy.ndimage.median_filter; size, 3 pixels) before the background-subtraction step. Second, we dynamically adjusted the watershed-detection threshold for each sample based on its fluorescence intensity. This step was important for achieving consistent cell-detection performance despite changes in the background and signal intensity across batches and samples owing to technical variations in clearing, immunolabelling and imaging. In brief, we selected a 1,000 × 1,000 × 200 pixel subvolume at the centre of each sample’s FOS imaging volume. We then median-filtered and background-subtracted this subvolume as described above. We then used sigma clipping (function, astropy.stats.sigma_clipped_stats; sigma=3.0, maxiters=10, cenfunc=‘median’, stdfunc=‘mad_std’) to estimate the mean background signal level for this subvolume, *μ*_bg_, and set the watershed-detection threshold for each sample to 10*μ_bg_. Third, we removed from further analyses all cell candidates that were located outside the brain, in the anterior olfactory areas or cerebellum (which were often damaged during dissection), or in the ventricles, fibre tracts and grooves following registration to the Allen CCF.

#### Cell classification.

One limitation of the watershed algorithm implemented by ClearMap is that it identifies any high-contrast feature as a candidate cell, including exterior and ventricle brain edges, tissue tears, bubbles and other aberrations. To overcome this limitation, we re-trained the 50-layer ResNet^81^ implemented in Keras (https://keras.io; v.2.8.0) for TensorFlow (https://www.tensorflow.org; v.2.8.0) from the Python package Cellfinder^80^ to classify candidate FOS^+^ cells in our high-resolution light-sheet microscopy imaging dataset as true FOS^+^ cells or artefacts. This network uses both the autofluorescence and FOS channels during classification because the autofluorescence channel has significant information about high-contrast anatomical features and imaging aberrations. We first manually annotated 2,000 true FOS^+^ cells and 1,000 artefacts from each of four brain samples across two technical batches using the Cellfinder Napari plugin, which produced a total training dataset of 12,000 examples. We then re-trained the Cellfinder network (which had already been trained on approximately 100,000 examples from serial two-photon images of GFP-labelled neurons) over 100 epochs with a learning rate of 0.0001 and 1,200 examples (10% of the training dataset) held out for validation. Re-training took 4 days 16 min 41 s on a high-performance computing cluster using 1 GPU and 12 CPU threads. We achieved a final validation accuracy of 98.33%. Across all samples in our main brain-wide FOS dataset, our trained convolutional neural network removed 15.99 ± 0.58% (mean ± s.e.m.; range, 2.96–32.71%; *n* = 99 brains across the experiments in Figs. 1 and 2) of cell candidates from ClearMap as artefacts.

#### Atlas registration.

We used the ClearMap interface with Elastix to transform the coordinates of each true FOS^+^ cell to the Allen CCF space using the transformations described above. We then used these coordinates to assign each FOS^+^ cell to an Allen CCF brain region. For each sample, we generated a final data structure that contained the Allen CCF coordinates (*x*,*y*,*z*), size and brain region for each true FOS^+^ cell.

### FOS density maps

We generated 3D maps of FOS^+^ cell density by applying a Gaussian kernel-density estimate (KDE) (function, scipy.stats.gaussian_kde) in Python to all FOS^+^ cells across all animals in a given experimental condition (for example, novel flavour + consumption time point). These maps are visualized in Figs. 1h and 2k and Extended Data Figs. 1c–h, 2g and 6e,f.

We first generated a table containing the Allen CCF coordinates (*x*,*y*,*z*) for every FOS^+^ cell in every animal in an experimental condition. At this stage, we listed each cell twice (once with its original coordinates and once with its ML (*z*) coordinate flipped to the opposite hemisphere) to pool data from both hemispheres. We then assigned each cell a weight equal to the inverse of the total number of FOS^+^ cells in that animal to ensure that each animal in an experimental condition would be equally weighted. We then fit a 3D Gaussian KDE for each experimental condition using the scipy.stats.gaussian_kde function and manually set the kernel bandwidth for every experimental condition to be equal at 0.04. We then evaluated this KDE at every voxel in the Allen CCF (excluding voxels outside the brain or in anterior olfactory areas, cerebellum, ventricles, fibre tracts and grooves) to obtain a 3D map of FOS^+^ density for each condition. Last, we normalized the KDE for each experimental condition by dividing by its sum as well as the voxel volume of the atlas to generate a final 3D map with units of ‘per cent FOS^+^ cells per mm^3^’. For the CGRP neuron stimulation time point, we assigned each cell a weight equal to the inverse of the number of FOS^+^ cells in the PB of that animal, rather than the total number FOS^+^ cells, to account for variations in ChR2 expression across mice and flavour conditions.

To examine the difference in FOS^+^ cell density across flavour conditions (for example, in Extended Data Fig. 1d for the consumption time point) we simply subtracted the 3D KDE volumes for the two conditions, novel – familiar, and then plotted coronal sections through this subtracted volume with Allen CCF boundaries overlaid. The colour bar limits for all novel – familiar ΔFOS KDE figures are ±0.5% FOS^+^ cells per mm^3^ and for all average FOS KDE, figures are 0–1% FOS^+^ cells per mm^3^.

We used the WebGL-based Neuroglancer to generate interactive 3D visualizations of the FOS^+^ cell density maps for each experimental time point (https://www.brainsharer.org/ng/?id=872). To achieve this, we used the Python package cloudvolume (https://github.com/seung-lab/cloud-volume; v.8.5.1) to convert our 3D KDE volumes from the numpy format to precomputed layers compatible with Neuroglancer and then loaded these layers into the Brainsharer web portal to create the final visualization.

### FOS GLMMs

We adopted a GLMM to analyse the brain-wide FOS data (Figs. 1 and 2). This process enabled us to model the contribution of flavour and experimental time point to neural activation in each brain region while also accounting for the overdispersed, discrete nature of the data by using a negative binomial link function, the contribution of batch-to-batch technical variation in tissue clearing, immunolabelling and imaging by modelling this as a random effect, and the potential contribution of sex as a biological variable by modelling this as a fixed effect.

The first step was to determine whether there was any effect of novel or familiar flavour, experimental time point or their interaction for each brain region while controlling the false discovery rate (FDR) across all regions. To accomplish this, we fit a full GLMM for each brain region using the R package glmmTMB^69^ (https://github.com/glmmTMB/glmmTMB; v.1.1.7) with a negative binomial link function (nbinom2) and the formula:

(2) FOScounts~Novel*Timepoint+Sex+(1|Batch)+ln(Totalcounts)

where FOS counts is the number of FOS^+^ cells in a brain region, Novel (novel, familiar), Time point (consumption, malaise, retrieval) and Sex (female, male) are fixed-effect categorical variables, (1|Batch) is a random effect for each technical batch (that is, each set of samples that underwent tissue clearing, immunolabelling and light-sheet microscopy imaging together), ln(Total counts) is an offset term for the total number of FOS^+^ cells in each sample and the asterisk represents all possible main effects and interactions (Fig. 1d). We then fit a reduced GLMM for each brain region, which was the same as the full model (equation (2)) but with the Novel*Time point terms (that is, all main effects and interactions related to flavour novelty and experimental time point) removed. We compared these two models for each brain region using likelihood-ratio χ^2^-tests and then adjusted the resultant *P* values using the Benjamini–Krieger–Yekutieli two-step procedure^82^ to permit a 10% FDR across all brain regions. The 10% FDR threshold used here is standard for brain-wide FOS studies^83–85^. Of the 200 brain regions tested, 130 met this criterion and were included for downstream analyses.

We next specifically tested the effect of flavour novelty on FOS counts separately at each experimental time point for the 130 brain regions that passed the above-defined FDR threshold. To calculate the marginal effect of the flavour condition (novel – familiar) at each time point for each brain region, we used the R package marginaleffects^70^ (https://github.com/vincentarelbundock/marginaleffects; v.0.12.0) to do post hoc testing of the full GLMM. We used the marginal effect estimates and s.e. values to calculate a *P* value for each time point with a *z* test and then corrected for multiple comparisons across time points in each brain region using the Hochberg–Bonferroni procedure^71^. We also used the ratio of these marginal effect estimates and s.e. values to compute the standardized average difference in FOS^+^ cell counts across flavour conditions for each brain region at each time point (*Z* = estimate/s.e.; Fig. 1e–g and Supplementary Table 1). The advantage of this metric is that it explicitly accounts for variation within and across groups, for effects of sex and technical batch, and is independent of brain region size.

When displaying FOS^+^ cell counts for individual samples (Fig. 1i and Extended Data Figs. 1a,b and 6b), we divided the number of FOS^+^ cells for each animal or brain region by the total number of FOS^+^ cells in that animal and by the Allen CCF volume of that brain region, so that the data for each region are presented as ‘per cent FOS^+^ cells per mm^3^’. We used the *P* values from the GLMM marginal effect *z* tests described above to assess significance.

To examine the brain-wide shift in novel – familiar coding across time points (Fig. 1e and Extended Data Fig. 3a–c), we used the Matlab package Violinplot (https://github.com/bastibe/Violinplot-Matlab) to plot the distribution of standardized average difference *Z* values at each time point for the brain regions that passed our FDR threshold and then used Kolmogorov–Smirnov tests to assess whether these distributions were significantly different from each other, correcting for multiple comparisons across time points using the Hochberg–Bonferroni procedure^71^.

To identify structure in novel – familiar coding across time points (Fig. 1f and Extended Data Fig. 3d–n), we used the built-in Matlab linkage function (method=‘ward’, metric=‘chebychev’) to create a hierarchical tree using the standardized average difference *Z* values at each time point for the brain regions that passed our FDR threshold. The input matrix was 130 brain regions × 3 time points. We then used the built-in Matlab dendrogram function to plot this hierarchical tree and used a distance threshold of 4.7 for clustering.

We followed an analogous procedure to analyse brain-wide FOS data for the CGRP neuron stimulation time point (Fig. 2 and Extended Data Fig. 6). To account for variations in ChR2 expression across mice and flavour conditions, we weighted FOS^+^ cell counts by the number of FOS^+^ cells in the PB in these analyses. Specifically, to compare the effects of CGRP neuron stimulation and LiCl-induced malaise on overall FOS levels (Extended Data Fig. 6c), we fit a GLMM for each brain region with the negative binomial link function and the formula:

(3) FOScounts~Timepoint+Sex+(1|Batch)+ln(PBcounts)

where FOS counts is the number of FOS^+^ cells in a brain region, Time point (consumption, malaise, retrieval, CGRP neuron stimulation) and Sex (female, male) are fixed-effect categorical variables, (1|Batch) is a random effect for each technical batch, ln(PB counts) is an offset term for the total number of FOS^+^ cells in the PB of each sample and the asterisk represents all possible main effects and interactions. For this model, we did not include any terms related to flavour novelty because we were specifically investigating changes in overall FOS levels. We then plotted the coefficient estimate *Z* values from this GLMM (Extended Data Fig. 6c). To compare the effects of CGRP neuron stimulation and LiCl-induced malaise on FOS levels in the novel versus familiar flavour condition (Extended Data Fig. 6d), we fit a GLMM for each brain region with the formula:

(4) FOScounts~Novel*Timepoint+Sex+(1|Batch)+ln(PBcounts)

where FOS counts is the number of FOS^+^ cells in a brain region, Novel (novel, familiar), Time point (malaise, CGRP neuron stimulation) and Sex (female, male) are fixed-effect categorical variables, (1|Batch) is a random effect for each technical batch, ln(PB counts) is an offset term for the total number of FOS^+^ cells in the PB of each sample and the asterisk represents all possible main effects and interactions. For this model, we only included the experimental time points in which CGRP neurons were activated either optogenetically (CGRP neuron stimulation) or pharmacologically (malaise); see Extended Data Fig. 6b for the quantification of PB activation. We then calculated and plotted the marginal effect of the flavour condition (novel – familiar) separately for each time point and brain region (Extended Data Fig. 6d). We also used the marginal effect from the GLMM in equation (4) to calculate the *P* value for Fig. 2h. When displaying FOS^+^ cell counts for individual animals (Fig. 2h; CGRP neuron stimulation time point) or brain regions (Fig. 2i,j; malaise and CGRP neuron stimulation time points), we first divided the number of FOS^+^ cells for each brain region in each animal by the number of FOS^+^ cells in the PB for that animal and by the Allen CCF volume of that brain region. We then divided this number by the average ratio of total FOS^+^ cells to PB FOS^+^ cells across every sample in that time point (malaise or CGRP neuron stimulation), which produced a final measure of FOS^+^ cells of each animal or region as a percentage of the entire brain’s FOS^+^ cells weighted by the relative count of PB FOS^+^ cells for that animal. We obtained consistent results by instead subsampling the animals in the CGRP neuron stimulation time point to have approximately equal FOS^+^ cell counts in both flavour conditions and then weighting by the total FOS^+^ cell count of each animal.

### FOS correlation analysis

To quantify FOS correlations across individual mice (Extended Data Fig. 4), we considered each experimental time point (consumption, malaise, retrieval) separately. We first assembled the relative FOS^+^ cell counts (per cent per mm^3^) for every brain region that passed our FDR threshold and then sorted these regions using the hierarchical tree fit described above, which resulted in a 130 brain region × 24 animal input matrix for each experimental time point. We then used the built-in Matlab corr function to calculate and visualize pairwise correlations among all brain regions (Extended Data Fig. 4a). To estimate the correlation among individual brain regions in the amygdala cluster at each time point (Extended Data Fig. 4b), we averaged pairwise correlations for each brain region with all other amygdala cluster regions in the correlation matrices described above. We tested whether the correlation among individual amygdala cluster brain regions was significant at each time point using Wilcoxon signed-rank tests, correcting for multiple comparisons across time points using the Hochberg–Bonferroni procedure^71^. To estimate the correlation between the amygdala cluster and every other cluster at each time point (Extended Data Fig. 4c), we averaged the pairwise correlations for all brain region pairs across the two clusters.

### LS activation FOS analysis

To compare the effects of LS activation on FOS levels in the LS and the CEA (Extended Data Fig. 2d,e), we calculated *P* values for these two regions using a GLMM with the formula:

(5) FOScounts~hM3D+Sex+(1|Batch)+ln(Totalcounts)

where FOS counts is the number of FOS^+^ cells in a brain region, hM3D (hM3D, YFP) and Sex (female, male) are fixed-effect categorical variables, (1|Batch) is a random effect for each technical batch, ln(Total counts) is an offset term for the total number of FOS^+^ cells in each sample and the asterisk represents all possible main effects and interactions. The *P* values in the figure are from the hM3D coefficient estimates.

To compare the effects of LS activation of FOS levels across the brain (Extended Data Fig. 2f), we plotted the average FOS level (per cent FOS^+^ cells per mm^3^) across all mice in each condition (hM3D, YFP) separately for three groups of brain regions: the amygdala network, the septal complex and other regions. We then used the built-in Matlab aoctool function to fit a one-way analysis of covariance model for these three groups of brain regions and the built-in Matlab multcompare function to test whether the estimated slopes were significantly different, correcting for multiple comparisons using the Bonferroni procedure.

### RNAscope FISH

We sliced 18–25-μm-thick sections from perfused brain samples. Multiplex FISH (Fig. 2l–n and Extended Data Fig. 6g,h) was performed using an RNAscope^86^ Multiplex Fluorescent Assay v2 (ACD 323120) with the following probes: Mm-Calcrl (452281), Mm-Sst-C2 (404631-C2, 1:50 dilution in C1 solution), Mm-Prkcd-C3 (441791-C3, 1:50 dilution in C1 solution) and Mm-Fos-C4 (316921-C4, 1:50 dilution in C1 solution). The *Calcrl*, *Sst*, *Prkcd* and *Fos* probes were linked to Opal 690, Opal 520, Opal 620 and Opal 570 fluorophores, respectively (Akoya Biosciences). All fluorophores were reconstituted in DMSO according to instructions from the manufacturer and diluted 1:1,200 in tyramide signal amplification buffer included in the RNAscope kit. After in situ hybridization, slides were coverslipped using DAPI Fluoromount-G (Southern Biotech, 0100).

We obtained ×20 *z* stacks from the CEA with a confocal microscope (Leica TCS SP8 X) using Leica Application Suite X software (https://www.leica-microsystems.com; v.1.8). We then converted these *z* stacks into maximum-intensity projections for each labelled RNA. We trained a Cellpose^87,88^ (https://cellpose.readthedocs.io; v.3.0.8) model to identify *Fos*^+^ cells in the maximum-intensity projections using eight manually corrected examples, and then used this model to identify *Fos*^+^ cells in the remaining images. We then manually classified whether every *Fos*^+^ cell identified by Cellpose also expressed *Sst*, *Prkcd* and/or *Calcrl*. We used the full *z* stacks for each labelled RNA for this process to ensure that potentially overlapping cells were labelled separately. We also imaged each tissue section with a slide scanner (Hamamatsu, Nanozoomer S60) using NDP Scan software (https://www.hamamatsu.com; v.3.4) and then registered them to the Allen CCF using ABBA^89^ (https://abba-documentation.readthedocs.io; v.0.8.0). To remove *Fos*^+^ cells outside the CEA from analysis, we manually aligned the confocal and slide scanner images using the *Fos* channel in each image as a guide and then manually transferred the CEA boundaries to the confocal images. Manual cell classifications and basic image processing tasks were performed using Fiji^72^ (https://fiji.sc; v.1.52).

### Slice electrophysiology

All slice electrophysiology recordings (Fig. 2c) were performed on brain slices collected at approximately the same time of day. *Calca*^*cre*^ mice were first injected with 400 nl of AAV5-EF1a-DIO-hChR2(H134R)-eYFP (titre, 1.2 × 10^13^ GC per ml; manufacturer, PNI Viral Core Facility) bilaterally into the PB 6 weeks or more before the experiment. On the day of the recordings, mice were anaesthetized with isoflurane and decapitated to remove the brain. After extraction, the brain was immersed in ice-cold NMDG ACSF (92 mM NMDG, 2.5 mM KCl, 1.25 mM NaH_2_PO_4_, 30 mM NaHCO_3_, 20 mM HEPES, 25 mM glucose, 2 mM thiourea, 5 mM sodium ascorbate, 3 mM sodium pyruvate, 0.5 mM CaCl_2_·4H_2_O, 10 mM MgSO_4_·7H_2_O and 12 mM *N*-acetyl-l-cysteine; pH adjusted to 7.3–7.4) for 2 min. Afterwards, coronal slices (300 μm) were sectioned using a vibratome (Leica VT1200s) and then incubated in NMDG ACSF at 34 °C for approximately 15 min. Slices were then transferred to a holding solution of HEPES ACSF (92 mM NaCl, 2.5 mM KCl, 1.25 mM NaH_2_PO_4_, 30 mM NaHCO_3_, 20 mM HEPES, 25 mM glucose, 2 mM thiourea, 5 mM sodium ascorbate, 3 mM sodium pyruvate, 2 mM CaCl_2_·4H_2_O, 2 mM MgSO_4_·7H_2_O and 12 mM *N*-acetyl-l-cysteine, bubbled at room temperature with 95% O_2_ and 5% CO_2_) for at least 60 min until recordings were performed.

Whole-cell recordings were performed using a Molecular Devices Multiclamp 700B amplifier and Digidata 1440A low-noise data acquisition system. Recording pipettes had a resistance of 4–7 MΩ and were filled with an internal solution containing 120 mM potassium gluconate, 0.2 mM EGTA, 10 mM HEPES, 5 mM NaCl, 1 mM MgCl_2_, 2 mM Mg-ATP and 0.3 mM NA-GTP, with the pH adjusted to 7.2 with KOH and the osmolarity adjusted to approximately 289 mmol kg^−1^ with sucrose. During recordings, slices were perfused with a recording ACSF solution (100 μM picrotoxin, 120 mM NaCl, 3.5 mM KCl, 1.25 mM NaH_2_PO_4_, 26 mM NaHCO_3_, 1.3 mM MgCl_2_, 2 mM CaCl_2_ and 11 mM D-(+)-glucose) with 1 μM TTX and 100 μM 4AP that was continuously bubbled with 95% O_2_ and 5% CO_2_. Infrared differential interference contrast-enhanced visual guidance was used to select neurons that were 3–4 cell layers below the surface of the slices. All CEAc and CEAl recordings were made for which eYFP-expressing CGRP neuron axons were visible, and all CEAm recordings were made more medial to this location using the Allen CCF as a guide The recording solution was delivered to slices through superfusion driven by a peristaltic pump (flow rate of 4–5 ml min^−1^) and was held at room temperature. The neurons were held at −70 mV (voltage clamp), and the pipette series resistance was monitored throughout the experiments by hyperpolarizing steps of 1 mV with each sweep. If the series resistance changed by >20% during the recording, the data were discarded. Whole-cell currents were low-pass filtered at 4 kHz online and digitized and stored at 10 kHz using Clampex software (https://www.moleculardevices.com; v.10.7). Currents were then filtered at 1 kHz offline before analysis. During the experiment, we measured light-evoked oEPSCs every 30 s with light stimulation (0.074 mW mm^−2^) delivered for a duration of 5 ms. Twenty repetitions of the stimulation protocol were recorded per cell after stable oEPSCs were achieved. All experiments were completed within 4 h after slices were made to maximize cell viability and consistency.

Traces from example CEAc, CEAl and CEAm neurons are shown in Fig. 2c. Across all monosynaptically connected neurons, the amplitude of CGRP neuron→CEAc or CEAl oEPSCs was −327.0 ± 136.3 pA (mean ± s.e.m.; *n* = 5 out of 5 connected neurons from 3 mice) and of CGRP neuron→CEAm oEPSCs was −15.6 ± 6.4 pA (mean ± s.e.m.; *n* = 4 out of 5 connected neurons from 3 mice).

### Two-reward CFA paradigm

For Neuropixels recording experiments (Figs. 3 and 4), we used a two-reward CFA paradigm (rather than the one-reward paradigm used for the FOS experiments). This enabled us to compare neural correlates of the novel-flavour reward, which was delivered from one port, with responses related to a control port that delivered water. Experiments were performed in an operant box (Med Associates) using MedPC software (https://med-associates.com/product/med-pc; v.IV). The operant box was situated in a sound-attenuating chamber and equipped with a single speaker and with two custom 3D-printed nosepoke ports with built-in lights and infrared beam breaks in each port. The nosepoke ports each contained a reward delivery tube that was calibrated to deliver 20 μl of reward through a solenoid valve (Lee Technologies LHDA2433315H). The ports were located on either side of the same wall of the operant box.

#### Basic task structure and training.

Mice first underwent a basic task procedure to train them to drink from two reward ports in a cued manner (Extended Data Fig. 8a). Each behavioural session had the following structure. First, the mouse was allowed to acclimate to the chamber for 5 min. Then, the consumption period began and rewards were made available in a trial-based manner that forced mice to drink from the two ports at a relatively equal rate throughout the session. At the beginning of each trial, one port was randomly selected and made available to the mouse. This was cued through the port light turning on and a distinct tone (2.5 kHz or 7.5 kHz; 70 dB) playing. The mouse had 10 s to enter the port and receive a reward, which was detected by the infrared beam break. The end of the 10 s reward availability period or entering either port ended the trial; at this point, the cueing light and sound were terminated and an inter-trial interval was initiated (randomly selected from a uniform distribution of 10–20 s; 1 s step size). At the end of the inter-trial interval, a new trial would begin as long as the mouse had not entered either reward port in the previous 2 s; otherwise, the next trial was delayed until this criterion was satisfied. To ensure that mice drank from the two ports at a relatively equal rate, we required that each consecutive block of ten successful rewarded trials must be evenly split between the two ports. The consumption period ended when 1.2 ml (60 rewards) was consumed. Mice learned to perform this task nearly perfectly (<5 unsuccessful trials per session) in approximately 1 week. During initial training, both ports delivered water. After mice were trained in the task, they underwent the chronic Neuropixels surgery described above and were allowed to recover for at least 5 days. Mice were then returned to daily training, with the addition of a delay period following the consumption period. At the beginning of the delay period (immediately after the final reward), mice were transferred to a distinct second context, which was triangular in shape with smooth white acrylic walls. Mice remained in this second context for at least 30 min before returning to the home cage. Mice were acclimated to the delay period and second context, and to tethering of the Neuropixels assembly and optical fibre, for at least 4 days before proceeding to the conditioning experiments. Variations to this basic task structure for specific experiments that were used following training and surgery are described below.

#### CGRP neuron cell-body stimulation conditioning experiment.

For these experiments (Figs. 3 and 4), on the conditioning day, the same behavioural session structure was followed, but now one port delivered water and the other port delivered sweetened grape Kool-Aid (0.06% grape and 0.3% saccharin sodium salt). The novel-flavour port was counterbalanced across mice. Mice (*n* = 8) were run in two separate groups separated by approximately 2 months. After the 30-min delay period in the second context, the CGRP neuron stimulation period began and lasted for 45 min in the same second context. Blue light was generated using a 447 nm laser and delivered to the animal using a 200 μm diameter patch cable. Light power was calibrated to approximately 10 mW at the patch cable tip. The laser was controlled with a Pulse Pal signal generator programmed to deliver 5 ms laser pulses at 10 Hz. For the duration of the CGRP neuron stimulation period, the laser was pulsed for 3 s bouts and then off for random intervals chosen from an exponential distribution (minimum, 1 s; mean, 3 s; maximum, 7.8 s). Following the 45-min neuron stimulation period, mice were returned to the home cage overnight. The following day, mice underwent a forced retrieval session that followed the same trial structure as previous sessions, and the flavour and water were delivered from the same ports as on the conditioning day.

#### CGRP^CEA^ projection stimulation conditioning experiment.

These experiments (Fig. 4e and Extended Data Figs. 9a–e and 10c–e) were performed using the same strategy as the cell-body stimulation experiment described above in a separate group of mice (*n* = 8) with the following changes. Green light was generated using a 532-nm laser and calibrated to approximately 3 mW at the patch cable tip. To minimize potential photoelectric artefacts in our recordings, we positioned the tip of the optical fibre 1.5 mm from the Neuropixels shanks in the CEA for an irradiance at the electrodes of approximately 0.1 mW mm^−2^ and reduced the laser pulse width to 2 ms. These stimulation parameters were sufficient to activate the ultrasensitive opsin ChRmine^64^.

#### LiCl conditioning experiment.

This experiment (Fig. 3m–r and Extended Data Figs. 9f,g and 10f,g) was performed in a separate group of mice (*n* = 4 control mice and 4 CGRP neuron ablation mice). It followed the same structure as above except that LiCl (125 mg kg^−1^ i.p.) was injected to induce gastrointestinal malaise after 30 min in the second context (delay period) instead of CGRP neuron stimulation. For behavioural validation of CGRP neuron ablation (Extended Data Fig. 9g), we included five mice that were not used for recordings but either received taCasp3 virus (*n* = 2 ablation mice) or did not undergo surgery (*n* = 3 control mice).

#### Familiarization experiment.

For these experiments (Fig. 4f and Extended Data Fig. 10h–k), a different flavour, sweetened cherry Kool-Aid (0.06% cherry and 0.3% saccharin sodium salt), was used. The experiment followed the same basic task structure as during initial training for the two-reward CFA paradigm, without any aversive conditioning experiences (LiCl injection or CGRP neuron stimulation). The experiment was run on three consecutive days. On the first day (novel day), one port contained the novel sweetened cherry Kool-Aid flavour and the other port contained water. On the second day, the port locations were switched. On the third day (familiar day), the port locations were switched again (that is, back to the initial locations from novel day).

#### CGRP neuron stimulation and LiCl injection experiment.

This experiment (Extended Data Fig. 9h–j) did not involve rewards or use the task structure described above. First, mice were allowed to acclimate to the operant box recording chamber for 5 min. Then, CGRP neurons were photostimulated using the same protocol as for the acute Neuropixels recording experiment described below. In brief, mice received 1 s of 10 Hz CGRP neuron stimulation followed by a 9-s inter-trial interval for a total of 10 min per 60 trains. After a 5-min recovery period, LiCl (125 mg kg^−1^ i.p.) was then injected to induce gastrointestinal malaise. Mice remained in the recording chamber for at least 15 min before being returned to the home cage.

We used 27 mice for chronic Neuropixels recording experiments (Extended Data Fig. 7c). Animals 1–4 were used for the CGRP neuron cell-body stimulation conditioning experiment. Animals 5–8 were used for multiple experiments with the following timeline: (1) CGRP neuron cell-body stimulation conditioning experiment, (2) familiarization experiment, (3) CGRP neuron stimulation→LiCl injection experiment. Animals 9–12 were control mice used for the LiCl conditioning experiment. Animals 13–16 were CGRP neuron ablation mice used for the LiCl conditioning experiment. Animals 17–24 were used for the CGRP^CEA^ projection stimulation conditioning experiment. Animals 25–27 were used for the familiarization experiment.

### Chronic Neuropixels recordings

Before beginning experiments, we performed a series of test recordings for each mouse to identify the recording sites along each Neuropixels shank that were located in the CEA. We recorded for approximately 10 min from the bottom 384 recording sites of each shank. We found that recording sites properly targeted to the CEA could be identified by a dense band of single-unit and multiunit activity (see Extended Data Fig. 7b for examples). This process enabled us to design custom Imec readout tables (recording site maps; https://billkarsh.github.io/SpikeGLX/help/imroTables) for each mouse that maximized the yield of CEA neurons during subsequent experiments.

#### Acquisition.

We recorded 384 Neuropixels channels per session at 30 kHz using National Instruments PXI hardware and SpikeGLX software (https://billkarsh.github.io/SpikeGLX; v.3.0). Experimental TTL signals (representing reward cues, port entries, reward deliveries and laser pulses) were recorded simultaneously using the same system.

#### Preprocessing.

We used CatGT (https://billkarsh.github.io/SpikeGLX; v.3.3) to apply global common average referencing (-gblcar) and to isolate the action potential frequency band (-apfilter=butter,12,300,9000). We then used the International Brain Laboratory’s (IBL) Python Kilosort 2.5 implementation^90,91^ (https://github.com/int-brain-lab/pykilosort) to correct for sample drift along the length of the probe, to detect and remove failing channels and to apply a spatial de-striping filter.

#### Spike sorting and curation.

We also used the IBL’s Python Kilosort 2.5 implementation^91–93^ (https://github.com/int-brain-lab/pykilosort) for spike sorting. We then used the Python package Phy (https://github.com/cortex-lab/phy; v.2.0) for interactive visualization and manual curation of spike sorting output. We used Phy to classify clusters from Kilosort as single-unit (good) or multiunit (MUA) clusters and to remove noise. We relied on waveform shape, autocorrelogram shape, spike amplitude time course and cluster separation for classification. Following curation with Phy, we used Matlab to compute three statistics for each cluster. First, we calculated the median amplitude of each cluster using the template scaling amplitudes from Kilosort (stored in amplitudes. npy), converted from bits to μV using the gain factor 2.34375. These template-scaling amplitudes were calculated after whitening the data and were significantly smaller than the equivalent raw spike amplitudes (μV). Second, we calculated the estimated false-positive rate of each cluster based on 2 ms refractory period violations^94^. Third, we calculated the firing rate (sp s^−1^) of each cluster. For experiments with multiple epochs (for example, consumption and CGRP neuron stimulation in Fig. 3), we calculated these metrics separately for each epoch and then kept the minimum median template-scaling amplitude, the maximum estimated false-positive rate and the minimum firing rate across epochs for each cluster. We removed clusters with median template-scaling amplitude values of <20 μV, estimated false-positive rates of >100% or firing rates <0.05 sp s^−1^ as noise. We classified the remaining clusters that were labelled ‘good’ in Phy and had an estimated false-positive rate <10% as single units and the rest as multiunits. We included both single-unit and multiunit clusters throughout the article. We confirmed that our findings were consistent across a range of amplitude thresholds and for only single-unit clusters. Finally, we binned the spikes for each included neuron into 10 ms bins for downstream analyses.

#### Atlas alignment.

All probes were coated in CellTracker CM-DiI (Invitrogen C7000) before implantation. After the conclusion of experiments, animals were euthanized and the brains cleared with an abbreviated version of the iDISCO+ protocol described above without immunostaining. The cleared brains were then imaged on a light-sheet microscope (LaVision Ultramicroscope II) using LaVision BioTec ImSpector software (https://www.lavisionbiotec.com; v.7.0). Images were acquired using 488 nm (autofluorescence channel) and 561 nm (CM-DiI channel) excitation light with 10 μm between horizontal planes and 5.91 μm per pixel resolution. Atlas alignment then followed the IBL’s pipeline^95^ (https://github.com/int-brain-lab/iblapps/wiki). The autofluorescence volume was registered to the atlas using the Python package Brainreg^96^ (https://github.com/brainglobe/brainreg; v.0.4.0), and these transformations were then directly applied to the CM-DiI volume. Individual Neuropixels shank trajectories were then manually annotated in the atlas-registered CM-DiI volume using the Brainreg-segment Napari module (https://github.com/brainglobe/brainreg-segment; v.0.2.16). Every recording site was then localized to an Allen CCF coordinate (*x*,*y*,*z*) and brain region using the IBL’s alignment GUI. The alignment process was performed separately for each Neuropixels probe shank. For all analyses, we only included neurons from recording sites that were localized to the CEA.

#### Multiday recordings.

We made two changes to our processing pipeline to track units across two recording sessions (for example, the conditioning and retrieval sessions in Fig. 4b–e and Extended Data Fig. 10a–g, and the novel and familiar sessions in Fig. 4f and Extended Data Fig. 10h–k). First, we concatenated the two recording sessions using CatGT after applying global common average referencing and isolating the action potential frequency band. We then performed spike sorting using the IBL’s Python Kilosort 2.5 implementation as described above. Second, during manual curation in Phy, we removed clusters with obvious discontinuities or irregularities across days as noise. We then evaluated our quality metrics in Matlab as described above. To improve Kilosort’s ability to track units in multiday recordings, pairs of sessions were separated by only 1 day (conditioning and retrieval) or 2 days (novel and familiar). We then used the Matlab package Spikes (https://github.com/cortex-lab/spikes) to extract spike waveforms and to generate autocorrelograms for each recording session (Fig. 4a).

### Chronic Neuropixels analysis

For experiments involving reward delivery, we classified all neurons as novel-flavour-preferring, water-preferring or nonselective (for example, in the heatmaps in Figs. 3d and 4b). We first *z* scored each neuron’s 10-ms binned spiking across the entire consumption period. We then calculated the average neural activity in the 10 s following every reward delivery, which was triggered by the animal entering the port. We distinguished nonselective neurons from reward-selective neurons using a Wilcoxon rank-sum test on these average responses for novel flavour and water trials while permitting a 5% FDR across all recorded neurons (pooled across mice within each experiment) with the Benjamini–Krieger–Yekutieli procedure^82^. We then classified reward-selective neurons as novel-flavour-preferring if their average neural activity in the 10 s following reward delivery was greater for novel-flavour trials than for water trials; the remaining neurons were classified as water-preferring. When tracking neurons across days and examining the change in their flavour response or selectivity (Fig. 4 and Extended Data Fig. 10), we classified neurons as novel-flavour-preferring or water-preferring based on their responses during consumption on the first day (novel or conditioning) only. We defined ‘novel flavour response’ as the average neural activity in the 10 s following novel flavour delivery and ‘novel flavour selectivity’ as the average neural activity in the 10 s following novel flavour delivery minus the average neural activity in the 10 s following water delivery. When correlating CGRP response to the change in flavour response and selectivity across days (Fig. 4d,e), we subtracted the baseline activity (−10 s to −5 s before reward delivery) from each trial when calculating reward responses to account for potential changes in baseline firing rate across days.

We generated peri-event time histograms (PETHs) surrounding reward delivery (−5 s to +10 s) using the *z* scored traces calculated above and averaging across all novel-flavour or water reward deliveries. When generating reward PETHs for the second day of multiday recordings (retrieval day, familiar day), we used the mean and s.d. calculated while *z* scoring the consumption period trace for the first day to ensure that units were comparable across days. We generated PETHs surrounding CGRP neuron stimulation or CGRP^CEA^ projection stimulation trains (−1 s to +4 s) using the mean and s.d. calculated while *z* scoring that day’s consumption period trace, and then subtracted the baseline (−1 s to 0 s) mean of each neuron’s PETH. For plotting reward delivery PETHs as heatmaps and traces, we convolved each neuron’s PETH with a causal half-Gaussian filter with 100-ms s.d.

We generated delay→CGRP neuron stimulation PETHs (Figs. 3d,e and 4b, c), delay→CGRP^CEA^ projection stimulation PETHs (Extended Data Figs. 9b,c and 10c) and delay→LiCl PETHs (Fig. 3n,q) using the 10-ms binned spiking from 30 min before to 45 min after the onset of CGRP neuron stimulation or CGRP^CEA^ projection stimulation LiCl injection. We then *z* scored these traces using the mean and s.d. from the final 20 min of the delay period and downsampled the final normalized PETHs to 1 sample per min for plotting. We defined ‘CGRP response’ as the average neural activity across the entire 45-min CGRP neuron stimulation or CGRP^CEA^ projection stimulation period in the PETHs described above (Figs. 3e and 4c–e and Extended Data Fig. 9c) and ‘LiCl response’ as the average neural activity from 5–15 min after LiCl injection in the PETHs described above (Fig. 3n,q). We generated whole-experiment PETHs (Fig. 3e,n,q and Extended Data Fig. 9c) by concatenating the 10-ms binned spiking from the final 15-min of the consumption period, the first 30-min of the delay period and the first 45-min of the CGRP neuron stimulation period or CGRP^CEA^ projection stimulation period or LiCl-induced malaise period. We then *z* scored these traces using the mean and s.d. of the delay period calculated above and downsampled the final normalized PETHs to 1 sample per min for plotting. When comparing the CGRP response (Fig. 3e,f and Extended Data Fig. 9c,d) or LiCl response (Fig. 3n,q) of novel flavour-preferring, water-preferring and nonselective neurons, we corrected for multiple comparisons using the Hochberg–Bonferroni procedure^71^.

For the CGRP neuron stimulation→LiCl injection experiment (Extended Data Fig. 9h–j), we first classified each neuron’s CGRP response type using the strategy from the acute recording experiment described below. We first *z* scored each neuron’s 10-ms binned spiking across the entire 10-min CGRP neuron stimulation period and generated baseline-subtracted PETHs surrounding CGRP neuron stimulation trains (−1 s to +2 s). We then applied the Gaussian mixture model (GMM) fit on the acute recording data (Extended Data Fig. 5c) to these PETHs to determine each neuron’s CGRP response type and to identify CGRP-activated neurons (Extended Data Fig. 9i). We then generated LiCl injection PETHs using the 10-ms binned spiking from 5 min before to 15 min after the LiCl i.p. injection. We then *z* scored these traces using the mean and s.d. from the 5-min acclimatization period before CGRP neuron stimulation began and baseline-subtracted the normalized PETHs using the mean activity during the period between CGRP neuron stimulation and LiCl i.p. injection (−5 min to −1 min before LiCl). We downsampled the final normalized PETHs to 1 sample per min for plotting and then plotted average LiCl injection PETHs separately for CGRP-activated neurons and for other neurons (Extended Data Fig. 9j). We defined ‘LiCl response’ as the average neural activity from 5 to 15 min after LiCl injection in the PETHs described above (Extended Data Fig. 9j).

### Decoding analysis

To identify reactivations of neural flavour representations (Fig. 3g–i,o,r), we trained a multinomial logistic regression decoder using the LogisticRegression class from the Python package scikit-learn^97^ (https://scikit-learn.org; v.1.0.2) separately for each mouse using all CEA neurons during the consumption period on the conditioning day and then evaluated this decoder across the entire conditioning session. We included all mice with >75 simultaneously recorded CEA neurons for the decoding analysis (6 out of 8 CGRP neuron stimulation mice in Fig. 4h–i; 8 out of 8 LiCl injection mice in Fig. 4o,r). The decoder was trained to discriminate behavioural states during the consumption period across three categories: novel-flavour consumption (represented by normalized spike counts within 1 s after novel-flavour delivery; normalization procedure described below); water consumption (normalized spike counts within 1 s after water delivery); and baseline (normalized spike counts within 1 s before each cue onset). We trained the decoder using Lasso regularization and tested *λ* from 10^−4^ to 10^4^ (nine logarithmically spaced values; Extended Data Fig. 8c). We chose *λ* = 1 for the final decoder because it provided a high level of regularization without decreasing log-likelihood in the held-out data during tenfold cross-validation. Cross-validation also verified that the decoder correctly identified the animal’s behavioural state (novel flavour, water, baseline) during the consumption period (Extended Data Fig. 8e).

We normalized spike counts separately for each neuron and task period. For the consumption period (decoder training), we calculated each neuron’s average spike counts within 1 s before cue onsets and subtracted it from the binned spike counts. We then divided these baseline-subtracted spike counts by the s.d. during the consumption period. For the delay and CGRP neuron stimulation periods (decoder evaluation), we *z* scored each neuron’s spike counts based on its mean and s.d. during the delay period.

We then evaluated the decoder using neural activities across the session. We first used a 1-s sliding window with 150 ms steps to bin the spikes across the start to the end of the session. After obtaining the *n* neuron × *n* time bin normalized spike counts, we used the decoder to classify the behavioural state for each time bin based on the corresponding normalized spike counts. To visualize the decoder’s performance (Fig. 3g), we plotted the decoder output along with the simultaneously recorded neural activity (spike trains convolved with a causal half-Gaussian filter with 25 ms s.d.) grouped by novel flavour or water preference, defined using criteria described above. For clarity, we only display a subset of recorded neurons (50 out of 90) in the example raster in Fig. 3g: all novel flavour-preferring and water-preferring neurons, along with 15 randomly chosen nonselective neurons. We focused our analysis on the comparison between the decoded probabilities for the novel flavour and water categories (Fig. 3g, top, and Fig. 3h). We detected peaks (local maxima with values > 0.5) of the decoder output as reactivation events, and counted the number of novel flavour and water reactivations with a sliding window of 1 min width and 30 s step size (Fig. 3i,o,r).

### PCA

We used the built-in Matlab pca function (Fig. 3j). We began by taking the novel flavour delivery, water delivery and CGRP neuron stimulation PETHs described above, all convolved with a causal half-Gaussian filter with 100 ms s.d., for all reward-selective neurons (*n* = 494 pooled across all mice). We baseline-subtracted each neuron’s reward-delivery PETHs using the mean baseline activity (−5 s to −4 s before reward delivery) averaged across both reward types and then peak-normalized each neuron’s PETHs using the maximum absolute value across both reward types. We baseline-subtracted each neuron’s CGRP neuron stimulation PETH using the mean baseline activity (−1 s to 0 s) before laser onset and then peak-normalized each neuron’s PETH using the maximum absolute value of the CGRP neuron stimulation PETH. To identify PC loadings and to calculate the variance explained, we concatenated each neuron’s novel flavour and water reward PETHs (0 s to +5 s from reward delivery), which produced a final input matrix that was 494 neurons × 1,000 time bins for PCA. We centred every column of this matrix before performing PCA along the neuron dimension.

To plot neural trajectories during novel flavour consumption and water consumption (Fig. 3k), we used the PC loadings defined above to calculate PC1 and PC2 values for the entire population at each time bin of the PETH (−5 s to +10 s from reward delivery). We followed an analogous procedure to plot neural trajectories during CGRP neuron stimulation. In both cases, we centred every column (time bin).

We repeated this entire analysis using only the neurons from individual mice (Fig. 3l) or using all neurons from a separate group of mice that received CGRP^CEA^ projection stimulation (Extended Data Fig. 9e).

When analysing changes in PC trajectories across days (retrieval in Extended Data Fig. 10b and familiarization in Extended Data Fig. 10k), we followed basically the same procedure as above. For these analyses, we identified PC loadings using only the first day’s (conditioning day or novel day) reward delivery PETHs and then used this set of PC loadings when plotting the PC trajectories for both days. Similarly, we baseline-subtracted and peak-normalized the second day’s PETHs using values that were calculated using only the first day’s PETHs. These measures ensured that PC trajectories were comparable across multiday recordings.

### Acute Neuropixels recordings

#### Surgery.

*Calca*^*cre*^ mice were first injected with 400 nl of AAV5-EF1a-DIO-hChR2(H134R)-eYFP (titre, 1.2 × 10^13^ GC per ml; manufacturer, PNI Viral Core Facility) into the left PB. Four weeks later, in a second surgery, an optical fibre (300 μm diameter core, 0.39 NA) was implanted at a −30° angle above the injection site (see the section ‘Viral injections and optical fibre implantations’ for details), a steel headbar (approximately 1 g) was implanted at AP +1.25 mm, and a ground pin (Newark Electronics) was placed above the right hemisphere of the cerebellum. Finally, a 2 mm^2^ recording chamber was built with Dentin (Parkell S301) above the left hemisphere extending from AP 0 mm to AP −2.0 mm and ML −2.5 mm to ML −3.5 mm. The exposed skull was removed and the brain covered with a silicone elastomer (Kwik-Cast, World Precision Instruments).

#### Recordings.

Mice were habituated to head fixation (3× 30-min sessions). On the recording day, mice were head-fixed in a custom-built recording rig^91^, the silicone elastomer removed and the exposed brain briefly cleaned with normal saline. Neuropixels 1.0 probes^98^ had a soldered connection to short ground to external reference, which was also connected to the mouse’s ground pin during recording. Imme diately before the start of the recording session, the probe was coated in CellTracker CM-DiI (Invitrogen C7000). A single probe was lowered (approximately 10 μm s^−1^) with an ultraprecise micromanipulator (Sensapex μMp) into the amygdala. To prevent drying, the exposed brain and probe shank were covered with a viscous silicone polymer (Dow-Sil, Corning). After reaching the targeted location, the brain tissue was allowed to settle for 15 min before starting the recording. Recordings were acquired at 30 kHz using National Instruments PXI hardware and SpikeGLX software (https://billkarsh.github.io/SpikeGLX; v.3.3). Six recording locations were targeted in each animal. During the recording, mice received 1 s CGRP neuron stimulation followed by a 9 s inter-trial interval for a total of 10 min. Blue light was generated using a 447 nm laser and delivered to the animal using a 200 μm diameter core patch cable. Light power was calibrated to approximately 8 mW at the fibre tip. The laser was controlled with a Pulse Pal signal generator (Sanworks, 1102) programmed to deliver 5 ms laser pulses at 10 Hz.

#### Analysis.

Spike sorting, manual curation and atlas alignment were performed as described above for chronic Neuropixels recordings. To precisely map our electrophysiological data to the anatomical subdivisions of the amygdala in this experiment, we used the IBL’s electrophysiology alignment GUI (https://github.com/int-brain-lab/iblapps/wiki) to manually tune the alignment of each recording to the Allen CCF using electrophysiological landmarks. We then *z* scored each neuron’s 10-ms binned spiking across the entire 10-min CGRP neuron stimulation period. We then generated PETHs surrounding CGRP neuron stimulation trains (−1 s to +2 s), and subtracted the baseline (−1 s to 0 s) mean of each neuron’s PETH (Extended Data Fig. 5c). We then used the built-in Matlab fitgmdist function (CovarianceType=‘diagonal’, RegularizationValue=1e-5, SharedCovariance=false, Replicates=100) to fit a GMM with four response types to this dataset. Specifically, we used the time bins during stimulation (0 s to +1 s) from all amygdala neurons to generate a 3,524 neuron × 100 time bin input matrix for GMM fitting. This GMM revealed two CGRP neuron stimulation-activated response types (shown in green in Extended Data Fig. 5c, left), one CGRP neuron stimulation-inhibited response type (shown in purple) and one unmodulated response type (shown in grey). We then plotted average CGRP neuron stimulation PETHs for each response type separately (Extended Data Fig. 5c, right) and analysed the distribution of CGRP neuron stimulation-activated neurons across amygdala regions (Extended Data Fig. 5d,e).

### GCaMP fibre photometry

We recorded CGRP neuron GCaMP signals (Fig. 2b) with standard fibre photometry acquisition hardware^99,100^. Excitation light was supplied at two wavelengths—isosbestic 405 nm (intensity at patch cable tip, 5–10 μW; sinusoidal frequency modulation, 531 Hz) and activity-dependent 488 nm (intensity, 15–25 μW; sinusoidal frequency modulation, 211 Hz)—using an LED driver (Thorlabs, DC4104) coupled to a low-autofluorescence patch cable (Doric, MFP_400/430/1100–0.57_0.45m_FCM-MF2.5_LAF). Emission light was collected through the same patch cable using a low-light photoreceiver (Newport Femtowatt 215) and then digitized using a base processor (Tucker Davis Technologies, RZ5D) that served both as an analog-to-digital converter and a lock-in amplifier. We then low-pass filtered (2 Hz) and downsampled (100 Hz) the isosbestic 405 nm and activity-dependent 488 nm signals. To control for photobleaching, we applied a linear fit to the isosbestic signal to align it to the activity-dependent signal and then subtracted this fitted isosbestic signal from the activity-dependent signal to obtain the final de-bleached activity-dependent GCaMP signal.

To assess changes in CGRP neuron activity due to LiCl-induced malaise, we generated PETHs using the 10 min before and 30 min after LiCl injection (125 mg kg^−1^ i.p.). We *z* scored the entire PETH for each mouse using the mean and s.d. of the full 10 min before LiCl injection and then downsampled (1 Hz) and smoothed (1-min centred moving average) the final normalized PETHs for plotting.

### AKAR2 fibre photometry

We recorded CEA AKAR2 signals (Fig. 5) using the same acquisition system described above for GCaMP recordings. We low-pass filtered (1 Hz) and downsampled (100 Hz) both the 405 nm and 488 nm signals. Because AKAR2 is a ratiometric indicator of PKA activity^47^, we divided the 488 nm signal by the 405 nm signal to obtain the final de-bleached activity-dependent PKA signal.

This experiment was run on four consecutive days using a version of the two-reward familiarization paradigm described above (Fig. 5b). On day 0 (water day), both ports contained water. On day 1 (novel day), one port (port A) contained the novel sweetened grape Kool-Aid flavour and the other port (port B) contained water. On days 2 and 3 (familiar days), the same ports contained the same sweetened grape Kool-Aid flavour and water as on day 1. The flavour port was counterbalanced across mice.

We generated PETHs surrounding reward delivery (−10 s to +30 s) using the final PKA signal described above. We *z* scored these PETHs separately for each mouse and day using the following procedure. First, we centred each individual reward event PETH by subtracting the mean baseline signal (−5 to −1 s before reward delivery). Then we concatenated this baseline epoch from all individual reward events from both ports for that mouse per day and calculated the s.d. of this vector. Then we divided each individual reward event PETH for that mouse per day by the calculated s.d. Last, we averaged across all rewards of each type (port A, port B) for that mouse per day (Fig. 5d,e).

To quantify PKA activity for statistical analysis, we calculated the average response from +5 to +15 s after reward delivery for each mouse per day per port using the final averaged PETHs calculated above. We then fit a GLMM using the R package glmmTMB^69^ (https://github.com/glmmTMB/glmmTMB; v.1.1.7) with a Gaussian link function and the formula:

(6) PKAactivity~Port*Day+(1|Subject)

where PKA activity is as described above, Port (port A, port B) and Day (day 0, day 1, day 2, day 3) are fixed-effect categorical variables, (1|Subject) is a random effect for each mouse and the asterisk represents the main effects and interactions. Using the coefficients from this GLMM, we used the R package marginaleffects^70^ (https://github.com/vincentarelbundock/marginaleffects; v.0.12.0) to calculate the marginal effect of port (port A – port B) on each day. We used the marginal effect estimates and s.e. values to calculate a *P* value for each day with a *z* test, and then corrected for multiple comparisons across days using the Hochberg–Bonferroni procedure^71^ (Fig. 5f).

The recording location for each animal was determined using the same procedure as for our Neuropixels recordings described above. In brief, we manually annotated the tip of the optical fibre lesion for each animal in the light-sheet microscopy imaging data after registration to the Allen CCF and visualized these recording locations on the Allen CCF (Fig. 5g) as 100 μm circles centred on the fibre tip location for each animal.

### Data exclusions

We excluded data in three instances. First, we excluded one mouse with no hM3D(Gq)–mCherry expression and one mouse with no YFP expression in the LS from Extended Data Fig. 2b. Second, we excluded one mouse with no eOPN3–mScarlet expression in the CEA from Fig. 2f. Third, we excluded confocal images with poor FISH labelling (defined as <35 total *Fos*^+^ CEA cells, or <25% of *Fos*^+^ cells also *Sst*^+^, or <25% of *Fos*^+^ cells also *Prkcd*^+^, or <25% of *Fos*^+^ cells also *Calcrl*^+^) from Fig. 2m,n (22 out of 109 images).

### Statistics and reproducibility

No statistical methods were used to predetermine sample sizes. Sample sizes were chosen based on previous studies investigating CTA and CGRP neurons (for example, refs. 23,24,34) and on the availability of animals. All attempts at replication were successful. Most experiments were replicated in multiple independent groups of animals with all experimental groups present in each cohort. We used multiple independent experimental approaches, and multiple independent analyses within each experiment, to confirm our findings whenever possible. For experiments with multiple groups, individual animals or entire cages were randomly assigned to a group either at the time of surgery or at the beginning of behavioural testing (for animals that did not require surgery) with the constraint of balancing sex across groups. Automated analyses and automated experimental hardware and software, without manual intervention, were used whenever possible. Experimenters were not blinded to the group assignments of the animals. Statistical tests and data analyses were performed in Matlab (R2021a), Python (3.8.10) and R (4.2.1) as described above. *P ≤ 0.05, **P ≤ 0.01, ***P ≤ 0.001, ****P ≤ 0.0001 throughout the article. Individual data points are shown when practical (and always for *n* ≤ 10), and box plots show the data distribution for larger sample sizes. Sample sizes, statistical tests, multiple comparisons corrections, exact *P* values, error bars, shaded areas and box plots are defined in the figure captions, Methods and Supplementary Table 2.

## Extended Data

## Supplementary Material

Supplementary Tables 1 and 2

**Supplementary information** The online version contains supplementary material available at https://doi.org/10.1038/s41586-025-08828-z.

## Data Availability

Data used in this paper are publicly available from Figshare (https://doi.org/10.6084/m9.figshare.28327118)^101^. Source data are provided with this paper.
