# Supplementary material for: A neural mechanism for learning from delayed postingestive feedback
Source: Nature. Author manuscript; Available in PMC 2025 Jun 24. (PMC12176619; doi:10.1038/s41586-025-08828-z)
Supplement: Supplementary Tables 1 and 2 [file NIHMS2082277-supplement-Supplementary_Tables_1_and_2.pdf]

nature portfolio

https://doi.org/10.1038/s41586-025-08828-z

Supplementary information

A neural mechanism for learning from delayed postingestive feedback

In the format provided by the authors and unedited

Supplementary Table 1 | FOS GLMM statistics for individual brain regions.

Summary information for each brain region in the Allen CCF that met the following criteria: (1) total volume ≥0.1 mm^3; (2) lowest level of its branch of the ontology tree (cortical layers not included); and (3) **`Flavour*Time point`** predictors (all main effects and interactions) significantly improved overall FOS GLMM performance (equation (2); see Methods). *P* values are from likelihood-ratio χ^2-tests comparing full and reduced models. Uncorrected *P* values are reported here. The critical *P* value for significance while permitting a 10% FDR using the Benjamini-Krieger-Yekutieli procedure was 0.1172. Of 200 regions that met criteria (1) and (2), 130 also met criterion (3) and are listed below. Novel – familiar ΔFOS values for the consumption, malaise, and retrieval time points were calculated using the GLMM from equation (2), and values for the CGRP stimulation time point were calculated using the GLMM from equation (4).

Name	Abbrev.	Parent	GLMM *P* value	Novel – familiar ΔFOS (*Z*)			
				Consume	Malaise	Retrieval	CGRP stim
Frontal pole	FRP	Cerebral cortex	8.86e-02	-0.58	-0.79	-0.75	-2.97
Secondary motor area	MOs	Cerebral cortex	8.47e-02	-0.87	-2.79	0.06	1.34
Primary somatosensory area, nose	SSp-n	Cerebral cortex	9.30e-02	1.60	-2.34	-0.44	-1.03
Supplemental somatosensory area	SSs	Cerebral cortex	1.13e-01	2.53	-0.72	-0.67	-0.28
Gustatory areas	GU	Cerebral cortex	2.43e-02	2.76	1.69	0.47	2.10
Visceral area	VISC	Cerebral cortex	1.92e-02	2.06	1.52	-0.43	1.23
Dorsal auditory area	AUDd	Cerebral cortex	3.72e-02	0.84	1.18	0.09	0.58
Primary auditory area	AUDp	Cerebral cortex	3.14e-02	-0.30	1.15	0.16	2.23
Posterior auditory area	AUDpo	Cerebral cortex	3.79e-04	-0.91	1.66	0.54	2.11
Ventral auditory area	AUDv	Cerebral cortex	5.89e-03	-0.35	1.10	-0.82	1.59
Anterolateral visual area	VISal	Cerebral cortex	2.21e-03	-0.31	0.05	1.67	1.66
Anteromedial visual area	VISam	Cerebral cortex	8.87e-04	0.18	1.98	1.33	1.69
Lateral visual area	VISl	Cerebral cortex	4.80e-02	0.68	0.06	1.01	2.31
Primary visual area	VISp	Cerebral cortex	1.02e-02	0.92	0.73	1.10	2.33
Posteromedial visual area	VISpm	Cerebral cortex	2.82e-03	0.61	1.26	1.15	1.98
Anterior cingulate area, ventral part	ACAv	Cerebral cortex	2.81e-02	-1.42	0.66	-2.26	1.82
Prelimbic area	PL	Cerebral cortex	5.53e-02	-2.72	-0.66	-1.18	0.09
Infralimbic area	ILA	Cerebral cortex	7.91e-03	-2.38	-1.18	-0.82	1.72
Orbital area, lateral part	ORBl	Cerebral cortex	1.12e-01	1.15	-1.37	-2.26	0.12
Orbital area, medial part	ORBm	Cerebral cortex	1.17e-01	-1.79	-0.93	-1.65	-0.23
Agranular insular area, posterior part	AIp	Cerebral cortex	4.00e-03	3.52	1.48	-0.29	1.60
Retrosplenial area, lateral agranular part	RSPagl	Cerebral cortex	4.33e-03	1.41	0.97	0.94	2.25
Retrosplenial area, dorsal part	RSPd	Cerebral cortex	2.39e-03	0.40	0.86	1.30	2.35
Rostrolateral visual area	VISrl	Cerebral cortex	1.93e-02	0.40	0.50	1.91	1.45
Temporal association areas	TEa	Cerebral cortex	9.24e-04	0.20	1.45	-0.26	1.51
Ectorhinal area	ECT	Cerebral cortex	2.45e-02	-0.50	1.19	0.10	2.20
Anterior olfactory nucleus	AON	Cerebral cortex	1.41e-02	0.29	0.35	-1.45	0.88
Taenia tecta, dorsal part	TTd	Cerebral cortex	8.74e-02	-1.50	-1.08	-2.11	1.92
Dorsal peduncular area	DP	Cerebral cortex	2.32e-03	-1.90	-0.91	-1.51	2.11
Piriform area	PIR	Cerebral cortex	7.53e-02	2.51	-0.14	0.19	1.83
Cortical amygdalar area, anterior part	COAa	Cerebral cortex	7.11e-03	2.01	0.40	2.66	2.32
Piriform-amygdalar area	PAA	Cerebral cortex	7.36e-02	0.97	0.24	2.26	0.97
Postpiriform transition area	TR	Cerebral cortex	4.68e-07	3.36	2.73	4.54	2.26
Field CA1	CA1	Cerebral cortex	1.26e-03	-2.42	0.27	-0.62	1.37
Field CA2	CA2	Cerebral cortex	2.48e-02	-1.66	0.18	-0.32	0.33
Field CA3	CA3	Cerebral cortex	1.64e-04	-1.47	-0.35	-0.86	1.77
Dentate gyrus	DG	Cerebral cortex	4.59e-04	-2.46	-1.19	0.48	1.33
Postsubiculum	POST	Cerebral cortex	1.04e-01	-0.19	-0.55	-0.04	1.40
Endopiriform nucleus, dorsal part	EPd	Cerebral nuclei	2.28e-02	1.61	1.11	0.46	2.15
Endopiriform nucleus, ventral part	EPv	Cerebral nuclei	2.72e-02	1.76	2.43	1.61	1.81
Lateral amygdalar nucleus	LA	Cerebral nuclei	1.31e-02	-1.96	1.19	-0.42	1.19
Basolateral amygdalar nucleus, posterior part	BLAp	Cerebral nuclei	2.05e-06	2.37	3.00	4.79	2.27
Basomedial amygdalar nucleus, anterior part	BMAa	Cerebral nuclei	1.01e-02	1.70	0.79	3.00	2.69
Nucleus accumbens	ACB	Cerebral nuclei	7.31e-03	-3.32	1.86	1.37	1.05
Olfactory tubercle	OT	Cerebral nuclei	1.96e-02	-0.29	-0.69	2.04	-0.64
Lateral septal nucleus	LS	Cerebral nuclei	1.92e-05	-3.92	-0.03	-0.01	0.61

Name	Abbrev.	Parent	GLMM *P* value	Novel – familiar ΔFOS (*Z*)			
				Consume	Malaise	Retrieval	CGRP stim
Septofimbrial nucleus	SF	Cerebral nuclei	1.79e-03	0.22	0.14	-0.18	-0.29
Central amygdalar nucleus, capsular part	CEAc	Cerebral nuclei	3.20e-05	1.89	1.33	2.59	3.85
Central amygdalar nucleus, lateral part	CEAl	Cerebral nuclei	6.21e-06	2.83	1.76	3.08	4.65
Central amygdalar nucleus, medial part	CEAm	Cerebral nuclei	2.18e-06	3.90	2.91	3.23	3.17
Intercalated amygdalar nucleus	IA	Cerebral nuclei	4.33e-05	2.06	2.64	4.30	2.52
Globus pallidus, external segment	GPe	Cerebral nuclei	2.86e-02	-0.06	-1.85	0.79	0.36
Globus pallidus, internal segment	GPi	Cerebral nuclei	3.50e-02	1.22	-1.57	1.44	0.70
Substantia innominata	SI	Cerebral nuclei	3.50e-02	-1.43	1.38	2.31	1.18
Magnocellular nucleus	MA	Cerebral nuclei	1.98e-02	-1.61	-0.19	1.63	1.81
Medial septal nucleus	MS	Cerebral nuclei	1.17e-01	-1.48	0.26	0.44	0.08
Diagonal band nucleus	NDB	Cerebral nuclei	1.15e-01	-0.87	-0.56	1.93	0.77
Triangular nucleus of septum	TRS	Cerebral nuclei	1.14e-03	1.90	0.27	1.44	-0.93
Bed nuclei of the stria terminalis	BST	Cerebral nuclei	6.74e-04	-0.47	0.71	2.84	1.41
Ventral medial nucleus of the thalamus	VM	Thalamus	5.70e-02	-1.41	-0.24	0.10	1.60
Subparafascicular nucleus	SPF	Thalamus	2.87e-02	-0.92	0.81	-0.53	1.31
Subparafascicular area	SPA	Thalamus	3.95e-02	-0.65	0.82	0.99	0.46
Medial geniculate complex	MG	Thalamus	1.07e-01	-0.91	-0.40	2.51	0.92
Lateral geniculate complex	LG	Thalamus	1.12e-03	-3.11	-1.25	1.06	1.42
Lateral posterior nucleus of the thalamus	LP	Thalamus	4.97e-02	-1.64	-0.93	1.69	1.33
Suprageniculate nucleus	SGN	Thalamus	8.79e-02	-0.69	-0.65	2.55	1.09
Anteromedial nucleus	AM	Thalamus	2.89e-02	-1.63	-0.14	1.22	-0.33
Anterodorsal nucleus	AD	Thalamus	2.63e-02	-1.14	-1.80	0.70	-0.91
Intermediodorsal nucleus of the thalamus	IMD	Thalamus	7.63e-04	-1.32	1.83	1.82	0.17
Mediodorsal nucleus of thalamus	MD	Thalamus	1.08e-05	-0.42	2.83	1.83	1.21
Submedial nucleus of the thalamus	SMT	Thalamus	3.56e-03	0.44	1.75	1.35	0.26
Perireunensis nucleus	PR	Thalamus	9.50e-03	-1.01	2.21	-0.11	1.38
Paraventricular nucleus of the thalamus	PVT	Thalamus	2.33e-03	0.45	1.36	1.44	1.31
Parataenial nucleus	PT	Thalamus	6.63e-02	-0.78	1.85	0.41	0.73
Nucleus of reuniens	RE	Thalamus	1.93e-02	0.68	1.38	0.63	1.01
Central medial nucleus of the thalamus	CM	Thalamus	3.40e-02	0.38	0.67	0.92	0.15
Central lateral nucleus of the thalamus	CL	Thalamus	4.58e-03	-1.27	-1.06	-0.51	-0.15
Parafascicular nucleus	PF	Thalamus	1.40e-03	-2.28	-1.16	1.57	0.97
Reticular nucleus of the thalamus	RT	Thalamus	5.95e-06	-2.58	-0.35	3.54	-0.02
Lateral habenula	LH	Thalamus	3.08e-05	-3.91	0.45	1.37	0.36
Paraventricular hypothalamic nucleus	PVH	Hypothalamus	1.10e-02	-0.05	1.83	0.50	1.64
Periventricular hypothalamic nucleus	PV	Hypothalamus	5.27e-02	-1.28	2.00	1.23	2.35
Anterodorsal preoptic nucleus	ADP	Hypothalamus	1.71e-03	-3.28	0.28	0.83	0.91
Anteroventral periventricular nucleus	AVPV	Hypothalamus	5.99e-02	-0.79	0.59	0.73	1.01
Medial preoptic area	MPO	Hypothalamus	1.12e-02	-1.77	0.55	1.28	1.24
Anterior hypothalamic nucleus	AHN	Hypothalamus	2.30e-02	0.42	1.15	0.73	2.10
Medial preoptic nucleus	MPN	Hypothalamus	4.58e-02	-1.17	0.56	1.10	1.34
Ventromedial hypothalamic nucleus	VMH	Hypothalamus	2.09e-02	0.22	1.77	0.43	1.21
Lateral preoptic area	LPO	Hypothalamus	1.27e-02	-1.89	1.40	1.83	0.60
Parasubthalamic nucleus	PSTN	Hypothalamus	2.82e-07	2.38	1.76	2.70	2.45
Subthalamic nucleus	STN	Hypothalamus	3.77e-02	-1.55	-0.21	1.63	2.08
Zona incerta	ZI	Hypothalamus	1.18e-02	-0.75	-1.93	0.32	1.82
Inferior colliculus, central part	ICc	Midbrain	7.55e-02	-2.08	1.02	1.28	4.57
Inferior colliculus, dorsal part	ICd	Midbrain	1.78e-02	-2.67	1.31	0.85	4.83
Ventral tegmental area	VTA	Midbrain	1.35e-03	-0.41	1.65	0.48	3.19
Midbrain reticular nucleus	MRN	Midbrain	7.77e-03	-0.77	-1.48	-0.40	1.71
Periaqueductal gray	PAG	Midbrain	6.01e-03	-0.06	1.59	-1.63	2.21
Anterior pretectal nucleus	APN	Midbrain	5.08e-03	-1.86	-3.05	1.02	0.97
Nucleus of the optic tract	NOT	Midbrain	1.60e-02	-1.02	-1.75	0.06	1.60
Nucleus of the posterior commissure	NPC	Midbrain	1.66e-02	-2.33	-0.25	-0.37	0.60
Posterior pretectal nucleus	PPT	Midbrain	6.65e-03	-1.52	-1.68	0.42	3.65
Cuneiform nucleus	CUN	Midbrain	7.89e-03	-1.27	-0.71	-0.09	2.96
Red nucleus	RN	Midbrain	3.21e-02	-0.77	-2.37	-0.72	1.24
Dorsal nucleus raphe	DR	Midbrain	6.50e-03	-0.20	3.11	0.10	0.84
Parabrachial nucleus	PB	Pons	1.58e-03	0.53	0.82	2.58	NA
Superior olivary complex	SOC	Pons	9.92e-02	0.37	-1.12	2.09	1.53

Name	Abbrev.	Parent	GLMM *P* value	Novel – familiar ΔFOS (*Z*)			
				Consume	Malaise	Retrieval	CGRP stim
Dorsal tegmental nucleus	DTN	Pons	2.85e-02	-0.83	2.14	0.97	0.10
Pontine central gray	PCG	Pons	7.80e-02	-1.00	1.14	0.75	0.09
Supratrigeminal nucleus	SUT	Pons	1.74e-02	0.45	-0.10	1.22	-3.34
Laterodorsal tegmental nucleus	LDT	Pons	3.25e-02	-0.74	2.39	0.85	0.67
Nucleus incertus	NI	Pons	1.94e-02	-2.08	1.14	0.78	-0.38
Pontine reticular nucleus	PRN	Pons	4.63e-02	1.33	-2.23	1.28	2.45
Dorsal cochlear nucleus	DCO	Medulla	4.47e-03	-1.40	0.70	2.39	1.73
Ventral cochlear nucleus	VCO	Medulla	1.84e-02	0.26	0.62	2.43	-1.13
Cuneate nucleus	CU	Medulla	4.81e-02	0.60	-0.01	1.34	1.45
External cuneate nucleus	ECU	Medulla	2.12e-02	-0.12	-0.22	0.68	1.67
Nucleus of the solitary tract	NTS	Medulla	2.02e-03	-0.17	1.48	2.28	1.54
Spinal nucleus of the trigeminal	SPV	Medulla	9.47e-05	-1.49	-0.90	2.51	0.17
Facial motor nucleus	VII	Medulla	1.59e-02	-0.64	0.62	1.77	0.15
Dorsal motor nucleus of the vagus nerve	DMX	Medulla	2.22e-04	1.28	0.54	2.40	1.94
Gigantocellular reticular nucleus	GRN	Medulla	2.86e-02	-1.41	-0.70	2.24	0.92
Intermediate reticular nucleus	IRN	Medulla	2.11e-02	-0.27	0.58	2.92	0.53
Lateral reticular nucleus	LRN	Medulla	1.32e-02	-1.30	0.07	1.35	0.57
Magnocellular reticular nucleus	MARN	Medulla	5.54e-03	-1.47	1.21	1.96	0.99
Medullary reticular nucleus	MDRN	Medulla	3.13e-03	-1.27	1.26	1.70	0.26
Parvicellular reticular nucleus	PARN	Medulla	3.32e-03	-0.97	1.03	2.85	1.21
Paragigantocellular reticular nucleus	PGRN	Medulla	4.36e-03	-1.00	1.04	1.61	-0.03
Nucleus prepositus	PRP	Medulla	1.01e-01	-1.26	-2.13	1.48	1.36
Spinal vestibular nucleus	SPIV	Medulla	4.97e-02	-1.90	-1.24	1.48	-0.18
Hypoglossal nucleus	XII	Medulla	1.48e-03	-0.66	0.29	1.75	0.01

Supplementary Table 2 | Summary of statistical tests.

All statistical tests were two-sided. Paired tests were performed whenever possible. Corrections for multiple comparisons were performed using the Hochberg-Bonferroni procedure. Corrected *P* values are reported here. NS, not significant, *$P \leq 0.05$, **$P \leq 0.01$, ***$P \leq 0.001$, ****$P \leq 0.0001$.

Figure panel	Group	Statistical test	Multiple comparisons	Sample size	Test statistic	*P* value	Sig.
1b, top	Day 1: LiCl vs. Saline	GLMM marginal effect	3 days	*n* = 8 LiCl mice, 8 Saline mice	*Z* = -7.26	8.0e-13	****
1b, top	Day 2: LiCl vs. Saline	GLMM marginal effect	3 days	*n* = 8 LiCl mice, 8 Saline mice	*Z* = -7.59	9.9e-14	****
1b, top	Day 3: LiCl vs. Saline	GLMM marginal effect	3 days	*n* = 8 LiCl mice, 8 Saline mice	*Z* = -6.78	1.2e-11	****
1b, bottom	Day 1: LiCl vs. Saline	GLMM marginal effect	3 days	*n* = 8 LiCl mice, 8 Saline mice	*Z* = -0.56	0.57	NS
1b, bottom	Day 2: LiCl vs. Saline	GLMM marginal effect	3 days	*n* = 8 LiCl mice, 8 Saline mice	*Z* = -1.22	0.57	NS
1b, bottom	Day 3: LiCl vs. Saline	GLMM marginal effect	3 days	*n* = 8 LiCl mice, 8 Saline mice	*Z* = -0.70	0.57	NS
1e	Consume vs. Malaise	Kolmogorov-Smirnov	3 pairs of time points	*n* = 130 regions	*K* = 0.31	1.2e-5	****
1e	Consume vs. Retrieval	Kolmogorov-Smirnov	3 pairs of time points	*n* = 130 regions	*K* = 0.44	3.6e-11	****
1e	Malaise vs. Retrieval	Kolmogorov-Smirnov	3 pairs of time points	*n* = 130 regions	*K* = 0.18	0.021	*
1i	Consume: Novel vs. Familiar	GLMM marginal effect	3 time points	*n* = 12 Novel mice, 12 Familiar mice	*Z* = 3.30	0.0021	**
1i	Malaise: Novel vs. Familiar	GLMM marginal effect	3 time points	*n* = 12 Novel mice, 12 Familiar mice	*Z* = 2.14	0.032	*
1i	Retrieval: Novel vs. Familiar	GLMM marginal effect	3 time points	*n* = 12 Novel mice, 12 Familiar mice	*Z* = 3.28	0.0021	**
2d	Novel vs. Familiar	Wilcoxon rank-sum	N/A	*n* = 6 Novel mice, 6 Familiar mice	*U* = 23	0.0087	**
2e	ChRmine vs. YFP	Wilcoxon rank-sum	N/A	*n* = 6 ChRmine mice, 6 YFP mice	*U* = 21	0.0022	**
2f	eOPN3 vs. YFP	Wilcoxon rank-sum	N/A	*n* = 11 eOPN3 mice, 9 YFP mice	*U* = 144	0.031	*
2h	Novel vs. Familiar	GLMM marginal effect	N/A	*n* = 14 Novel mice, 13 Familiar mice	*Z* = 4.28	1.9e-5	****
2i, top	Malaise vs. CGRP stim	Pearson correlation	N/A	*n* = 12 regions	*r* = 0.809	0.0014	**
2i, bottom	Malaise vs. CGRP stim	Pearson correlation	N/A	*n* = 117 regions	*r* = 0.918	5.2e-48	****
2j, top	Malaise vs. CGRP stim	Pearson correlation	N/A	*n* = 12 regions	*r* = 0.913	3.4e-05	****
2j, bottom	Malaise vs. CGRP stim	Pearson correlation	N/A	*n* = 117 regions	*r* = -0.281	0.0021	**
2m	*Sst*+: Novel vs. Familiar	Wilcoxon rank-sum	N/A	*n* = 6 Novel mice, 7 Familiar mice	*U* = 51	0.23	NS
2m	*Prkcd*+: Novel vs. Familiar	Wilcoxon rank-sum	N/A	*n* = 6 Novel mice, 7 Familiar mice	*U* = 48	0.45	NS
2m	*Calcrl*+: Novel vs. Familiar	Wilcoxon rank-sum	N/A	*n* = 6 Novel mice, 7 Familiar mice	*U* = 49	0.37	NS
2n	*Sst*–/*Prkcd*–/*Calcrl*–: Novel vs. Familiar	Wilcoxon rank-sum	N/A	*n* = 6 Novel mice, 7 Familiar mice	*U* = 34	0.29	NS
2n	*Sst*+/*Prkcd*–/*Calcrl*–: Novel vs. Familiar	Wilcoxon rank-sum	N/A	*n* = 6 Novel mice, 7 Familiar mice	*U* = 41	0.95	NS
2n	*Sst*–/*Prkcd*+/*Calcrl*–: Novel vs. Familiar	Wilcoxon rank-sum	N/A	*n* = 6 Novel mice, 7 Familiar mice	*U* = 35	0.37	NS
2n	*Sst*–/*Prkcd*–/*Calcrl*+: Novel vs. Familiar	Wilcoxon rank-sum	N/A	*n* = 6 Novel mice, 7 Familiar mice	*U* = 35	0.37	NS
2n	*Sst*+/*Prkcd*+/*Calcrl*–: Novel vs. Familiar	Wilcoxon rank-sum	N/A	*n* = 6 Novel mice, 7 Familiar mice	*U* = 44	0.84	NS
2n	*Sst*+/*Prkcd*–/*Calcrl*+: Novel vs. Familiar	Wilcoxon rank-sum	N/A	*n* = 6 Novel mice, 7 Familiar mice	*U* = 42	1.00	NS
2n	*Sst*–/*Prkcd*+/*Calcrl*+: Novel vs. Familiar	Wilcoxon rank-sum	N/A	*n* = 6 Novel mice, 7 Familiar mice	*U* = 39	0.73	NS
2n	*Sst*+/*Prkcd*+/*Calcrl*+: Novel vs. Familiar	Wilcoxon rank-sum	N/A	*n* = 6 Novel mice, 7 Familiar mice	*U* = 53	0.14	NS

Figure panel	Group	Statistical test	Multiple comparisons	Sample size	Test statistic	*P* value	Sig.
3e	Flavour-pref vs. Water-pref	Wilcoxon rank-sum (normal approximation)	3 pairs of neuron groups	*n* = 373 Flavour-pref neurons, 121 Water-pref neurons from 8 mice	*Z* = 3.96	0.00015	***
3e	Flavour-pref vs. Nonselective	Wilcoxon rank-sum (normal approximation)	3 pairs of neuron groups	*n* = 373 Flavour-pref neurons, 610 Nonselective neurons from 8 mice	*Z* = 6.62	1.1e-10	****
3e	Water-pref vs. Nonselective	Wilcoxon rank-sum (normal approximation)	3 pairs of neuron groups	*n* = 121 Water-pref neurons, 610 Nonselective neurons from 8 mice	*Z* = 0.05	0.96	NS
3f	Flavour-pref vs. Water-pref	Wilcoxon rank-sum (normal approximation)	3 pairs of neuron groups	*n* = 373 Flavour-pref neurons, 121 Water-pref neurons from 8 mice	*Z* = 3.84	0.00024	***
3f	Flavour-pref vs. Nonselective	Wilcoxon rank-sum (normal approximation)	3 pairs of neuron groups	*n* = 373 Flavour-pref neurons, 610 Nonselective neurons from 8 mice	*Z* = 7.26	1.2e-12	****
3f	Water-pref vs. Nonselective	Wilcoxon rank-sum (normal approximation)	3 pairs of neuron groups	*n* = 121 Water-pref neurons, 610 Nonselective neurons from 8 mice	*Z* = 0.80	0.42	NS
3n	Flavour-pref vs. Water-pref	Wilcoxon rank-sum (normal approximation)	3 pairs of neuron groups	*n* = 280 Flavour-pref neurons, 80 Water-pref neurons from 4 mice	*Z* = 5.64	3.4e-8	****
3n	Flavour-pref vs. Nonselective	Wilcoxon rank-sum (normal approximation)	3 pairs of neuron groups	*n* = 280 Flavour-pref neurons, 218 Nonselective neurons from 4 mice	*Z* = 5.78	2.2e-8	****
3n	Water-pref vs. Nonselective	Wilcoxon rank-sum (normal approximation)	3 pairs of neuron groups	*n* = 80 Water-pref neurons, 218 Nonselective neurons from 4 mice	*Z* = -2.72	0.0065	**
3q	Flavour-pref vs. Water-pref	Wilcoxon rank-sum (normal approximation)	3 pairs of neuron groups	*n* = 124 Flavour-pref neurons, 20 Water-pref neurons from 4 mice	*Z* = 2.15	0.063	NS
3q	Flavour-pref vs. Nonselective	Wilcoxon rank-sum (normal approximation)	3 pairs of neuron groups	*n* = 124 Flavour-pref neurons, 256 Nonselective neurons from 4 mice	*Z* = -0.34	0.73	NS
3q	Water-pref vs. Nonselective	Wilcoxon rank-sum (normal approximation)	3 pairs of neuron groups	*n* = 20 Water-pref neurons, 256 Nonselective neurons from 4 mice	*Z* = -2.37	0.053	NS
4d, left	CGRP vs. ΔNovel flavour	Pearson correlation	N/A	*n* = 265 neurons from 8 mice	*r* = 0.410	3.4e-12	****
4d, left	CGRP vs. ΔSelectivity	Pearson correlation	N/A	*n* = 265 neurons from 8 mice	*r* = 0.300	6.8e-7	****
4d, middle	CGRP vs. ΔNovel flavour	Pearson correlation	N/A	*n* = 123 neurons from 8 mice	*r* = -0.012	0.89	NS
4d, middle	CGRP vs. ΔSelectivity	Pearson correlation	N/A	*n* = 123 neurons from 8 mice	*r* = 0.059	0.51	NS
4d, right	CGRP vs. ΔNovel flavour	Pearson correlation	N/A	*n* = 551 neurons from 8 mice	*r* = 0.022	0.61	NS
4d, right	CGRP vs. ΔSelectivity	Pearson correlation	N/A	*n* = 551 neurons from 8 mice	*r* = 0.049	0.25	NS
4e	CGRP vs. ΔNovel flavour	Pearson correlation	N/A	*n* = 286 neurons from 8 mice	*r* = 0.124	0.036	*
4e	CGRP vs. ΔSelectivity	Pearson correlation	N/A	*n* = 286 neurons from 8 mice	*r* = 0.194	0.00099	***
4f	Novel vs. Familiar	Wilcoxon signed-rank	N/A	*n* = 201 neurons from 7 mice	*T* = 15142	5.2e-10	****
5f	Day 0: Port A vs. Port B	GLMM marginal effect	4 days	*n* = 13 mice	*Z* = -1.16	0.25	NS
5f	Day 1: Port A vs. Port B	GLMM marginal effect	4 days	*n* = 13 mice	*Z* = 8.74	9.4e-18	****
5f	Day 2: Port A vs. Port B	GLMM marginal effect	4 days	*n* = 13 mice	*Z* = 2.87	0.012	*
5f	Day 3: Port A vs. Port B	GLMM marginal effect	4 days	*n* = 13 mice	*Z* = 2.07	0.078	NS
ED 1a	SSs: Novel vs. Familiar	GLMM marginal effect	3 time points	*n* = 12 Novel mice, 12 Familiar mice	*Z* = 2.53	0.034	*
ED 1a	GU: Novel vs. Familiar	GLMM marginal effect	3 time points	*n* = 12 Novel mice, 12 Familiar mice	*Z* = 2.76	0.017	*
ED 1a	AIp: Novel vs. Familiar	GLMM marginal effect	3 time points	*n* = 12 Novel mice, 12 Familiar mice	*Z* = 3.52	0.0013	**
ED 1a	PIR: Novel vs. Familiar	GLMM marginal effect	3 time points	*n* = 12 Novel mice, 12 Familiar mice	*Z* = 2.51	0.036	*
ED 1a	TR: Novel vs. Familiar	GLMM marginal effect	3 time points	*n* = 12 Novel mice, 12 Familiar mice	*Z* = 3.36	0.0015	**
ED 1a	BLAp: Novel vs. Familiar	GLMM marginal effect	3 time points	*n* = 12 Novel mice, 12 Familiar mice	*Z* = 2.37	0.018	*
ED 1a	CEAl: Novel vs. Familiar	GLMM marginal effect	3 time points	*n* = 12 Novel mice, 12 Familiar mice	*Z* = 2.83	0.0094	**

Figure panel	Group	Statistical test	Multiple comparisons	Sample size	Test statistic	*P* value	Sig.
ED 1a	CEAm: Novel vs. Familiar	GLMM marginal effect	3 time points	*n* = 12 Novel mice, 12 Familiar mice	*Z* = 3.90	0.00029	***
ED 1a	IA: Novel vs. Familiar	GLMM marginal effect	3 time points	*n* = 12 Novel mice, 12 Familiar mice	*Z* = 2.06	0.040	*
ED 1a	PSTN: Novel vs. Familiar	GLMM marginal effect	3 time points	*n* = 12 Novel mice, 12 Familiar mice	*Z* = 2.38	0.035	*
ED 1b	PL: Novel vs. Familiar	GLMM marginal effect	3 time points	*n* = 12 Novel mice, 12 Familiar mice	*Z* = -2.72	0.020	*
ED 1b	CA1 Novel vs. Familiar	GLMM marginal effect	3 time points	*n* = 12 Novel mice, 12 Familiar mice	*Z* = -2.42	0.047	*
ED 1b	DG: Novel vs. Familiar	GLMM marginal effect	3 time points	*n* = 12 Novel mice, 12 Familiar mice	*Z* = -2.46	0.041	*
ED 1b	ACB: Novel vs. Familiar	GLMM marginal effect	3 time points	*n* = 12 Novel mice, 12 Familiar mice	*Z* = -3.32	0.0027	**
ED 1b	LS: Novel vs. Familiar	GLMM marginal effect	3 time points	*n* = 12 Novel mice, 12 Familiar mice	*Z* = -3.92	0.00027	***
ED 1b	LG: Novel vs. Familiar	GLMM marginal effect	3 time points	*n* = 12 Novel mice, 12 Familiar mice	*Z* = -3.11	0.0057	**
ED 1b	RT: Novel vs. Familiar	GLMM marginal effect	3 time points	*n* = 12 Novel mice, 12 Familiar mice	*Z* = -2.58	0.019	*
ED 1b	LH: Novel vs. Familiar	GLMM marginal effect	3 time points	*n* = 12 Novel mice, 12 Familiar mice	*Z* = -3.91	0.00027	***
ED 1b	ADP: Novel vs. Familiar	GLMM marginal effect	3 time points	*n* = 12 Novel mice, 12 Familiar mice	*Z* = -3.28	0.0031	**
ED 1b	ICd: Novel vs. Familiar	GLMM marginal effect	3 time points	*n* = 12 Novel mice, 12 Familiar mice	*Z* = -2.67	0.023	*
ED 2b	hM3D vs. YFP	Wilcoxon rank-sum	N/A	*n* = 18 hM3D mice, 12 YFP mice	*U* = 344	0.0049	**
ED 2d	hM3D vs. YFP	GLMM coefficient estimate	N/A	*n* = 12 Novel mice, 12 Familiar mice	*Z* = 12.69	6.5e-37	****
ED 2e	hM3D vs. YFP	GLMM coefficient estimate	N/A	*n* = 12 Novel mice, 12 Familiar mice	*Z* = -3.42	0.00062	***
ED 2f	Amygdala network vs. Septal complex	One-way analysis of covariance model (slope)	3 pairs of region groups	*n* = 12 amygdala regions, 4 septal regions	*t* = 5.50	6.1e-7	****
ED 2f	Amygdala network vs. Other regions	One-way analysis of covariance model (slope)	3 pairs of region groups	*n* = 12 amygdala regions, 114 other regions	*t* = 3.83	0.00060	***
ED 2f	Septal complex vs. Other regions	One-way analysis of covariance model (slope)	3 pairs of region groups	*n* = 4 septal regions, 114 other regions	*t* = -3.73	0.00088	***
ED 3a	Consume vs. Malaise	Kolmogorov-Smirnov	3 pairs of time points	*n* = 38 regions	*K* = 0.21	0.87	NS
ED 3a	Consume vs. Retrieval	Kolmogorov-Smirnov	3 pairs of time points	*n* = 38 regions	*K* = 0.13	0.87	NS
ED 3a	Malaise vs. Retrieval	Kolmogorov-Smirnov	3 pairs of time points	*n* = 38 regions	*K* = 0.18	0.87	NS
ED 3b	Consume vs. Malaise	Kolmogorov-Smirnov	3 pairs of time points	*n* = 54 regions	*K* = 0.41	0.00031	***
ED 3b	Consume vs. Retrieval	Kolmogorov-Smirnov	3 pairs of time points	*n* = 54 regions	*K* = 0.57	4.2e-8	****
ED 3b	Malaise vs. Retrieval	Kolmogorov-Smirnov	3 pairs of time points	*n* = 54 regions	*K* = 0.28	0.025	*
ED 3c	Consume vs. Malaise	Kolmogorov-Smirnov	3 pairs of time points	*n* = 38 regions	*K* = 0.45	0.0012	**
ED 3c	Consume vs. Retrieval	Kolmogorov-Smirnov	3 pairs of time points	*n* = 38 regions	*K* = 0.71	7.6e-9	****
ED 3c	Malaise vs. Retrieval	Kolmogorov-Smirnov	3 pairs of time points	*n* = 38 regions	*K* = 0.37	0.0081	**
ED 4b	Consume	Wilcoxon signed-rank	3 time points	*n* = 12 regions	*T* = 78	0.00049	***
ED 4b	Malaise	Wilcoxon signed-rank	3 time points	*n* = 12 regions	*T* = 78	0.00049	***
ED 4b	Retrieval	Wilcoxon signed-rank	3 time points	*n* = 12 regions	*T* = 78	0.00049	***
ED 4c	Amygdala cluster vs. Other clusters	Pearson correlation	N/A	*n* = 27 cluster–time points	*r* = 0.758	4.6e-6	****
ED 4d, left	CEA vs. AIp	Pearson correlation	N/A	*n* = 24 mice	*r* = 0.661	0.00044	***
ED 4d, left	CEA vs. BST	Pearson correlation	N/A	*n* = 24 mice	*r* = -0.019	0.93	NS
ED 4d, middle	CEA vs. AIp	Pearson correlation	N/A	*n* = 24 mice	*r* = 0.385	0.063	NS
ED 4d, middle	CEA vs. BST	Pearson correlation	N/A	*n* = 24 mice	*r* = 0.713	9.2e-5	****
ED 4d, right	CEA vs. AIp	Pearson correlation	N/A	*n* = 24 mice	*r* = -0.119	0.58	NS

Figure panel	Group	Statistical test	Multiple comparisons	Sample size	Test statistic	*P* value	Sig.
ED 4d, right	CEA vs. BST	Pearson correlation	N/A	*n* = 24 mice	*r* = 0.676	0.00029	***
ED 6c, top	Malaise vs. CGRP stim	Pearson correlation	N/A	*n* = 12 regions	*r* = 0.712	0.0094	**
ED 6c, bottom	Malaise vs. CGRP stim	Pearson correlation	N/A	*n* = 117 regions	*r* = 0.726	2.0e-20	****
ED 6d, top	Malaise vs. CGRP stim	Pearson correlation	N/A	*n* = 12 regions	*r* = 0.645	0.024	*
ED 6d, bottom	Malaise vs. CGRP stim	Pearson correlation	N/A	*n* = 117 regions	*r* = 0.103	0.27	NS
ED 9c	Flavour-pref vs. Water-pref	Wilcoxon rank-sum (normal approximation)	3 pairs of neuron groups	*n* = 354 Flavour-pref neurons, 129 Water-pref neurons from 8 mice	*Z* = 7.05	3.7e-12	****
ED 9c	Flavour-pref vs. Nonselective	Wilcoxon rank-sum (normal approximation)	3 pairs of neuron groups	*n* = 354 Flavour-pref neurons, 738 Nonselective neurons from 8 mice	*Z* = 8.46	8.1e-17	****
ED 9c	Water-pref vs. Nonselective	Wilcoxon rank-sum (normal approximation)	3 pairs of neuron groups	*n* = 129 Water-pref neurons, 738 Nonselective neurons from 8 mice	*Z* = -2.49	0.013	*
ED 9d	Flavour-pref vs. Water-pref	Wilcoxon rank-sum (normal approximation)	3 pairs of neuron groups	*n* = 354 Flavour-pref neurons, 129 Water-pref neurons from 8 mice	*Z* = 7.01	4.8e-12	****
ED 9d	Flavour-pref vs. Nonselective	Wilcoxon rank-sum (normal approximation)	3 pairs of neuron groups	*n* = 354 Flavour-pref neurons, 738 Nonselective neurons from 8 mice	*Z* = 8.33	2.4e-16	****
ED 9d	Water-pref vs. Nonselective	Wilcoxon rank-sum (normal approximation)	3 pairs of neuron groups	*n* = 129 Water-pref neurons, 738 Nonselective neurons from 8 mice	*Z* = -2.95	0.0031	**
ED 9g	taCasp3 vs. Control	Wilcoxon rank-sum	N/A	*n* = 6 taCasp3 mice, 7 Control mice	*U* = 57	0.033	*
ED 9j	CGRP-activated vs. Other	Wilcoxon rank-sum (normal approximation)	N/A	*n* = 189 CGRP-activated neurons, 632 Other neurons from 4 mice	*Z* = 2.30	0.021	*
ED 10a	Conditioning vs. Retrieval	Wilcoxon signed-rank	N/A	*n* = 8 mice	*T* = 27	0.25	NS
ED 10d	Conditioning vs. Retrieval	Wilcoxon signed-rank	N/A	*n* = 8 mice	*T* = 16	0.82	NS
ED 10f	Conditioning vs. Retrieval	Wilcoxon signed-rank	N/A	*n* = 279 neurons from 4 mice	*T* = 21099	0.20	NS
ED 10g	Conditioning vs. Retrieval	Wilcoxon signed-rank	N/A	*n* = 109 neurons from 4 mice	*T* = 3829	0.012	*
ED 10i	Novel vs. Familiar	Wilcoxon signed-rank	N/A	*n* = 7 mice	*T* = 26	0.047	*
ED 10j	Novel vs. Familiar	Wilcoxon signed-rank	N/A	*n* = 160 neurons from 7 mice	*T* = 4571	0.0020	**
