# Supplementary material for: A neural mechanism for learning from delayed postingestive feedback
Source: Nature. 2025 Apr 2;642(8068):700–9. doi: 10.1038/s41586-025-08828-z (PMC12176619; doi:10.1038/s41586-025-08828-z)
Supplement: Supplementary file 1 [file 41586_2025_8828_MOESM2_ESM.pdf]

nature portfolio

Corresponding author(s): Ilana B. Witten
Christopher A. Zimmerman

Last updated by author(s): 2/2/2025

Reporting Summary

Nature Portfolio wishes to improve the reproducibility of the work that we publish. This form provides structure for consistency and transparency in reporting. For further information on Nature Portfolio policies, see our Editorial Policies and the Editorial Policy Checklist.

Statistics

For all statistical analyses, confirm that the following items are present in the figure legend, table legend, main text, or Methods section.

n/a	Confirmed	
☐	☒	The exact sample size (*n*) for each experimental group/condition, given as a discrete number and unit of measurement
☐	☒	A statement on whether measurements were taken from distinct samples or whether the same sample was measured repeatedly
☐	☒	The statistical test(s) used AND whether they are one- or two-sided<br>*Only common tests should be described solely by name; describe more complex techniques in the Methods section.*
☐	☒	A description of all covariates tested
☐	☒	A description of any assumptions or corrections, such as tests of normality and adjustment for multiple comparisons
☐	☒	A full description of the statistical parameters including central tendency (e.g. means) or other basic estimates (e.g. regression coefficient) AND variation (e.g. standard deviation) or associated estimates of uncertainty (e.g. confidence intervals)
☐	☒	For null hypothesis testing, the test statistic (e.g. *F*, *t*, *r*) with confidence intervals, effect sizes, degrees of freedom and *P* value noted<br>*Give P values as exact values whenever suitable.*
☒	☐	For Bayesian analysis, information on the choice of priors and Markov chain Monte Carlo settings
☒	☐	For hierarchical and complex designs, identification of the appropriate level for tests and full reporting of outcomes
☐	☒	Estimates of effect sizes (e.g. Cohen's *d*, Pearson's *r*), indicating how they were calculated

Our web collection on statistics for biologists contains articles on many of the points above.

Software and code

Policy information about availability of computer code

Data collection

The following software were used for data collection:
MedPC (https://med-associates.com/product/med-pc; version IV)
SpikeGLX (https://billkarsh.github.io/SpikeGLX; version 3.0)
SmartSPIM Acquisition Software (https://lifecanvastech.com/products/smartspim; version 5.6)
LaVision BioTec ImSpector (https://www.lavisionbiotec.com; version 7.0)
OpenEx Software Suite (https://www.tdt.com/component/openex-software-suite; version 2.31)
NDP Scan (https://www.hamamatsu.com; version 3.4)
Leica Application Suite X (https://www.leica-microsystems.com; version 1.8)
Clampex (https://www.moleculardevices.com; version 10.7)

Data analysis

Data were analyzed with publicly available software and with custom MATLAB (R2021a), Python (3.8.10), and R (4.2.1) scripts. Custom code used in this paper is publicly available at https://github.com/cazimmerman/cta and https://github.com/bichanw/cta.

The following software were used for data analysis and visualization:
glmmTMB R package (https://github.com/glmmTMB/glmmTMB; version 1.17)
marginaleffects R package (https://github.com/vincentarelbundock/marginaleffects; version 0.12.0)
Fiji (https://fiji.sc; version 1.52)
TeraStitcher C++ package (https://github.com/abria/TeraStitcher; version 1.11.10)
Pystripe Python package (https://github.com/chunglabmit/pystripe; version 0.2.0)
Elastix C++ package (https://github.com/SuperElastix/elastix; version 4.8)
ClearMap Python package (https://github.com/ChristophKirst/ClearMap2; version 2.0)

Cellfinder Python package (https://github.com/brainglobe/cellfinder; version 0.4.20)
Keras (https://keras.io; version 2.8.0) and TensorFlow (https://www.tensorflow.org; version 2.8.0)
cloudvolume Python package (https://github.com/seung-lab/cloud-volume; version 8.5.1)
Violinplot MATLAB package (https://github.com/bastibe/Violinplot-Matlab)
Cellpose (https://cellpose.readthedocs.io; version 3.0.8)
ABBA (https://abba-documentation.readthedocs.io; version 0.8.0)
CatGT (https://billkarsh.github.io/SpikeGLX; version 3.3)
IBL Python Kilosort 2.5 implementation (https://github.com/int-brain-lab/pykilosort)
Phy Python package (https://github.com/cortex-lab/phy; version 2.0)
IBL electrophysology alignment GUI (https://github.com/int-brain-lab/iblapps/wiki)
Brainreg Python package (https://github.com/brainglobe/brainreg; version 0.4.0)
Brainreg-segment Napari module (https://github.com/brainglobe/brainreg-segment; version 0.2.16)
Spikes MATLAB package (https://github.com/cortex-lab/spikes)
scikit-learn Python package (https://scikit-learn.org; version 1.0.2)

For manuscripts utilizing custom algorithms or software that are central to the research but not yet described in published literature, software must be made available to editors and reviewers. We strongly encourage code deposition in a community repository (e.g. GitHub). See the Nature Portfolio guidelines for submitting code & software for further information.

Data

Policy information about availability of data

All manuscripts must include a data availability statement. This statement should provide the following information, where applicable:

- Accession codes, unique identifiers, or web links for publicly available datasets
- A description of any restrictions on data availability
- For clinical datasets or third party data, please ensure that the statement adheres to our policy

Data used in this paper are publicly available at https://doi.org/10.6084/m9.figshare.28327118.

The original Allen CCFv3 (Wang et al. Cell 2020) is available at: https://atlas.brain-map.org

Research involving human participants, their data, or biological material

Policy information about studies with human participants or human data. See also policy information about sex, gender (identity/presentation), and sexual orientation and race, ethnicity and racism.

Reporting on sex and gender	N/A
Reporting on race, ethnicity, or other socially relevant groupings	N/A
Population characteristics	N/A
Recruitment	N/A
Ethics oversight	N/A

Note that full information on the approval of the study protocol must also be provided in the manuscript.

Field-specific reporting

Please select the one below that is the best fit for your research. If you are not sure, read the appropriate sections before making your selection.

☒ Life sciences ☐ Behavioural & social sciences ☐ Ecological, evolutionary & environmental sciences

For a reference copy of the document with all sections, see nature.com/documents/nr-reporting-summary-flat.pdf

Life sciences study design

All studies must disclose on these points even when the disclosure is negative.

Sample size	No statistical methods were used to predetermine sample sizes. Sample sizes were chosen based on previous studies investigating CTA and CGRP neurons (for example, Carter et al. Nature 2013; Carter et al. J Neurosci 2015; Chen et al. Neuron 2018) and on the availability of animals.
Data exclusions	We excluded data in three instances. First, we excluded one mouse with no hM3D(Gq)-mCherry expression and one mouse with no YFP expression in the LS from Extended Data Fig. 2b. Second, we excluded one mouse with no eOPN3-mScarlet expression in the CEA from Fig. 2f. Third, we excluded confocal images with poor FISH labeling (defined as <35 total Fos+ CEA cells, or <25% of Fos+ cells also Sst+, or <25% of Fos+ cells also Prkcd+, or <25% of Fos+ cells also Calcrl+) from Fig. 2m,n (22 out of 109 images).

Replication	All attempts at replication were successful. Most experiments were replicated in multiple independent cohorts of animals with all experimental groups present in each cohort. We used multiple independent experimental approaches, and multiple indpendent analyses within each experiment, to confirm our findings whenever possible.<br>— The behavioral experiments involving LiCl-induced malaise, CGRP neuron stimulation (one cohort with CGRP neuron cell-body stimulation, one cohort with CGRP–CEA projection stimulation), CGRP–CEA projection inhibition, CGRP neuron ablation, and LS activation each included two independent cohorts of mice with consistent results.<br>— The brainwide Fos imaging experiments included two independent cohorts of mice for each experimental timepoint (Consumption, Malaise, Retrieval, CGRP neuron stimulation, LS activation) with consistent results.<br>— The RNAscope experiment included two independent cohorts of mice with consistent results.<br>— The CGRP neuron photometry experiment included two independent cohorts of mice with consistent results.<br>— The CGRP neuron stimulation and CGRP–CEA projection stimulation Neuropixels experiments each included two independent cohorts of mice with consistent results. These results were further corroborated by the independent LiCl-induced malaise Neuropixels experiments.<br>— The familiarization Neuropixels experiment included two independent cohorts of mice with consistent results.<br>— The PKA fiber photometry experiment included two groups of mice ("rear" and "front" flavor location groups) run as a single cohort with consistent results across groups. These results were further corroborated by an independent pilot cohort.<br>— The results of the acute CGRP neuron stimulation Neuropixels experiment (n = 24 insertions from 4 mice in one cohort) were consistent across animals. These results were further corroborated by the independent CGRP neuron stimulation brainwide Fos experiment.
Randomization	For experiments with multiple groups, individual animals or entire cages were randomly assigned to a group either at the time of surgery or at the beginning of behavioral testing (for animals that did not require surgery) with the constraint of balancing sex across groups.
Blinding	Automated analyses without manual intervention were used whenever possible (for example, cell detection and downstream analysis for brainwide light sheet imaging data, spike sorting and downstream analysis of Neuropixels data). Automated experimental hardware and software were used whenever possible (for example, reward delivery and laser timing in the CFA paradigm). Group identity during LiCl malaise and CGRP neuron stimulation was typically obvious based on behavioral change, and thus experimenters were not blinded to the group assignments of the animals. The experimenter was blinded to the anatomical location and task-tuning of units during manual curation of spike sorting output. The experimenter was blinded to group identity while manually classifying RNAscope FISH data.

Reporting for specific materials, systems and methods

We require information from authors about some types of materials, experimental systems and methods used in many studies. Here, indicate whether each material, system or method listed is relevant to your study. If you are not sure if a list item applies to your research, read the appropriate section before selecting a response.

Materials & experimental systems

n/a	Involved in the study	
☐	☒	Antibodies
☒	☐	Eukaryotic cell lines
☒	☐	Palaeontology and archaeology
☐	☒	Animals and other organisms
☒	☐	Clinical data
☒	☐	Dual use research of concern
☒	☐	Plants

Methods

n/a	Involved in the study	
☒	☐	ChIP-seq
☒	☐	Flow cytometry
☒	☐	MRI-based neuroimaging

Antibodies

Antibodies used	mouse anti-CGRP (Abcam ab81887)<br>rabbit anti-GFP (Novus NB600-308)<br>chicken anti-GFP (Aves GFP-1020)<br>rabbit anti-Fos (Synaptic Systems 226008)<br>Alexa Fluor 594 donkey anti-rabbit (Jackson Immuno 703-585-155)<br>Alexa Fluor 568 donkey anti-mouse (Life Technologies A10037)<br>Alexa Fluor 647 donkey anti-rabbit (Abcam ab150075, Invitrogen A31573)
Validation	Primary antibodies were validated by the manufacturer.<br>mouse anti-CGRP: https://www.abcam.com/en-us/products/primary-antibodies/cgrp-antibody-4901-ab81887<br>rabbit anti-GFP: https://www.novusbio.com/products/gfp-antibody_nb600-308<br>chicken anti-GFP: https://www.aveslabs.com/products/anti-green-fluorescent-protein-antibody-gfp<br>rabbit anti-Fos: https://www.sysy.com/product/226008

Animals and other research organisms

Policy information about studies involving animals; ARRIVE guidelines recommended for reporting animal research, and Sex and Gender in Research

Laboratory animals	Wild type mice (JAX 00064) and Calca::Cre mice (JAX 033168) were obtained from the Jackson Laboratory. Adult mice (>8 weeks)

Laboratory animals	were used for all experiments. Mice were housed under a 12-h light–dark cycle, and experiments were conducted during the dark cycle. Ambient temperature was maintained at 21–26 °C and humidity at 30–70%.
Wild animals	No wild animals were used.
Reporting on sex	Mice of both sexes were used for all experiments.
Field-collected samples	No field-collected samples were used.
Ethics oversight	All experimental procedures were approved by the Princeton University Institutional Animal Care and Use Committee following the NIH Guide for the Care and Use of Laboratory Animals.

Note that full information on the approval of the study protocol must also be provided in the manuscript.
